# Design of a novel multi-epitope vaccine candidate against hepatitis C virus using structural and nonstructural proteins: An immunoinformatics approach

**DOI:** 10.1371/journal.pone.0272582

**Published:** 2022-08-30

**Authors:** Esmaeil Behmard, Hussein T. Abdulabbas, Saade Abdalkareem Jasim, Sohrab Najafipour, Abdolmajid Ghasemian, Akbar Farjadfar, Ebrahim Barzegari, Amin Kouhpayeh, Parviz Abdolmaleki

**Affiliations:** 1 School of Advanced Technologies in Medicine, Fasa University of Medical Sciences, Fasa, Iran; 2 Department of Medical Laboratory Techniques, Faculty of Health and Medical Techniques, Imam Ja’afar Al-Sadiq University, Al Muthanna, Iraq; 3 Medical Laboratory Techniques Department, Al-maarif University College, Ramadi, Iraq; 4 Noncommunicable Diseases Research Center, Fasa University of Medical Sciences, Fasa, Iran; 5 Department of Medical Biotechnology, Fasa University of Medical Sciences, Fasa, Iran; 6 Medical Biology Research Center, Health Technology Institute, Kermanshah University of Medical Sciences, Kermanshah, Iran; 7 Department of Pharmacology, Fasa University of Medical Sciences, Fasa, Iran; 8 Department of Biophysics, Faculty of Biological Sciences, Tarbiat Modares University, Tehran, Iran; Rajendra Memorial Research Institute of Medical Sciences, INDIA

## Abstract

Hepatitis C virus (HCV) infects the liver and causes chronic infection. Several mutations in the viral genome have been associated with drug resistance development. Currently, there is no approved vaccine against the HCV. The employment of computational biology is the primary and crucial step for vaccine design or antiviral therapy which can substantially reduce the duration and cost of studies. Therefore, in this study, we designed a multi-epitope vaccine using various immunoinformatics tools to elicit the efficient human immune responses against the HCV. Initially, various potential (antigenic, immunogenic, non-toxic and non-allergenic) epitope segments were extracted from viral structural and non-structural protein sequences using multiple screening methods. The selected epitopes were linked to each other properly. Then, toll-like receptors (TLRs) 3 and 4 agonists (50S ribosomal protein L7/L12 and human β-defensin 2, respectively) were added to the N-terminus of the final vaccine sequence to increase its immunogenicity. The 3D structure of the vaccine was modeled. Molecular dynamics simulations studies verified the high stability of final free vaccines and in complex with TLR3 and TLR4. These constructs were also antigenic, non-allergenic, nontoxic and immunogenic. Although the designed vaccine traits were promising as a potential candidate against the HCV infection, experimental studies and clinical trials are required to verify the protective traits and safety of the designed vaccine.

## 1. Introduction

Hepatitis C virus (HCV) is one of the most important human infectious diseases threatening the life of a large part of the international community [1–3]. The World Health Organization (WHO) has estimated that ~200 million cases are infected with the HCV worldwide, and 3–4 million new cases are diagnosed each year [4–6]. Liver cirrhosis and carcinoma are major consequences of chronic HCV infection. The treatment efficacy is insufficient due to various side effects of anti-HCV drugs and the development of drug resistance by the virus [7–10]. Therefore, there is an unmet requirement to develop new strategies such as vaccine design to reduce the HCV incidence and related mortality.

It is well known that a successful vaccination should be associated with sufficient immunity (through elicit of immune responses) and protection [11, 12]. Various vaccine candidates have been developed against the HCV [13–15]. The design of a multi-epitope vaccine derived from the structural and non-structural protein sequences is promising and preferable than whole cell or inactivated candidates against viral pathogens [16–20]. These proteins play a major role in the process of viral penetration and replication within the host cell [9, 10]. Multi-epitope vaccines advantages over the inactivated or whole protein vaccines include lower costs and rapidity of production, higher immunity and safety potential, lower non-specific reactions or cross-reactivity and convenient manipulation [21–23]. Identification of potential epitopes capable of eliciting immune responses have become a challenge in current safety research [17, 18]. Application of basic structure design techniques and immunoinformatics tools in hybrid with dynamic mechanistic studies are promising to profoundly understand these mechanisms [16–18]. Among these methods, computational techniques draw attentions increasingly, because of their contribution to remarkable reduction of time and costs of vaccine design [14–20, 24]. With the advent of sequencing technologies, sufficient genomic and proteomic information have been provided from various viruses. As a result, peptide-based and epitope-based vaccines can be designed with the help of various bioinformatics tools [14–20, 24]. The design of epitope-based vaccines is now a familiar concept. Epitope-based vaccines against *Leishmania donovani* complex [15], Theileria parasites [16], Chikungunya virus [17], Human Herpes virus [18], human immunodeficiency virus (HIV) [19], Ebola virus [20], and human coronavirus [22] have been suggested to date. For successful development of peptide-based vaccines understanding the molecular details of antigen detection is an important and effective step. Though the identification of immunogenic epitopes has been facilitated by various algorithms, more computational studies are needed to prove the tendency and detail of interactions between the immune receptor and epitopes. Considering this, docking and molecular dynamics simulation services are effective sources of molecular-level structural information in immunology. Various Toll-like receptors (TLRs) such as TLR2, TLR3, TLR4 and TLR6 act as receptors of the viral proteins [25]. The membrane TLR4 and intracellular TLR3 agonist/adjuvant has been adopted for ligand and receptor interaction assessment in some studies of multi-epitope vaccine design [21, 22]. TLR3 plays a crucial role in the antiviral induction of hepatic macrophages and Kupffer cells via nuclear factor kappa B (NF-KB) pathway and pro-inflammatory cytokines CXCL10. TLR4 agonist also provokes substantial immune responses [26].

In this study, a multi-epitope vaccine was designed against the HCV using immunoinformatics and computational methods from sequences of HCV structural and non-structural proteins.

## 2. Materials and methods

**Fig 1:**
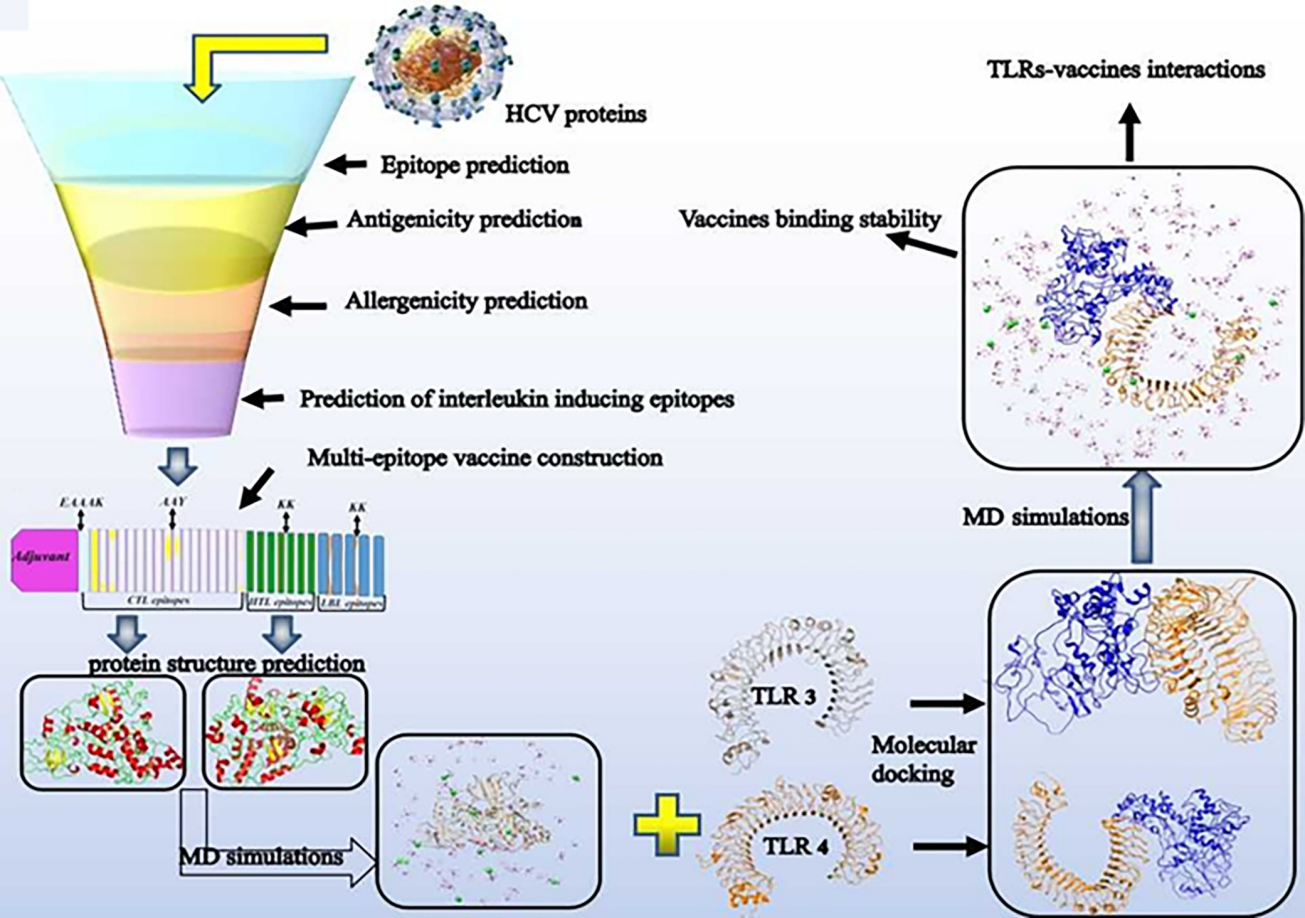
Systemic flowchart of the multi-epitope subunit vaccines build against HCV.

The basic steps of designing a multi-epitope vaccine are shown in the Fig 1.

### 2.1. Viral protein sequences

For designing multi-epitope vaccine constructs against HCV, both structural and non-structural protein sequences were obtained from protein databases. The occurrence of high mutation rate in the HCV genome in infected populations facilitates immune escape and adaptation of the virus. Therefore, to obtain a vaccine active against wide range of viral strains, alignment of the viral protein sequences from various strains is needed to gain conserved sequences in order to select efficient candidate epitopes. To this aim, clustalW2 was used to perform multiple sequence alignment [27].

### 2.2. Prediction of MHC-І binding (Cytotoxic T Lymphocyte (CTL)) epitopes

Predicting peptides that are capable of inducing T-cell responses is a crucial step in the design of epitope-based peptide vaccine. The NetCTL v1.2 server (www.cbs.dtu.dk/services/NetCTL) was applied to predict the CD8+ T-cell epitopes. In the IEDB MHC class I binding tool (www.iedb.org), the Artificial Neural Network and human were selected as prediction method and MHC source species, respectively. The IC50 value was selected 50 nM. In the NetCTL v1.2 server, the threshold value was selected as 0.5.

### 2.3. Prediction of MHC-ІІ binding (Helper T Lymphocyte (HTL)) epitopes

The IEDB tool was used to predict HTL epitopes in structural and non-structural proteins of the HCV. Then, HTL epitopes with IC50 value of less than 50 nM were selected for further use.

### 2.4. Prediction of linear B-cell epitopes

As we know, one of the important factors in the process of immunization of the human body against pathogens is the secretion of antibodies by B lymphocytes, participating in the humoral immunity. In this study, BCPred server (http://ailab.ist.psu.edu/bcpred) was utilized to predict 20-mer B-cell epitopes, using a support vector machine (SVM) algorithm and a kernel technique.

### 2.5. Estimation of antigenicity, allergenicity, and toxicity of CTL, HTL and B cells epitopes

The antigenic potential of CTL, HTL and B cells epitopes was predicted using the VaxiJen v2.0, and epitopes with antigenic values of more than 0.4 were selected for further screening [28]. The toxicity and allergenicity of these selected epitopes were checked by the ToxinPred and the AllerTOP servers, respectively [29, 30]. Subsequently, the ability to induce the secretion of various interleukins (interferon-gamma (IFN-γ), interleukin-4 (IL-4) and interleukin-10 (IL-10) secreted by B and HTL (CD4^+^) cells epitopes was checked through the IFNepitope, IL4pred and IL10pred server tools [31–33]. Among the predicted epitopes, those with high antigenic potential, non-allergenicity and non-toxicity and high solubility at high expression levels were selected to develop a multi-epitope vaccine.

### 2.6. Designing of multi-epitope vaccine construct

The multi-epitope vaccine was designed via fusion of CTL, HTL and B cells epitopes with appropriate linkers (AAY and KK) according to recent studies [15–19]. Two separate adjuvants were also added to the N termini to increase the immunogenicity of the multi-epitope vaccines [15–19]. To this aim, a 124- and a 45-amino acid sequences from 50S ribosomal protein L7/L12 (TLR4 agonist) and human β-defensin 2 (TLR3 agonist) were respectively obtained and added separately to the N terminus of the vaccine sequence using the appropriate linker (EAAAK) [15]. The vaccines designed in this route contained 686 and 607 residues, respectively.

### 2.7. Estimation of immunogenic, allergenic and physiochemical properties of multi-epitope vaccines

The VaxiJen v2.0 and SolPro (http://scratch.proteomics.ics.uci.edu/) web tools were used to predict the antigenic potential and solubility of the multi-epitope vaccines, respectively [28]. The allergenicity of the vaccines was checked using AllerTOP server [29]. In addition, the physiochemical properties of the vaccines, such as amino acid composition, theoretical isoelectric point (pI), grand average of hydropathicity (GRAVY), molecular weight, aliphatic and instability index, and *in vitro* and *in vivo* half-life were evaluated using ProtParam server [34].

### 2.8. Modeling the three-dimensional structure of the multi-epitope vaccines

The secondary structural properties of the multi-epitope vaccine were analyzed using the SOPMA secondary structure prediction method [35]. Modeling and refinement of the three-dimensional (3D) structure of the multi-epitope vaccines was performed using the Galaxy server (http://galaxy.seoklab.org/). The Galaxy server relaxes the model structure using repacking and molecular dynamics simulation. The 3D structure of the multi-epitope vaccines was then validated using a variety of tools, such as SWISS-MODEL server [36], ProSA-web [37] and ERRAT (https://servicesn.mbi.ucla.edu/).

### 2.9. Molecular dynamics simulation of multi-epitope vaccines

High-performance MD simulation is an important method to validate the stability of the modeled structures. The multi-epitope vaccines and vaccine-TLRs complexes were simulated for 100 ns using Gromacs 2020 package [38], implementing similar protocols as in our previous studies [39]. The simulation trajectories were saved for the multi-epitope vaccines and complexes every 10 ps and root mean square deviations (RMSDs), root mean square fluctuations (RMSFs) and radius of gyration (Rg) analyses were carried out by Gromacs tools box. The final structures of multi-epitope vaccines were used as a ligand for docking to TLR3 and TLR4.

### 2.10. Molecular docking of toll-like receptors and multi-epitope vaccines

The molecular docking of the final vaccines after 100 ns simulations with TLR3 (PDB ID: 2A0Z) and TLR4 (PDB ID: 4G8A, www.rcsb.org) was performed using the ClusPro 2.0 web server [40] to analyze the binding pattern of vaccines with TLR3 and TLR4. Based on the lowest global energy, the final vaccine-TLRs complexes models were selected and visualized using PyMOL package and LIGPLOT software [41, 42].

## 3. Results

### 3.1. Sequence alignment

The alignment of various HCV protein sequences was performed using ClustalW2 (https://embnet.vital-it.ch/software/ClustalW.html). The results of this step has been shown in supplementary data.

### 3.2. Prediction and evaluation of HTL, CTL and B cell epitopes

The IEDB MHC-II prediction tool, the NetCTL 1.2 and the BCPred servers were used to predict HTL (15-mer), CTL and B-cell linear epitopes of the HCV proteins, respectively (S1–S9 Tables). The potentially antigenic and non-allergenic epitopes (using VaxiJen v2.0 and AllerTOP v.2.0, respectively) were adopted and next, those induced various cytokines such as IFN-γ, IL-4, and IL-10 (via prediction of B and HTL epitopes) were obtained (Table 1).

**Table 1:** Final selected CTL, HTL and LBL epitopes for multi-epitope vaccines construction.

Protein	CD8+-T cell epitope	CD4+-T cell epitope	B-cell epitope
P7	^42^YAIYGTWPL	^36^LVPGMTYAIYGTWPLLLL	^32^IKGRLVPGMTYAIYGTWPLL
	^54^LLALPQRAY		
NS3	^432^VTQTVDFSLDPTFTI		
	^126^LLSPRPVSY		
	^204^APTGSGKST		
	^362^GRHLIFCHSKKK	^500^ AWYELTPAETTVRLR	^587^RLKPTLRGPTPLLY
	^123^RGSLLSPRPVSYLK	^386^INAVAYYRGLDVSVI	^181^SPTFTDNSTPPAVP
	^107^VTRHADVIPV	^534^SVFTGLTHIDAHFLSQT	^86^PAPQGTRSLTPCTC
	^383^GLGINAVAY	^554^GDNFPYLVAYQATVCARAQAP	
	^262^GAPITYSTY		
	^230^PSVAATLGFGAYMSKAH		
NS5B	^454^IEPLDLPQI	^560^IYHSLSRARPRWFMW	^141^KSEVFCVQPEKGGR
	^489^LRKLGVPPLR		
	^132^TPIDTTIMAKSEVFCVQPEK		
	^560^IYHSLSRARPRWFMWCLLLLSVGVGIYL	^35^NMVYATTSRSASLRQ	
	^500^WRHRARSVRARLLSQGGRAATCGKYLFNWAVRTKLKLTPI		
	^199^QRVEFLVNAWKSKKSPMGFSYDTRCFDSTVTESDIRV		
	^29^SLLRHHNMVYATTRSASLRQKKVTF		

### 3.3. Combining selected epitopes to make the final multi-epitope vaccine

**Fig 2:**
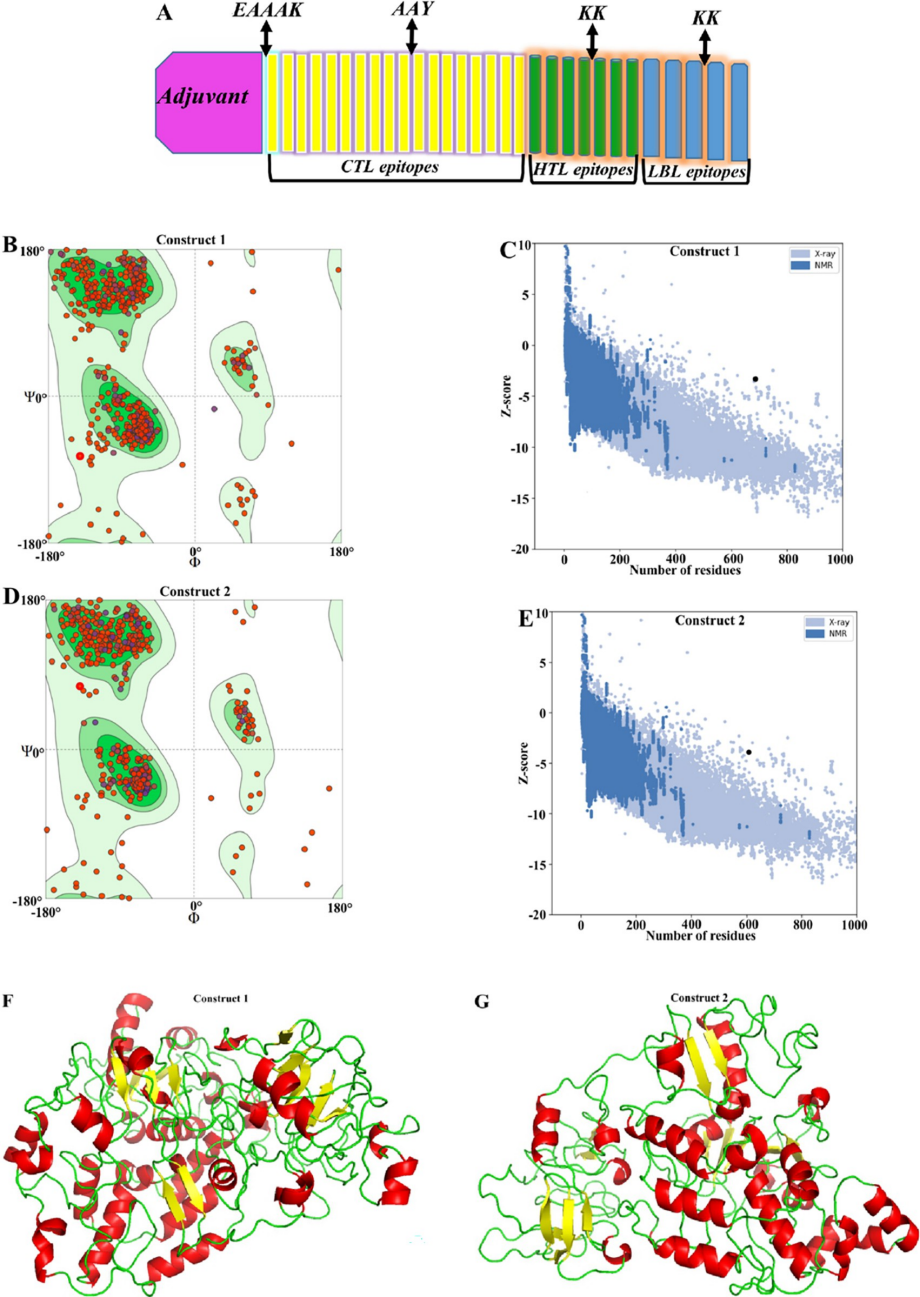
Prediction and assessment of the 3D structure of the multi-epitope vaccines. (A) Schematic image of the final vaccine. (B) Ramachandran plot analysis of refined construct 1, showing 91.08%, 7.17% and 1.75% residues in favored, allowed and disallowed region, respectively, (C) ProSA validation of 3D construct 1, showing Z-score -3.28, (D) Ramachandran plot analysis of refined construct 2, showing 91.07%, 7.28% and 1.65% residues in favored, allowed and disallowed region, respectively, (E) ProSA validation of 3D construct 2, showing Z-score -3.88, (F and G) 3D structure of vaccines models showing α-helix (red cartoon), β-strand (yellow cartoon) and loop (green cartoon).

Selected potential epitopes including 18 CTL, 7 HTL and 5 LBL epitopes were fused together using AAY and KK linkers, respectively (Fig 2A). The 50S ribosomal protein L7/L12 (TLR4 agonist) and human β-defensin 2 (TLR3 agonist) adjuvants were separately added to the N terminal part sequence of the final multi-epitope vaccine using an EAAAK linker to increase immunogenicity (Fig 2A) giving final multi-epitope vaccines sequences with 686 and 607 residues (S1 Fig).

### 3.4. Evaluation of antigenicity, allergenicity, physiochemical properties and secondary structure of the vaccine constructs

The designed vaccines were sufficiently antigenic and non-allergenic. Their molecular weight included 76.27 kDa and 67.87 kDa, and their theoretical pI included 9.98 and 10.16 (Table 2). The instability and the aliphatic indices of the final vaccines have been represented in the Table 2. The final vaccines have a grand average of hydropathicity (GRAVY) values of -0.152 and -0.169 (Table 2 and S1 Fig).

**Table 2:** Various features of multi-epitope vaccines.

Features	Assessment	
	Construct 1	Construct 2
Adjuvant	50S ribosomal protein L7/L12 (TLR4)	Human β-defensin 2 (TLR3)
Number of amino acids	686	607
Molecular weight	76270.36 Dalton	67872.61 Dalton
Chemical formula	C3496H5551N933O938S19	C3104H4887N851O811S24
Theoretical pI	9.98	10.16
Total number of negatively charged residues (Asp + Glu):	43	24
Total number of positively charged residues (Arg + Lys)	107	97
Total number of atoms	10937	9677
Extinction coefficient (at 280 nm in H2O)	126060 M-1cm-1	127550 M-1cm-1
Instability index	35.01	38.13
Aliphatic index	89.55	81.76
Grand average of hydropathicity (GRAVY)	-0.152	-0.169
Antigenicity	0.53 (AntigenPro), 0.75 (Vaxijen v.2.0)	0.80 (AntigenPro), 0.78(Vaxijen v.2.0)
Allergenicity	Probable non-allergen (AllergenFP v.1.0)	Probable non-allergen (AllergenFP v.1.0)
	Probable non-allergen (AllerTOP v.2.0)	Probable non-allergen (AllerTOP v.2.0)

### 3.5. Modeling, refinement, and validation of vaccine constructs

**Fig 3:**
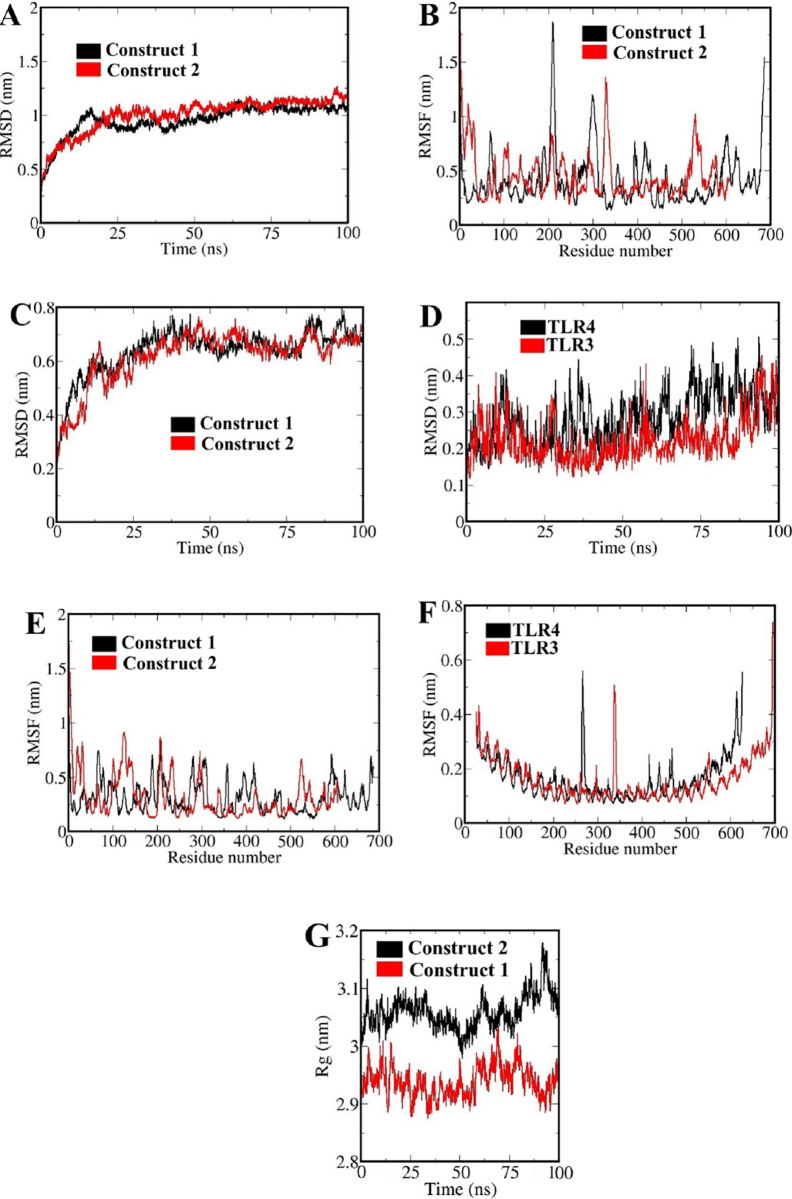
MD simulations outputs of free vaccines and vaccines-TLRs complexes; A and B: The RMSD and RMSF values of Cα atoms for free vaccines, respectively; C and D: The RMSD values of Cα atoms for vaccines and TLRs in the complex states, respectively; E and F: The RMSF values of Cα atoms for vaccines and TLRs in the complex states, respectively; G: The Rg values of vaccines structures in the complex states.

The three-dimensional structures of the final vaccines were modeled and refined (Fig 2). In the optimal structural model of construct 1, 91.08% of the residues were in the favored area, 7.17% in the allowed area and 1.75% in the disallowed area of Ramachandran plot (Fig 2B). In addition, the quality factor obtained from the ERRAT analysis was 76.41% and the Z-score was −3.28 (Fig 2C). Regarding the construct 2, 91.07% of the residues were in the favored area, 7.28% in the allowed area and 1.65% in the disallowed area of Ramachandran plot (Fig 2D). In addition, the quality of 72.64% and the Z-score of −3.88 was obtained (Fig 2E). The ERRAT score included 76.41% and 72.64% for the vaccine candidates (Fig 3F and 3G).

### 3.6. Prediction of B-cell epitopes in the final vaccines

In general, four discontinuous B-cell epitopes with score of 0.83 to 0.87, and 12 linear B-cell epitopes with score of 0.83 to 0.93 were selected as the final epitopes in 50S ribosomal protein L7/L12-multi-epitope vaccine (S2 Fig, Tables 3 and 4). In addition, six discontinuous B-cell epitopes with score range of 0.82 to 0.88, and 14 linear B-cell epitopes with score range of 0.83 to 0.91 were selected as the final epitopes in human β-defensin 2-multi-epitope vaccine (S2 Fig, Tables 3 and 4). For both multi-epitope vaccines, the PI values of 0.87 and 0.88 demonstrated that 87% and 88% of residues located in the predicted ellipsoid area of the epitopes and these epitopes had the highest solvent accessibility.

**Table 3:** Linear B-cell epitopes of the final vaccines.

	Linear B cell epitopes	Score
Construct 1	^250^AAYGAPITYS^259^	0.93
Construct 1	^306^RAAYTPID^313^	0.91
Construct 1	^62^LKGAGS^67^	0.91
Construct 1	^70^LTVVKRIKDLIGL^82^	0.90
Construct 1	^278^SKAHAAYIEPLDL^290^	0.90
Construct 1	^614^AIYGTWPLLKKRLKPTLRGPT^634^	0.89
Construct 1	^645^TDNSTPP^651^	0.88
Construct 1	^562^ARAQAPK^568^	0.88
Construct 1	^45^KAEILDKS^52^	0.87
Construct 1	^589^VYATTS^594^	0.85
Construct 1	^356^VGIYLAAYW^364^	0.85
Construct 1	^606^RLVPGM^611^	0.83
Construct 2	^1^GIINTLQKYYCRVRGGRC^18^	0.91
Construct 2	^521^RQKKKGRLVPGMTYAIYGTW^540^	0.89
Construct 2	^199^SKAHAAYIEPLDLPQIA^215^	0.88
Construct 2	^225^PLRAAYTPI^233^	0.86
Construct 2	^237^IMAKSE^242^	0.86
Construct 2	^94^PVSYAAYAPTGSGKSTA^114^	0.85
Construct 2	^572^PAVPKKPA^579^	0.85
Construct 2	^549^PTLRG^553^	0.84
Construct 2	^339^SKKS^342^	0.82
Construct 2	^511^YATTS^515^	0.82
Construct 2	^66^LPQRA^70^	0.81
Construct 2	^176^PITYS^180^	0.81
Construct 2	^259^SRARPRW^265^	0.81
Construct 2	^22^SCLPKEEQ^29^	0.80

**Table 4:** Discontinuous B-cell epitopes of the final vaccines.

Discontinuous B cell epitopes	Number of residues	Score	
A:A250, A:A251, A:Y252, A:G253, A:A254, A:P255, A:I256, A:T257, A:Y258, A:S259, A:A275, A:S278, A:K279, A:A282, A:A283, A:Y284, A:I285, A:E286, A:P287, A:L288, A:D289, A:L290, A:P291, A:Q292, A:R306, A:A307, A:A308, A:Y309, A:T310, A:P311, A:I312, A:D313, A:V356, A:G357, A:I358, A:Y359, A:L360, A:A361, A:A362, A:Y363, A:W364, A:H366, A:R367, A:A368, A:R369	45	0.87	Construct 1
A:A562, A:R563, A:A564, A:Q565, A:A566, A:P567, A:K568, A:Y571, A:K586, A:P620, A:L621, A:L622, A:K623, A:K624, A:R625, A:L626, A:K627, A:P628, A:T629, A:L630, A:R631, A:G632, A:P633, A:T634, A:P635, A:L636, A:T645, A:D646, A:N647, A:S648, A:T649, A:P650, A:P651	33	0.87	Construct 1
A:K45, A:A46, A:E47, A:I48, A:L49, A:D50, A:K51, A:S52, A:D59, A:I61, A:L62, A:K63, A:G64, A:A65, A:G66, A:S67, A:L70, A:T71, A:V72, A:V73, A:K74, A:I76, A:K77, A:D78, A:L79, A:I80, A:G81, A:L82, A:G83, A:L84, A:K85, A:E86, A:S87, A:K88, A:D89, A:V91, A:D92, A:K96, A:K100, A:G101, A:L102, A:S103, A:K104, A:E105, A:E106, A:A107, A:E108, A:S109, A:L145, A:P146, A:Q147, A:R148, A:P177, A:V178, A:S179, A:Y180	56	0.84	Construct 1
A:R580, A:V589, A:Y590, A:A591, A:T592, A:T593, A:S594, A:A597, A:Q601, A:K602, A:A614, A:I615, A:Y616, A:G617, A:T618, A:W619	16	0.83	Construct 1
A:V510, A:Y511, A:A512, A:T513, A:T514, A:S515, A:A518, A:R521, A:K523, A:K524, A:G526, A:R527, A:L528, A:V529, A:P530, A:G531, A:M532, A:T533, A:Y534, A:A535, A:I536, A:G538, A:T539, A:W540, A:R546	25	0.88	Construct 2
A:V13, A:G15, A:G16, A:R17, A:C18	5	0.87	Construct 2
A:S180, A:S199, A:K200, A:A203, A:A204, A:Y205, A:I206, A:E207, A:P208, A:L209, A:D210, A:L211, A:P212, A:Q213, A:I214, A:A215, A:A216, A:V223, A:P224, A:P225, A:L226, A:A228, A:A229, A:Y230, A:T231, A:P232, A:I233, A:D234, A:T236, A:I237, A:M238, A:A239, A:K240, A:S241, A:E242	35	0.84	Construct 2
A:G1, A:I2, A:I3, A:N4, A:T5, A:L6, A:Q7, A:K8, A:Y10, A:C11, A:R12, A:R14, A:S22, A:C23, A:L24, A:P25, A:K26, A:E27, A:E28, A:Q29, A:C41, A:K44, A:K45, A:A65, A:R97, A:P98, A:V99, A:S100, A:Y101, A:A102, A:A103, A:L120	32	0.84	Construct 2
A:A485, A:Q486, A:A487, A:P549, A:T550, A:L551, A:R552, A:P572, A:A573, A:V574, A:P575, A:K576, A:K577, A:P578, A:A579, A:Q581	16	0.83	Construct 2
A:R42, A:Y104, A:A105, A:P106, A:T107, A:G108, A:S109, A:G110, A:K111, A:S112, A:T113, A:A114, A:H124, A:K126, A:K128, A:A129, A:L136, A:S137, A:P138, A:R139	20	0.82	Construct 2

### 3.7. Molecular dynamic simulation of multi-epitope vaccines

The RMSD of the constructs Cα atoms raise up over time which highlighted a continues increasing trend up to 25 ns and next reached a plateau indicating a stable structural conformations, while the RMSD value converged within ~1 nm (Fig 3A) Likewise, the Cα-RMSF graph of the constructs during 100 ns of MD simulation was identified as stable dynamics behavior (Fig 3B). However, higher fluctuation rates were observed in the loop regions. The similar patterns of the RMSD and RMSF and low changes at various time intervals, revealed good structural stability of the vaccine constructs.

### 3.8. Molecular docking of the immune receptors (TLRs) with adjuvant and vaccines

A diverse hydrophobic and hydrophilic interactions participated in the adjuvants-TLRs binding (S3 Fig). Moreover, the molecular docking of the final vaccines after 100 ns simulations with TLR3 (PDB ID: 2A0Z) and TLR4 (PDB ID: 4G8A) was performed (S4 Fig). Next, the complex with best ClusPro score was selected and further applied for running MD simulation investigations.

### 3.9. Molecular dynamic simulation of multi-epitope vaccines-TLRs complexes

The molecular dynamics simulations of the docked complexes were performed for 100 ns. These simulations studies are capable to address several important queries about the structural stability of the final complexes. To address these queries, it is warranted to assess several main statistical factors based on MD simulation outputs (Fig 3C–3G).

The conformational stability of the vaccines and TLRs in the complexes states was investigated based on the calculation of the overall fluctuations of the final vaccines. The RMSD values were calculated as a time-dependent parameter for decoding the displacement of Cα atoms in the final vaccine constructs and TLRs during simulation times (Fig 3C and 3D). Fluctuations in the vaccine’s RMSD graph could be due to the movement of the residues to achieve a suitable and stable conformation of the vaccines in the complexes states. The RMSD fluctuations of the vaccines structures in the complexes states were lower than free states vaccines, which indicated the stability of the vaccines structures during complexes simulations (Fig 3). In addition, the RMSD graph of the TLRs structures in the complexes states were low and smooth, which indicated their structural stability in the complexes states (Fig 3).

Local structural fluctuations were estimated utilizing the RMSF factor for the vaccines and TLRs structures during the complexes simulations (Fig 3E and 3F). Loop areas exhibited highest fluctuations in the complexes states. The RMSF graph of the TLRs showed low flexibility, which clarified that TLRs had good structural stability in the complexes states (Fig 3F).

The high value of Rg (Fig 3G) indicates a decrease in protein compactness. Fluctuations in the Rg diagram indicate the movements of the vaccines domains which induce conformational changes and thereby select a suitable conformation in the complexes states. Hence, the studied complexes had a proper structural stability.

### 3.10. Estimation of binding free energy of immune receptor-vaccine complex

**Fig 4:**
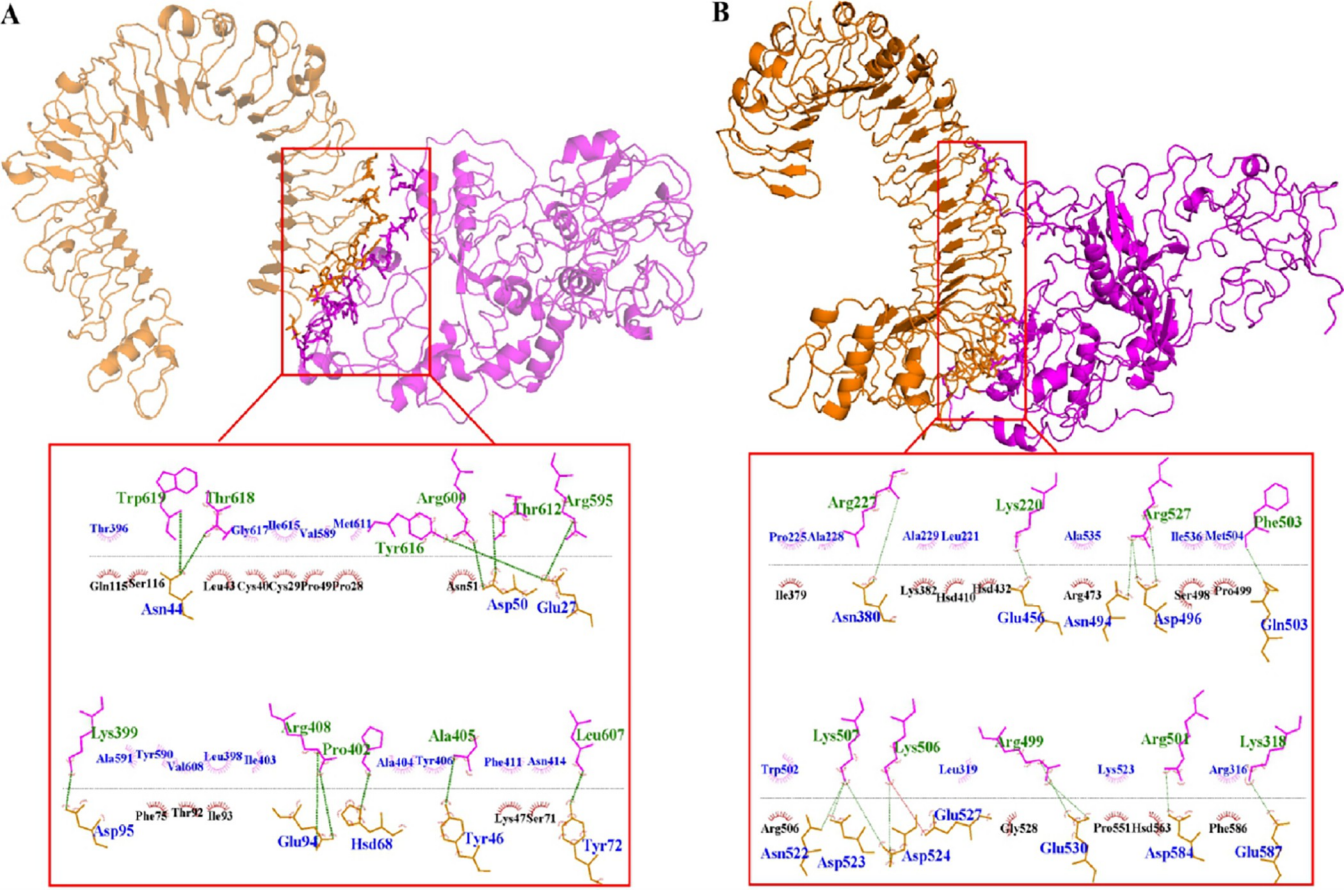
The 3D view of the final system conformations. The interface residues between two proteins TLRs (orange cartoon) and vaccines (magenta cartoon) residues (orange and magenta sticks) are labeled. Hydrogen bonds and hydrophobic contacts are presented as green dashed line and arc with spokes radiating, respectively. A and B indicate TLR4-construct 1 and TLR3-construct 2 complexes, respectively.

Both hydrophilic (ΔE_polar_) and hydrophobic energy terms (ΔE_nonpolar_) were participated in the binding of vaccines to the TLRs (Table 5). However, hydrophilic energy was as main driving force in the interactions between the immune receptors and the vaccines, compared to hydrophobic energy. Paramount residues which were participated at the interface regions have been identified (Fig 4). Accordingly, both polar and non-polar residues played key roles at the binding process, of the vaccines-TLRs complexes (Fig 4).

**Table 5:** The contribution of various energy components in the ΔG_bind_ (kJ/mol).

	Construct 1-TLR 4	Construct 2-TLR 3
ΔE_ele_^a^	-15786.76 ± 490.57	-11730.95 ± 240.61
ΔE_vdW_^b^	-460.52 ± 87.34	-247.89 ± 68.97
ΔG_PB_^c^	1476.22 ± 249.39	2323.25 ± 429.45
ΔG_SA_^d^	-64.67 ± 8.02	-45.45 ± 8.03
ΔE_non-polar_^e^	-525.19 ± 47.68	-9407.7 ± 335.03
ΔE_polar_^f^	-14310.54 ± 369.95	-293.34 ± 38.5
ΔG_bind_	-14835.73 ± 208.82	-9701.04 ± 186.76

^a^Electrostatic connection

^b^van der Waals connection

^c^Polar contribution of the solvation effect

^d^Non-polar contribution of solvation effect

^e^ ΔE_non-polar =_ ΔE_vdW +_ ΔG_SA_

^f^ ΔE_polar =_ ΔE_ele +_ ΔG_GB_

## 4. Discussion

Following the HCV protein sequences alignment, we used the IEDB MHC-II prediction tool, the NetCTL 1.2 and the BCPred servers to predict HTL (15-mer), CTL and B-cell linear epitopes, respectively (S1–S9 Tables). Those antigenic and non-allergenic epitopes were adopted and next those antigenic B and HTL epitopes were used for the construction of our multi-epitope vaccines (Table 1).

Selected epitopes containing 18 CTL, 7 HTL and 5 LBL epitopes were fused together using AAY and KK linkers, respectively (Fig 2A). Two adjuvants including 50S ribosomal protein L7/L12 (TLR4 agonist) and human β-defensin 2 (TLR3 agonist) were added separately to the N terminal domain of the final multi-epitope vaccine using an EAAAK linker to increase immunogenicity (Fig 2A), synthesizing constructs with 686 and 607 residues (S1 Fig). These designed vaccines were also potentially antigenic and non-allergenic. Their molecular weight included 76.27 kDa and 67.87 kDa, and their theoretical pI were 9.98 and 10.16 (Table 2), which indicates the basic nature of the vaccines. The instability and the aliphatic indices of the final vaccines have been represented in the Table 2, which indicates their appropriate structure and high thermo-stability. The final vaccines also deciphered a grand average of hydropathicity (GRAVY) values of -0.152 and -0.169, confirming their hydrophilic characters (Table 2). Therefore, they can interact with other proteins and are soluble in water. The predicted secondary structure of the final vaccine has been exhibited in the S1 Fig.

Galaxy server, SWISS-MODEL, ProSA-web and ERRAT servers (Fig 2) demonstrated that in the best three-dimensional structural model of the construct 1, 91.08% of the residues placed in the favored area, 7.17% in the allowed area and 1.75% in the disallowed area of Ramachandran plot (Fig 2B). In addition, the quality factor obtained from the ERRAT analysis was 76.41% and its Z-score was −3.28 (Fig 2C). Regarding the construct 2, 91.07% of the residues were in the favored area, 7.28% in the allowed area and 1.65% in the disallowed area of Ramachandran plot (Fig 2D). In addition, the quality factor and Z-score included 72.64% and −3.88, respectively (Fig 2E). A ≥90% reliability cut-off is mandatory for residues placing in the favoured region [43] which was confirmed in our study. The ERRAT score of 76.41% and 72.64% for the vaccine constructs clarified the adequate quality and validity percentage. An ERRAT score >50 indicates a suitable quality model [44]. All of these indicators confirm the good quality of the 3D structures of the final vaccines (Fig 3F and 3G).

We observed four discontinuous B-cell epitopes with a score of 0.83 to 0.87, and 12 linear B-cell epitopes with a score of 0.83 to 0.93, selected as the final epitopes in 50S ribosomal protein L7/L12-multi-epitope vaccine (S2 Fig, Tables 3 and 4). In addition, six discontinuous B-cell epitopes with score of 0.82 to 0.88, and 14 linear B-cell epitopes with score of 0.83 to 0.91 were selected as the final epitopes in human β-defensin 2-multi-epitope vaccine (S2 Fig, Tables 3 and 4). For both multi-epitope vaccines, the PI values of 0.87 and 0.88 demonstrated that 87% and 88% of residues located in the predicted ellipsoid area of the epitopes, having the highest solvent accessibility. Following the MD simulations for 100 ns, in the RMSD of the constructs, Cα atoms raise up over time which highlighted a continues increasing trend up to 25 ns and then reaching a plateau which indicated a stable structural conformations, while the RMSD value converged within ~1 nm (Fig 3A). Likewise, the Cα-RMSF graph of the constructs deciphered stable dynamics behavior (Fig 3B). However, higher fluctuation rates were observed in the loop regions. Similar patterns of the RMSD and RMSF and low changes at various time intervals, revealed good structural stability of the vaccine constructs. These final constructs were used as the ligands for docking to the TLRs. The molecular docking demonstrated the interaction of the TLRs with the adjuvants. Accordingly, a diverse hydrophobic and hydrophilic interactions participated in the adjuvants-TLRs binding (S3 Fig).

Moreover, the molecular docking of the final vaccines after 100 ns simulations with TLR3 (PDB ID: 2A0Z) and TLR4 (PDB ID: 4G8A) was performed (S4 Fig). The simulations studies were capable of addressing main queries regarding the structural stability of the final complexes depicted as outputs (Fig 3C–3G).

The conformational stability of the vaccines and TLRs in the complexes states was investigated based on the calculation of the overall fluctuations of the final vaccines. The RMSD values were calculated as a time-dependent parameter for decoding the displacement of Cα atoms in the final vaccine constructs and TLRs during simulation times (Fig 3C and 3D). Fluctuations in the vaccine’s RMSD graph could be due to the movement of the residues to form a suitable and stable conformation of the vaccines in the complexes states, being lower than free states vaccines, which indicated the stability of the vaccines structures during complexes simulations (Fig 3). In addition, the RMSD graph of the TLRs structures in the complexes states were low and smooth, which indicated their structural stability in the complexes states (Fig 3).

Local structural fluctuations estimation utilizing the RMSF factor (Fig 3E and 3F) revealed highest fluctuations of loop areas in the complexes states. These localized fluctuations in vaccines residues can facilitate vaccine epitopes’ exposure to TLRs and make sufficient space for side chains to adopt suitable conformation for binding to TLRs. The RMSF graph of the TLRs showed low flexibility, clarifying their sufficient structural stability in the complexes states (Fig 3F).

The high value of Rg indicates a decrease in protein compactness. Fluctuations in the Rg diagram indicate the movements of the vaccines domains which induce conformational changes and thereby select a suitable conformation in the complexes states. According to the pattern of changes in these factors, our vaccines structures system displayed high stability during complexes simulations (Fig 3G). Accordingly, the multi- epitope vaccines-TLRs constructs achieved a suitable and stable conformational complexes during the simulations times, highlighting their proper structural stability.

The binding free energy profile of our vaccine candidates (Table 5) [45] revealed that both hydrophilic (ΔE_polar_) and hydrophobic energy terms (ΔE_nonpolar_) participated in the binding of vaccines to the TLRs. However, hydrophilic energy was the main driving force in the interactions, compared to hydrophobic energy (Fig 4). Accordingly, both polar and non-polar residues played key roles in the binding process, of the vaccines-TLRs complexes (Fig 4).

Noticeably, the output of the TLRs-vaccines complexes outlined the high structural stability of molecular species docked together during the simulation. Hydrophilic residues play a vital role in the interactions between the vaccines and the TLRs, which in turn determine high stability of the complex structure. Indeed, these binary interactions cause the efficient adhesion of molecular species together. Accordingly, the vaccine candidate constructs had the ability to stimulate the immune system’s responses efficiently though further studies are warranted to verify these findings in vitro and in vivo. In previous studies multi-epitope vaccine constructs have been used against *Leishmania donovani* stimulating CD8+ T cells and INFγ (19 antigenic proteins, 49 epitopes binding to 40 different MHC class I supertypes) [46], provoking CD8+ T cells (LeIF and TSA) [47] and induction of CD4+, CD8+, IFN-γ and IL-10 [48]. Major limitations of our survey included lack of experimental assessment of the multi-epitope vaccine in vitro, in vivo or in clinical trial. A study using multi-epitope DNA- and peptide-based vaccines provoked CD4+ and CD8+ responses against the HCV and were evaluated in BALB/c mice model [49].

## 5. Conclusion

The efficient anti-HCV treatment approaches are limited due to various side effects, recurrency and antiviral resistance development. Thereby, it is essential to seek other strategies like vaccine design. Currently, it is possible to design recombinant and multi-epitope vaccines more rapid and cost effective using a variety of computational methods. Accordingly, we attempted to design multi-epitope vaccine candidates based on the conserved areas of the HCV structural and non-structural protein sequences using the immunoinformatics tools. According to our findings, the vaccines constructs (including CTL, HTL and B cell epitopes plus two adjuvants or TLR3 and TLR4 agonists) were efficient in terms of antigenicity, non-toxicity, solubility, immunogenicity, non-allergenicity and stability. The 3D structures of the multi-epitope vaccine candidates’ models were highly stable and soluble. Additionally, their interactions with the TLR3 and TLR4 resulted in efficient vaccine candidates with acceptable traits in silico. Our computational findings were promising considering the candidate multi-epitope vaccines potential in controlling the HCV infection.

## Supporting information

S1 Table Linear B cell (LBL) epitopes of the P7 protein. (DOCX)

S2 Table Linear B cell (LBL) epitopes of the NS3 protein. (DOCX)

S3 Table Linear B cell (LBL) epitopes of the NS5B protein. (DOCX)

S4 Table Helper T Lymphocyte (HTL) epitopes of the NS5B protein. (DOCX)

S5 Table Helper T Lymphocyte (HTL) epitopes of the NS3 protein. (DOCX)

S6 Table Helper T Lymphocyte (HTL) epitopes of the P7 protein. (DOCX)

S7 Table Cytotoxic T Lymphocyte (CTL) epitopes of the P7protein. (DOCX)

S8 Table Cytotoxic T Lymphocyte (CTL) epitopes of the NS3 protein. (DOCX)

S9 Table Cytotoxic T Lymphocyte (CTL) epitopes of the NS5B protein. (DOCX)

S1 Fig Prediction of the secondary structure of the construct 1 (A) and construct 2 (B) vaccines. The predicted results showed that among 686 amino acids in the construct 1, 268 (39.07%), 135 (19.68%), 66 (9.62%) and 217 (31.63%) amino acids are involved in α-helix, extended strand, beta turn, and random coil, respectively. Our predicted outputs revealed that among 607 amino acids in the construct 2, 205 (33.77%), 132 (21.75%), 63 (10.38%) and 207 (34.10%) amino acids are involved in α-helix, extended strand, beta turn, and random coil, respectively.
(DOCX)

S2 Fig Linear (A and C) and Discontinuous (B and D) B-cell epitopes of the construct 1 (A and B) and construct 2 (C and D) vaccines (colored spheres).
(DOCX)

S3 Fig The 3D view of the molecular docking conformations. The interface residues between two proteins TLRs (orange cartoon) and adjuvants (magenta cartoon) residues (orange and magenta sticks) are labeled. Hydrogen bonds and hydrophobic contacts are presented as green dashed line and arc with spokes radiating, respectively. A and B indicate TLR4-50S ribosomal protein L7/L12 and TLR3-human β-defensin 2 complexes, respectively.
(DOCX)

S4 Fig A and B indicate TLR4-construct 1 and TLR3-construct 2 complexes, respectively. TLRs and costructs are depicted as brown and blue cartoons respectively.
(DOCX)

S1 Data Multiple alignment of the NS3 protein sequences in HCV strains. (PDF)

S2 Data Multiple alignment of the P7 protein sequences in HCV strains. (PDF)

S3 Data Multiple alignment of the NS5B protein sequences in HCV strains. (PDF)
