# Supplementary material for: Design of a novel multi-epitope vaccine candidate against hepatitis C virus using structural and nonstructural proteins: An immunoinformatics approach
Source: PLoS One. 2022 Aug 30;17(8):e0272582. doi: 10.1371/journal.pone.0272582 (PMC9426923; doi:10.1371/journal.pone.0272582)
Supplement: S1 Table — (DOCX) [file pone.0272582.s001.docx]

**Table S1:** Linear B cell (LBL) epitopes of the P7 protein

| Position | Epitope | Antigenicity | Score | Allergenicity |
| --- | --- | --- | --- | --- |
| 32 | IKGRLVPGMTYAIYGTWPLL | 0.6257 | 1 | NON-ALLERGEN |
