# Supplementary material for: Design of a novel multi-epitope vaccine candidate against hepatitis C virus using structural and nonstructural proteins: An immunoinformatics approach
Source: PLoS One. 2022 Aug 30;17(8):e0272582. doi: 10.1371/journal.pone.0272582 (PMC9426923; doi:10.1371/journal.pone.0272582)
Supplement: S2 Table — (DOCX) [file pone.0272582.s002.docx]

**Table S2:** Linear B cell (LBL) epitopes of the NS3 protein

| Position | Epitope | Antigenicity | Score | Allergenicity |
| --- | --- | --- | --- | --- |
| 181 | SPTFTDNSTPPAVP | 0.6346 | 1 | NON-ALLERGEN |
| 133 | SYLKGSSGGPLLCP | 0.1020 | 1 | ALLERGEN |
| 321 | ATATPPGSVTVPHP | 0.3040 | 1 | ALLERGEN |
| 570 | RASAPPPSWDQMWK | 0.1760 | 1 | NON-ALLERGEN |
| 275 | ADGGCSGGAYDIII | 0.0527 | 1 | NON-ALLERGEN |
| 587 | RLKPTLRGPTPLLY | 0.7366 | 1 | NON-ALLERGEN |
| 113 | VIPVRRRGDTRGSL | 0.7219 | 1 | ALLERGEN |
| 86 | PAPQGTRSLTPCTC | 0.4383 | 1 | NON-ALLERGEN |
| 25 | DKNQVEGEVQIVST | 0.5930 | 1 | ALLERGEN |
| 474 | RFVTPGERPSGMFD | 0.2951 | 0.997 | NON-ALLERGEN |
| 60 | GAKSLAGPKGPILQ | -0.109 | 0.996 | NON-ALLERGEN |
| 249 | DPNIRTGVRTITTG | 0.8909 | 0.996 | ALLERGEN |
| 506 | PAETSVRLRAYMNT | 0.4798 | 0.994 | NON-ALLERGEN |
