# Supplementary material for: Design of a novel multi-epitope vaccine candidate against hepatitis C virus using structural and nonstructural proteins: An immunoinformatics approach
Source: PLoS One. 2022 Aug 30;17(8):e0272582. doi: 10.1371/journal.pone.0272582 (PMC9426923; doi:10.1371/journal.pone.0272582)
Supplement: S3 Table — (DOCX) [file pone.0272582.s003.docx]

**Table S3:** Linear B cell (LBL) epitopes of the NS5B protein

| Position | Epitope | Antigenicity | Score | Allergenicity |
| --- | --- | --- | --- | --- |
| 346 | YSAPPGDPPQPEYD | 0.0443 | 1 | NON-ALLERGEN |
| 433 | LLAQEQLEKALDCQ | 0.2217 | 1 | ALLERGEN |
| 124 | KDLLEDTETPIDTT | 0.1041 | 1 | NON-ALLERGEN |
| 399 | TARHTPVNSWLGNI | -0.2071 | 0.999 | ALLERGEN |
| 141 | KSEVFCVQPEKGGR | 1.7494 | 0.997 | NON-ALLERGEN |
| 548 | SSWFVAGYSGGDIY | 0.1244 | 0.997 | NON-ALLERGEN |
| 282 | SGVLTTSCGNTLTC | -0.0149 | 0.995 | ALLERGEN |
| 83 | LSVEEACKLTPPHS | 0.4633 | 0.994 | NON-ALLERGEN |
| 475 | HSYSPGEINRVASC | 0.3119 | 0.989 | ALLERGEN |
| 495 | PPLRVWRHRARSVR | -0.3279 | 0.988 | NON-ALLERGEN |
