# Supplementary material for: Design of a novel multi-epitope vaccine candidate against hepatitis C virus using structural and nonstructural proteins: An immunoinformatics approach
Source: PLoS One. 2022 Aug 30;17(8):e0272582. doi: 10.1371/journal.pone.0272582 (PMC9426923; doi:10.1371/journal.pone.0272582)
Supplement: S5 Table — (DOCX) [file pone.0272582.s005.docx]

**Table S5:** Helper T Lymphocyte (HTL) epitopes of the NS3 protein

| Antigen | NS3-CD4+ T-cell epitopes- MHC  class II binding | IL4pred | IL10pred | IFNepitope |
| --- | --- | --- | --- | --- |
| 0.4335  0.9420  0.9169  0.5568  1.0899  0.9841  1.0074  1.0061  0.7283  0.6665  1.2638  0.6314  0.9306  0.7188  1.0040  0.4247  1.3230 1.2627  1.0486 1.1670  0.8489  1.0290  0.5868 | ^221^QGYKVLVLNPSVAAT^235^  ^556^NFPYLVAYQATVCAR^570^  ^558^PYLVAYQATVCARAQ^572^  ^555^DNFPYLVAYQATVCA^569^  ^228^LNPSVAATLGFGAYM^242^  ^229^NPSVAATLGFGAYMS^243^  ^499^CAWYELTPAETTVRL^513^  ^230^PSVAATLGFGAYMSK^244^  ^387^NAVAYYRGLDVSVIP^401^  ^535^VFTGLTHIDAHFLSQ^549^  ^165^KAVDFIPVESMETTM^179^  ^534^SVFTGLTHIDAHFLS^548^  ^498^GCAWYELTPAETTVR^512^  ^386^INAVAYYRGLDVSVI^400^  ^231^SVAATLGFGAYMSKA^245^  ^554^GDNFPYLVAYQATVC^568^  ^164^AKAVDFIPVESMETT^178^  ^500^AWYELTPAETTVRLR^514^  ^166^AVDFIPVESMETTMR^180^  ^104^LYLVTRHADVIPVRR^118^  ^536^FTGLTHIDAHFLSQT^550^  ^163^VAKAVDFIPVESMET^177^  ^129^PRPVSYLKGSSGGPL^143^ | Non IL4 inducer  IL4 inducer  IL4 inducer  IL4 inducer  Non IL4 inducer  Non IL4 inducer  IL4 inducer  Non IL4 inducer  IL4 inducer  IL4 inducer  IL4 inducer  IL4 inducer  IL4 inducer  IL4 inducer  Non IL4 inducer  IL4 inducer  IL4 inducer  IL4 inducer  IL4 inducer  Non IL4 inducer  IL4 inducer  IL4 inducer  Non IL4 inducer | IL10 non-inducer  IL10 inducer  IL10 inducer  IL10 inducer  IL10 non-inducer  IL10 non-inducer  IL10 inducer  IL10 non-inducer  IL10 non-inducer  IL10 inducer  IL10 inducer  IL10 inducer  IL10 non-inducer  IL10 inducer  IL10 non-inducer  IL10 inducer  IL10 inducer  IL10 inducer  IL10 inducer  IL10 inducer  IL10 inducer  IL10 inducer  IL10 inducer | POSITIVE  POSITIVE  POSITIVE  POSITIVE  NEGATIVE  NEGATIVE  NEGATIVE  NEGATIVE  POSITIVE  POSITIVE  NEGATIVE  POSITIVE  NEGATIVE  POSITIVE  NEGATIVE  POSITIVE  NEGATIVE  POSITIVE  NEGATIVE  NEGATIVE  POSITIVE  NEGATIVE  POSITIVE |
| 0.7342  0.6030  1.0188  0.7390  0.7390  0.8213  0.5972  0.8099  0.8465  0.5781  1.0027  0.4884  0.3073  0.6590  0.4592  0.7044  0.5532  0.6757  1.2627 | ^616^TKFIMACMSADLEVV^630^  ^617^TKFIMACMSADLEVVT^631^  ^232^VAATLGFGAYMSKAH^246^  ^230^PSVAATLGFGAYMSK^244^  ^233^AATLGFGAYMSKAHG^247^  ^378^AAKLSGLGINAVAYY^392^  ^209^GKSTKVPAAYAAQGY^223^  ^299^TILGIGTVLDQAETA^313^  ^380^KLSGLGINAVAYYRG^394^  ^210^KSTKVPAAYAAQGYK^224^  ^377^LAAKLSGLGINAVAY^391^  ^214^VPAAYAAQGYKVLVL^228^  ^35^VVSTATQSFLATCVN^49^  ^560^LVAYQATVCARAQAP^574^  ^312^TAGARLVVLATATPP^326^  ^208^SGKSTKVPAAYAAQG^222^  ^554^GDNFPYLVAYQATVCARAQAP^574^  ^534^SVFTGLTHIDAHFLSQT^550^  ^500^ AWYELTPAETTVRLR^514^ | IL4 inducer  IL4 inducer  Non IL4 inducer  Non IL4 inducer  Non IL4 inducer  Non IL4 inducer  IL4 inducer  Non IL4 inducer  Non IL4 inducer  IL4 inducer  Non IL4 inducer  Non IL4 inducer  Non IL4 inducer  IL4 inducer  Non IL4 inducer  IL4 inducer  IL4 inducer  IL4 inducer  IL4 inducer | IL10 non-inducer  IL10 non-inducer  IL10 non-inducer  IL10 non-inducer  IL10 non-inducer  IL10 non-inducer  IL10 non-inducer  IL10 inducer  IL10 non-inducer  IL10 non-inducer  IL10 non-inducer  IL10 non-inducer  IL10 inducer  IL10 inducer  IL10 non-inducer  IL10 non-inducer  IL10 inducer  IL10 inducer  IL10 inducer | NEGATIVE  NEGATIVE  NEGATIVE  NEGATIVE  NEGATIVE  NEGATIVE  POSITIVE  NEGATIVE  NEGATIVE  POSITIVE  NEGATIVE  POSITIVE  NEGATIVE  POSITIVE  NEGATIVE  NEGATIVE  POSITIVE  POSITIVE  POSITIVE |
