# Supplementary material for: Design of a novel multi-epitope vaccine candidate against hepatitis C virus using structural and nonstructural proteins: An immunoinformatics approach
Source: PLoS One. 2022 Aug 30;17(8):e0272582. doi: 10.1371/journal.pone.0272582 (PMC9426923; doi:10.1371/journal.pone.0272582)
Supplement: S6 Table — (DOCX) [file pone.0272582.s006.docx]

**Table S6:** Helper T Lymphocyte (HTL) epitopes of the P7 protein

| Antigen | P7-CD4+ T-cell epitopes- MHC  class II binding | IL4pred | IL10pred | IFNepitope |
| --- | --- | --- | --- | --- |
| 0.6008  0.4511  0.6850  0.58  0.6276  0.5123  0.6458  0.5278  0.4868  0.8335  0.8514  0.8583  0.7734 | ^39^GMTYAIYGTWPLLLL^53^  ^38^PGMTYAIYGTWPLLL^52^  ^42^YAIYGTWPLLLLLLA^56^  ^33^KGRLVPGMTYAIYGT^47^  ^11^ASLAGTHGLLSFLVF^25^  ^32^IKGRLVPGMTYAIYG^46^  ^30^WYIKGRLVPGMTYAI^44^  ^36^LVPGMTYAIYGTWPLLLL^53^  ^12^SLAGTHGLLSFLVFF^26^  ^26^FCAAWYIKGRLVPGM^40^  ^27^CAAWYIKGRLVPGMT^41^  ^25^FFCAAWYIKGRLVPG^39^  ^29^AWYIKGRLVPGMTYA^43^ | IL4 inducer  IL4 inducer  IL4 inducer  Non IL4 inducer  Non IL4 inducer  Non IL4 inducer  Non IL4 inducer  IL4 inducer  Non IL4 inducer  Non IL4 inducer  Non IL4 inducer  Non IL4 inducer  Non IL4 inducer | IL10 inducer  IL10 inducer  IL10 non-inducer  IL10 inducer  IL10 inducer  IL10 inducer  IL10 inducer  IL10 inducer  IL10 inducer  IL10 inducer  IL10 inducer  IL10 inducer  IL10 inducer | NEGATIVE  NEGATIVE  NEGATIVE  NEGATIVE  NEGATIVE  NEGATIVE  NEGATIVE  NEGATIVE  NEGATIVE  NEGATIVE  NEGATIVE  NEGATIVE  NEGATIVE |
