# Supplementary material for: Design of a novel multi-epitope vaccine candidate against hepatitis C virus using structural and nonstructural proteins: An immunoinformatics approach
Source: PLoS One. 2022 Aug 30;17(8):e0272582. doi: 10.1371/journal.pone.0272582 (PMC9426923; doi:10.1371/journal.pone.0272582)
Supplement: S7 Table — (DOCX) [file pone.0272582.s007.docx]

**Table S7:** Cytotoxic T Lymphocyte (CTL) epitopes of the P7protein

| MHC supertype A1 | ^54^LLALPQRAY |
| --- | --- |
| MHC supertype A2 | ^42^YAIYGTWPL ^35^RLVPGMTYA  ^46^GTWPLLLLL ^43^AIYGTWPLL  ^53^LLLALPQRA ^18^GLLSFLVFF |
| MHC supertype A3 | ^23^LVFFCAAWY ^52^LLLLALPQR  ^54^LLALPQRAY |
| MHC supertype A24 | ^44^IYGTWPLLL ^47^TWPLLLLLL  ^24^VFFCAAWYI |
| MHC supertype A26 | ^42^YAIYGTWPL  ^43^AIYGTWPLL  ^23^LVFFCAAWY |
| MHC supertype B7 | ^42^YAIYGTWPL ^5^LVVLNAASL  ^28^AAWYIKGRL |
| MHC supertype B8 | ^49^PLLLLLLAL ^42^YAIYGTWPL  ^17^HGLLSFLVF ^31^YIKGRLVPG |
| MHC supertype B27 | ^34^GRLVPGMTY |
| MHC supertype B39 | ^42^YAIYGTWPL ^44^IYGTWPLLL |
| MHC supertype B44 |  |
| MHC supertype B58 | ^40^MTYAIYGTW ^22^FLVFFCAAW  ^42^YAIYGTWPL |
| MHC supertype B62 | ^54^LLALPQRAY ^23^LVFFCAAWY  ^42^YAIYGTWPL ^22^FLVFFCAAW  ^5^LVVLNAASL |
