# Supplementary material for: Design of a novel multi-epitope vaccine candidate against hepatitis C virus using structural and nonstructural proteins: An immunoinformatics approach
Source: PLoS One. 2022 Aug 30;17(8):e0272582. doi: 10.1371/journal.pone.0272582 (PMC9426923; doi:10.1371/journal.pone.0272582)
Supplement: S8 Table — (DOCX) [file pone.0272582.s008.docx]

**Table S8:** Cytotoxic T Lymphocyte (CTL) epitopes of the NS3 protein.

| MHC supertype A1 | ^126^LLSPRPVSY ^494^CYDAGCAWY ^233^AATLGFGAY ^262^GAPITYSTY ^215^PAAYAAQGY ^383^GLGINAVAY  ^508^ETTVRLRAY |
| --- | --- |
| MHC supertype A2 | ^438^FSLDPTFTI ^622^CMSADLEVV  ^47^CVNGVCWTV |
| MHC supertype A3 | ^364^HLIFCHSKK ^365^LIFCHSKKK  ^126^LLSPRPVSY |
| MHC supertype A24 | ^217^AYAAQGYKV ^432^VTQTVDFSL |
| MHC supertype A26 | ^508^ETTVRLRAY ^384^LGINAVAYY  ^342^SNTGEIPFY |
| MHC supertype B7 | ^505^TPAETTVRL ^204^APTGSGKST  ^218^YAAQGYKVL ^406^VVIVATDAL  ^170^IPVESMETT ^86^HAPPGARSL |
| MHC supertype B8 | ^218^YAAQGYKVL |
| MHC supertype B27 | ^218^YAAQGYKVL ^505^TPAETTVRL  ^438^FSLDPTFTI |
| MHC supertype B39 |  |
| MHC supertype B44 |  |
| MHC supertype B58 | ^570^RAQAPPPSW ^126^LLSPRPVSY  ^383^LGINAVAYY ^230^PSVAATLGF |
| MHC supertype B62 | ^126^LLSPRPVSY ^414^LMTGYTGDF  ^383^LGINAVAYY ^605^VQNEITLTH  ^465^TGRGRGGIY ^384^LGINAVAYY  ^233^AATLGFGAY ^218^YAAQGYKVL  ^262^GAPITYSTY ^406^VVIVATDAL  ^508^ETTVRLRAY ^508^ETTVRLRAYL |
