# Supplementary material for: Design of a novel multi-epitope vaccine candidate against hepatitis C virus using structural and nonstructural proteins: An immunoinformatics approach
Source: PLoS One. 2022 Aug 30;17(8):e0272582. doi: 10.1371/journal.pone.0272582 (PMC9426923; doi:10.1371/journal.pone.0272582)
Supplement: S9 Table — (DOCX) [file pone.0272582.s009.docx]

Table S9: Cytotoxic T Lymphocyte (CTL) epitopes of the NS5B protein

|  | Epitopes | Antigenicity>0.4 | Allergenicity |
| --- | --- | --- | --- |
| MHC supertype A1 | ^469^LSAFSLHSY  ^56^RLQVLDDHY  ^30^LLRHHNMVY  ^375^DASGKRVYY | 0.5033 ( Probable ANTIGEN )  0.7380 ( Probable ANTIGEN )  0.4624 ( Probable ANTIGEN )  0.4112 ( Probable ANTIGEN ) | NON-ALLERGEN  NON-ALLERGEN  NON-ALLERGEN  NON-ALLERGEN |
| MHC supertype A2 | ^524^YLFNWAVRT  ^577^LLLSVGVGI  ^29^SLLRHHNMV  ^575^CLLLLSVGV  ^572^FMWCLLLLS  ^59^VLDDHYRDV | 0.6932 ( Probable ANTIGEN )  1.2610 ( Probable ANTIGEN )  0.4241 ( Probable ANTIGEN )  1.0094 ( Probable ANTIGEN )  0.8177 ( Probable ANTIGEN )  0.6331 ( Probable ANTIGEN ) | NON-ALLERGEN  NON-ALLERGEN  NON-ALLERGEN  NON-ALLERGEN  NON-ALLERGEN  NON-ALLERGEN |
| MHC supertype A3 | ^30^LLRHHNMVY  ^43^RSASLRQKK  ^469^LSAFSLHSY  ^562^HSLSRARPR | 0.4624 ( Probable ANTIGEN )  1.0880 ( Probable ANTIGEN )  0.5033 ( Probable ANTIGEN )  0.6091 ( Probable ANTIGEN ) | NON-ALLERGEN  NON-ALLERGEN  NON-ALLERGEN  NON-ALLERGEN |
| MHC supertype A24 | ^3^SYTWTGALI  ^571^WFMWCLLLL  ^570^RWFMWCLLL  ^523^KYLFNWAVR  ^216^GFSYDTRCF | 0.4709 ( Probable ANTIGEN )  1.2641 ( Probable ANTIGEN )  1.6596 ( Probable ANTIGEN )  0.4353 ( Probable ANTIGEN )  1.1777 ( Probable ANTIGEN ) | NON-ALLERGEN  NON-ALLERGEN  NON-ALLERGEN  NON-ALLERGEN  NON-ALLERGEN |
| MHC supertype A26 | ^469^LSAFSLHSY | 0.5033 ( Probable ANTIGEN ) | NON-ALLERGEN |
| MHC supertype B7 | ^539^IPAASQLDL  ^539^ATTSRSASL  ^132^TPIDTTIMA  ^46^SLRQKKVTF | 0.7892 ( Probable ANTIGEN )  0.9402 ( Probable ANTIGEN )  0.4542 ( Probable ANTIGEN )  1.7893 ( Probable ANTIGEN ) | NON-ALLERGEN  NON-ALLERGEN  NON-ALLERGEN  NON-ALLERGEN |
| MHC supertype B8 | ^46^SLRQKKVTF  ^531^RTKLKLTPI  ^207^AWKSKKSPM  ^2^MSYTWTGAL  ^30^LLRHHNMVY  ^528^WAVRTKLKL | 1.7893 ( Probable ANTIGEN )  2.2509 ( Probable ANTIGEN )  1.2625 ( Probable ANTIGEN )  0.6599 ( Probable ANTIGEN )  0.4624 ( Probable ANTIGEN )  1.5662 ( Probable ANTIGEN ) | NON-ALLERGEN  NON-ALLERGEN  NON-ALLERGEN  NON-ALLERGEN  NON-ALLERGEN  NON-ALLERGEN |
| MHC supertype B27 | ^500^WRHRARSVR  ^516^GRAATCGKY  ^509^ARLLSQGGR  ^42^SRSASLRQK  ^490^RKLGVPPLR  ^489^LRKLGVPPL  ^199^QRVEFLVNA  ^565^SRARPRWFM  ^211^KKSPMGFSY | 0.7950 ( Probable ANTIGEN )  0.5689 ( Probable ANTIGEN )  0.6435 ( Probable ANTIGEN )  0.8380 ( Probable ANTIGEN )  1.7694 ( Probable ANTIGEN )  0.8730 ( Probable ANTIGEN )  0.8495 ( Probable ANTIGEN )  1.0847 ( Probable ANTIGEN )  1.7388 ( Probable ANTIGEN ) | NON-ALLERGEN  NON-ALLERGEN  NON-ALLERGEN  NON-ALLERGEN  NON-ALLERGEN  NON-ALLERGEN  NON-ALLERGEN  NON-ALLERGEN  NON-ALLERGEN |
| MHC supertype B39 | ^103^YGAKDVRNL  ^2^MSYTWTGAL  ^489^LRKLGVPPL | 1.4463 ( Probable ANTIGEN )  0.6599 ( Probable ANTIGEN )  0.8730 ( Probable ANTIGEN | NON-ALLERGEN  NON-ALLERGEN  NON-ALLERGEN |
| MHC supertype B44 | ^330^QEDAASLRV  ^454^IEPLDLPQI | 0.5003 ( Probable ANTIGEN )  1.2303 ( Probable ANTIGEN ) | NON-ALLERGEN  NON-ALLERGEN |
| MHC supertype B58 | ^563^SLSRARPRW  ^469^LSAFSLHSY  ^209^KSKKSPMGF  ^211^KKSPMGFSY  ^579^LSVGVGIYL  ^389^TTPLARAAW | 1.1393 ( Probable ANTIGEN )  0.5033 ( Probable ANTIGEN )  1.7146 ( Probable ANTIGEN )  1.7388 ( Probable ANTIGEN )  1.2218 ( Probable ANTIGEN )  0.5487 ( Probable ANTIGEN ) | NON-ALLERGEN  NON-ALLERGEN  NON-ALLERGEN  NON-ALLERGEN  NON-ALLERGEN  NON-ALLERGEN |
| MHC supertype B62 | ^30^LLRHHNMVY  ^469^LSAFSLHSY  ^56^RLQVLDDHY  ^46^SLRQKKVTF  ^2^MSYTWTGAL  ^209^KSKKSPMGF  ^211^KKSPMGFSY  ^564^LSRARPRWF  ^52^VTFDRLQVL | 0.4624 ( Probable ANTIGEN )  0.5033 ( Probable ANTIGEN )  0.7380 ( Probable ANTIGEN )  1.7893 ( Probable ANTIGEN )  0.6599 ( Probable ANTIGEN )  1.7146 ( Probable ANTIGEN )  1.7388 ( Probable ANTIGEN )  0.9609 ( Probable ANTIGEN )  0.5322 ( Probable ANTIGEN ) | NON-ALLERGEN  NON-ALLERGEN  NON-ALLERGEN  NON-ALLERGEN  NON-ALLERGEN  NON-ALLERGEN  NON-ALLERGEN  NON-ALLERGEN  NON-ALLERGEN |
