# Supplementary material for: Design of a novel multi-epitope vaccine candidate against hepatitis C virus using structural and nonstructural proteins: An immunoinformatics approach
Source: PLoS One. 2022 Aug 30;17(8):e0272582. doi: 10.1371/journal.pone.0272582 (PMC9426923; doi:10.1371/journal.pone.0272582)
Supplement: S1 Fig — Prediction of the secondary structure of the construct 1 (A) and construct 2 (B) vaccines. The predicted results showed that among 686 amino acids in the construct 1, 268 (39.07%), 135 (19.68%), 66 (9.62%) and 217 (31.63%) amino acids are involved in α-helix, extended strand, beta turn, and random coil, respectively. Our predicted outputs revealed that among 607 amino acids in the construct 2, 205 (33.77%), 132 (21.75%), 63 (10.38%) and 207 (34.10%) amino acids are involved in α-helix, extended strand, beta turn, and random coil, respectively. (DOCX) [file pone.0272582.s010.docx]

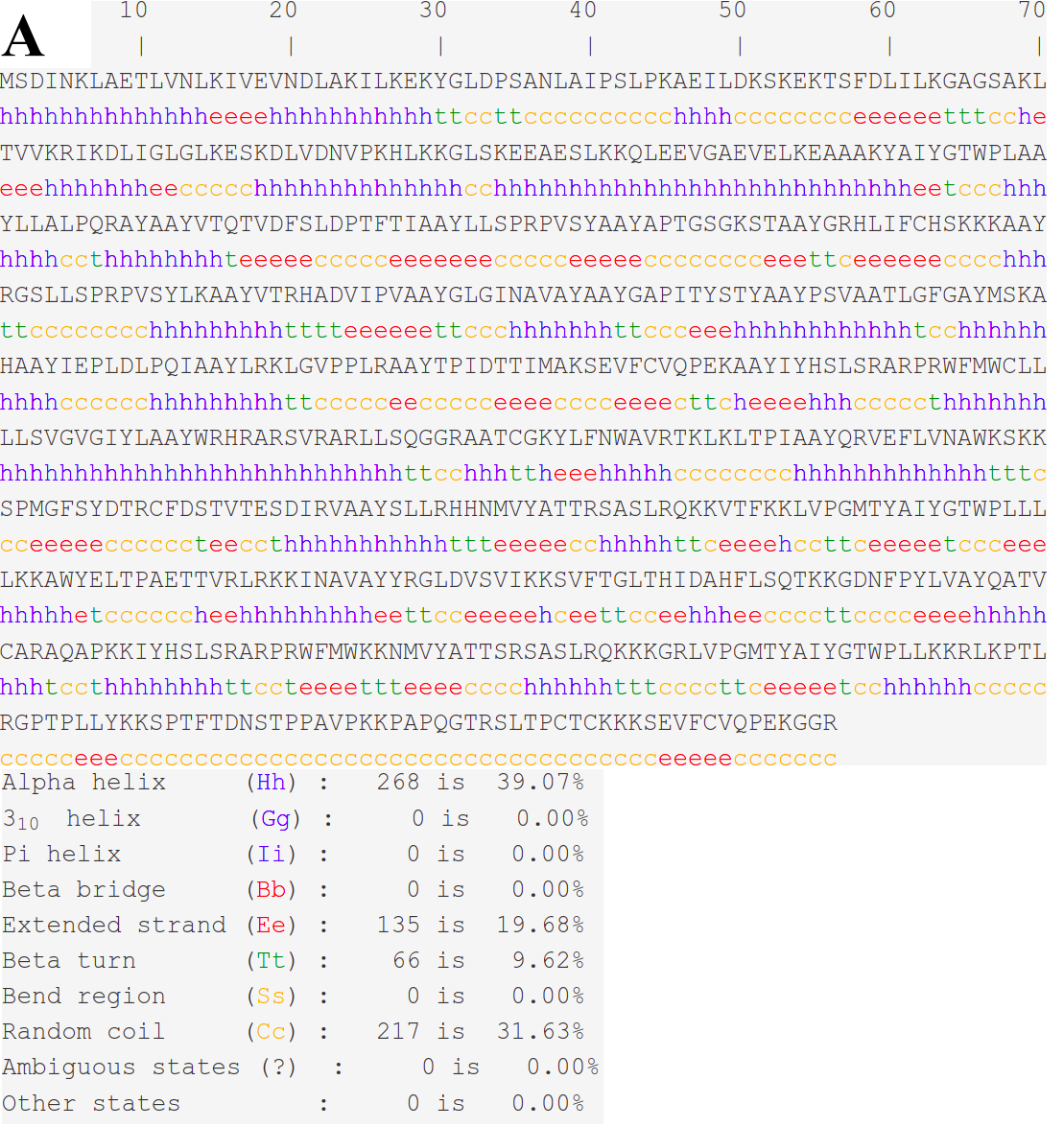

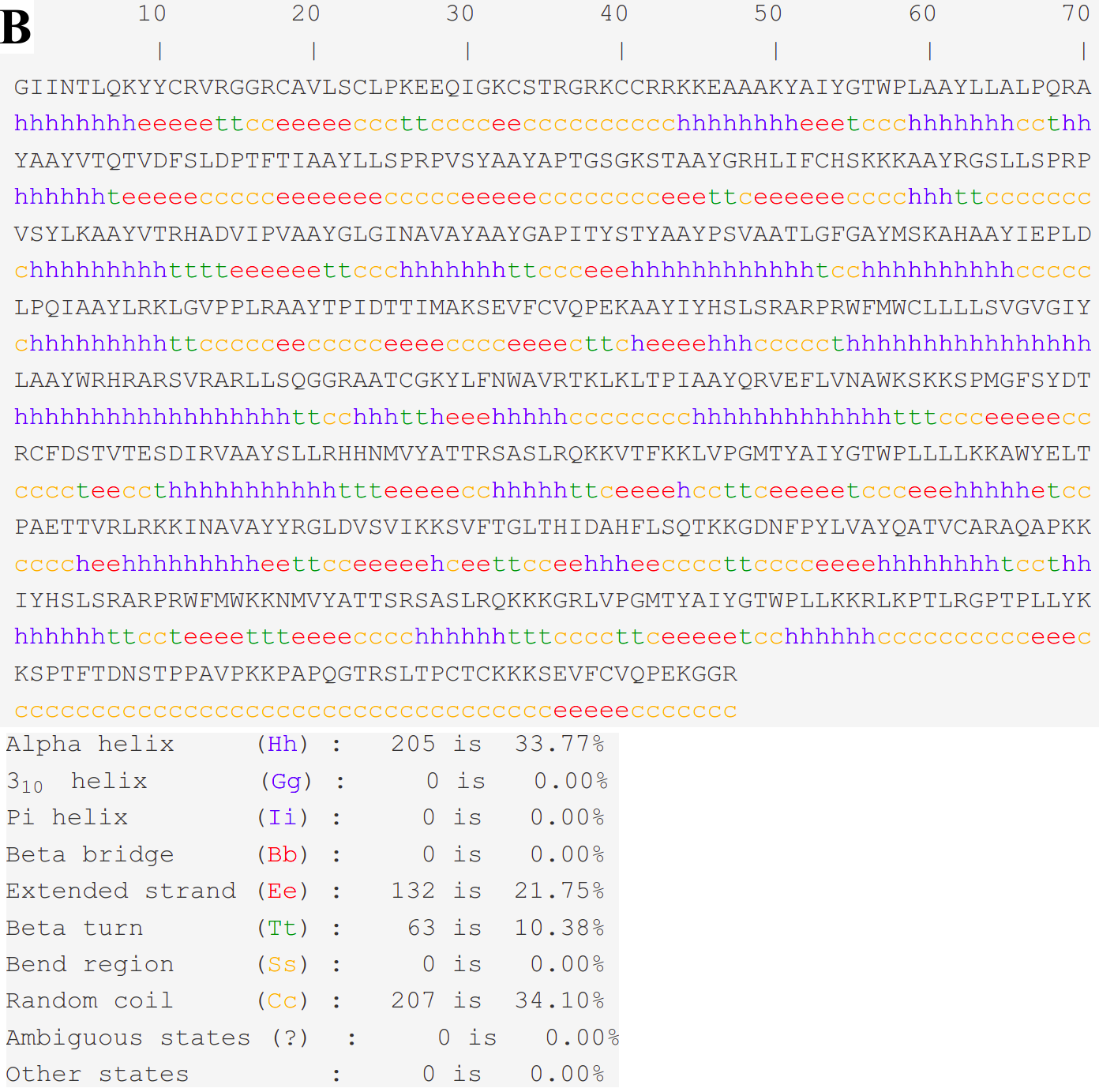


**Figure S1:** Prediction of the secondary structure of the construct 1 (A) and construct 2 (B) vaccines. The predicted results showed that among 686 amino acids in the construct 1, 268 (39.07%), 135 (19.68%), 66 (9.62%) and 217 (31.63%) amino acids are involved in α-helix, extended strand, beta turn, and random coil, respectively. Our predicted outputs revealed that among 607 amino acids in the construct 2, 205 (33.77%), 132 (21.75%), 63 (10.38%) and 207 (34.10%) amino acids are involved in α-helix, extended strand, beta turn, and random coil, respectively.
