# Supplementary material for: Design of a novel multi-epitope vaccine candidate against hepatitis C virus using structural and nonstructural proteins: An immunoinformatics approach
Source: PLoS One. 2022 Aug 30;17(8):e0272582. doi: 10.1371/journal.pone.0272582 (PMC9426923; doi:10.1371/journal.pone.0272582)
Supplement: S2 Fig — Linear (A and C) and Discontinuous (B and D) B-cell epitopes of the construct 1 (A and B) and construct 2 (C and D) vaccines (colored spheres). (DOCX) [file pone.0272582.s011.docx]

| 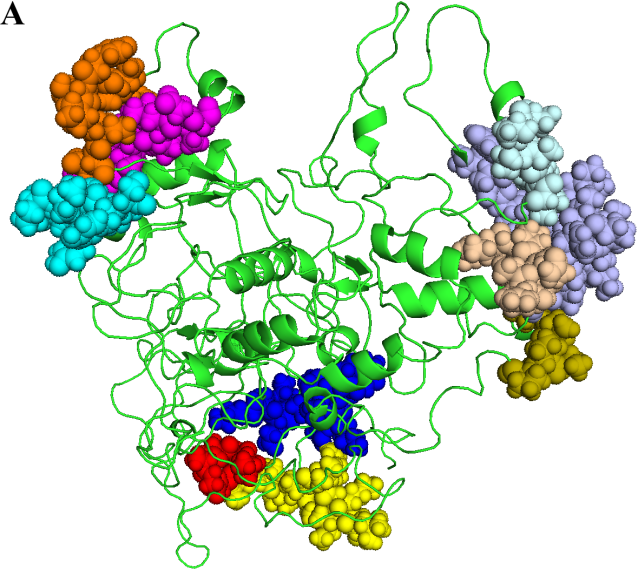 | 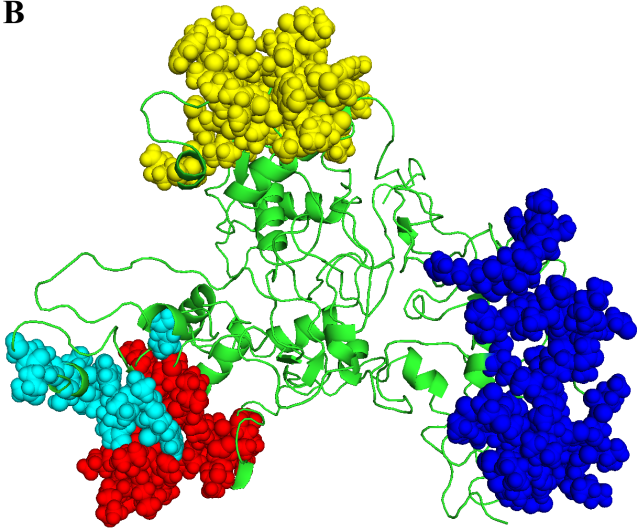 |
| --- | --- |
| 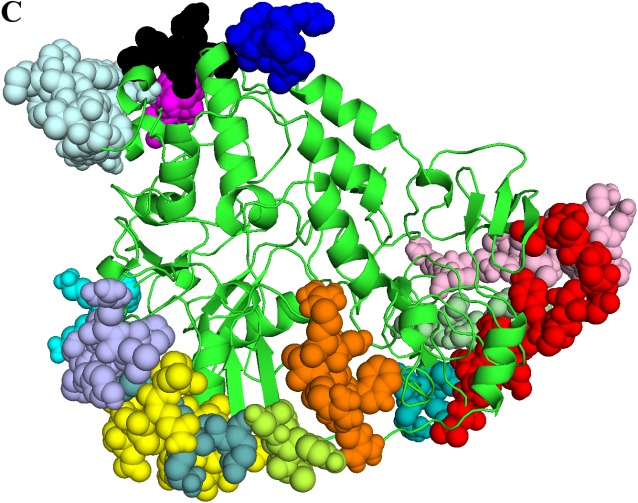 | 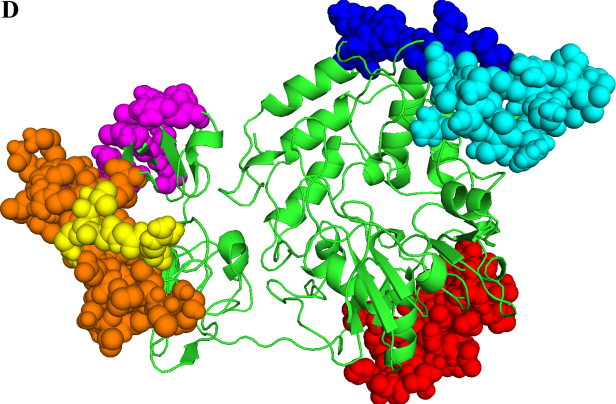 |

**Figure S2:** Linear (A and C) and Discontinuous (B and D) B-cell epitopes of the construct 1 (A and B) and construct 2 (C and D) vaccines (colored spheres).
