# Supplementary material for: Design of a novel multi-epitope vaccine candidate against hepatitis C virus using structural and nonstructural proteins: An immunoinformatics approach
Source: PLoS One. 2022 Aug 30;17(8):e0272582. doi: 10.1371/journal.pone.0272582 (PMC9426923; doi:10.1371/journal.pone.0272582)
Supplement: S3 Fig — The interface residues between two proteins TLRs (orange cartoon) and adjuvants (magenta cartoon) residues (orange and magenta sticks) are labeled. Hydrogen bonds and hydrophobic contacts are presented as green dashed line and arc with spokes radiating, respectively. A and B indicate TLR4-50S ribosomal protein L7/L12 and TLR3-human β-defensin 2 complexes, respectively. (DOCX) [file pone.0272582.s012.docx]

| 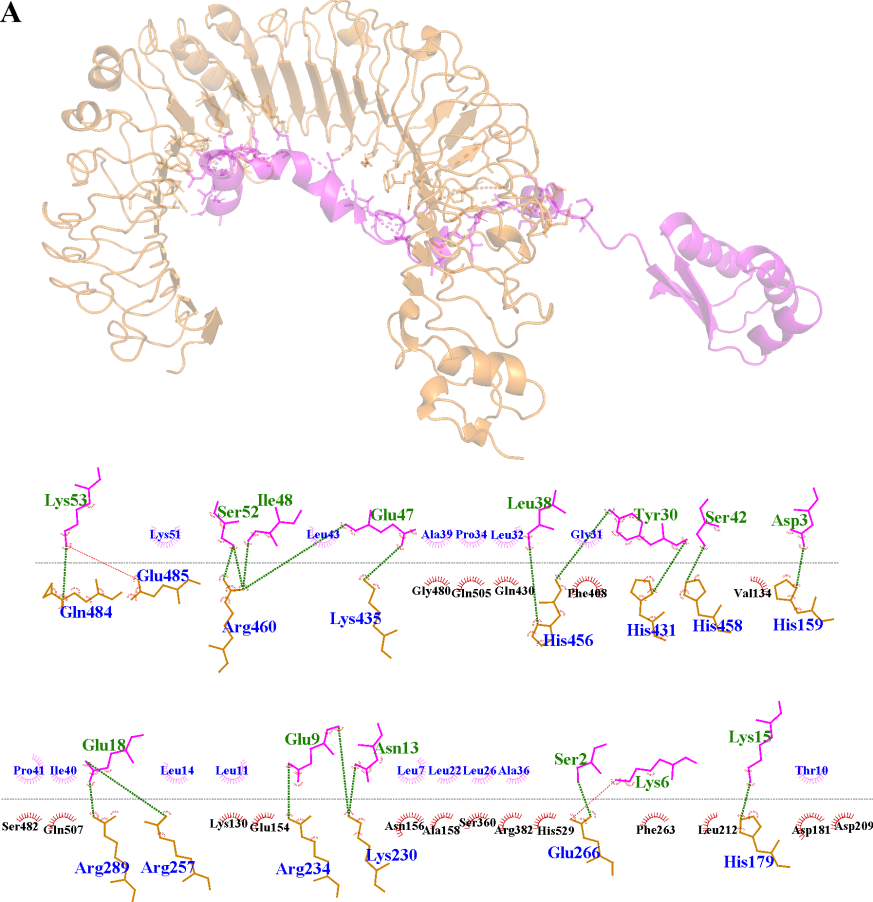 |
| --- |
| 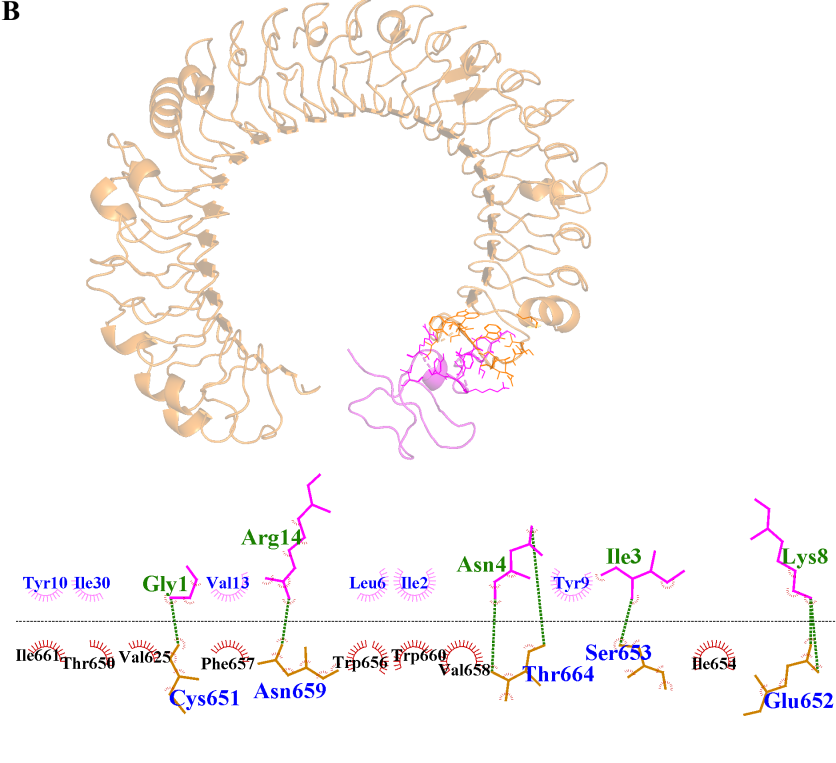 |

**Figure S3:** The 3D view of the molecular docking conformations. The interface residues between two proteins TLRs (orange cartoon) and adjuvants (magenta cartoon) residues (orange and magenta sticks) are labeled. Hydrogen bonds and hydrophobic contacts are presented as green dashed line and arc with spokes radiating, respectively. A and B indicate TLR4-50S ribosomal protein L7/L12 and TLR3-human β-defensin 2 complexes, respectively.
