# Supplementary material for: Design of a novel multi-epitope vaccine candidate against hepatitis C virus using structural and nonstructural proteins: An immunoinformatics approach
Source: PLoS One. 2022 Aug 30;17(8):e0272582. doi: 10.1371/journal.pone.0272582 (PMC9426923; doi:10.1371/journal.pone.0272582)
Supplement: S4 Fig — A and B indicate TLR4-construct 1 and TLR3-construct 2 complexes, respectively. TLRs and costructs are depicted as brown and blue cartoons respectively. (DOCX) [file pone.0272582.s013.docx]

| 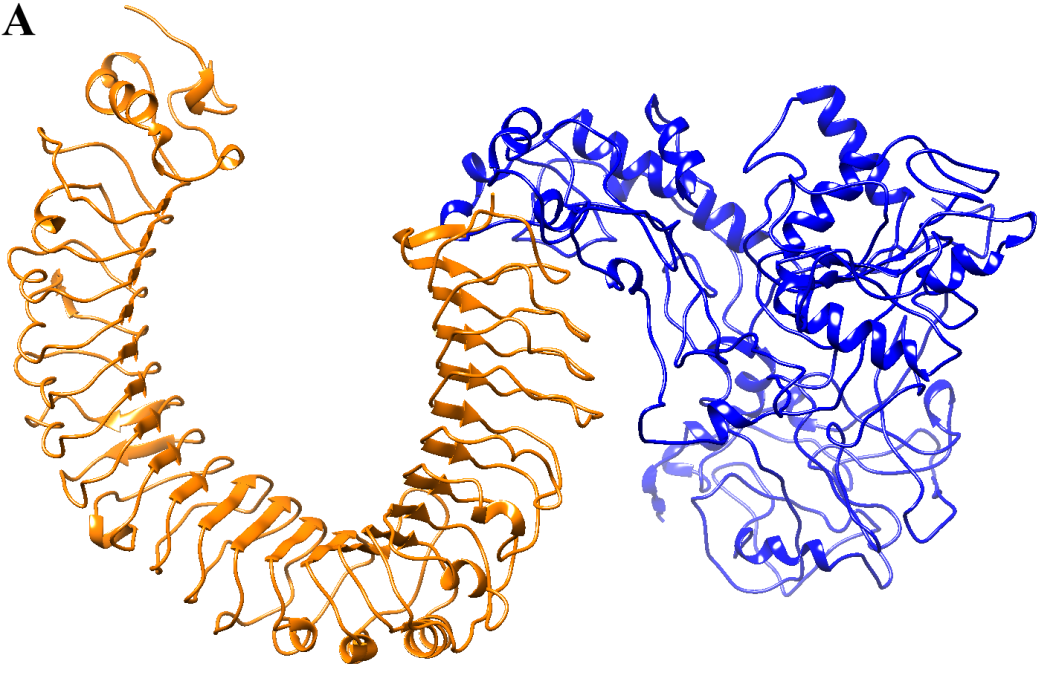 |
| --- |
| 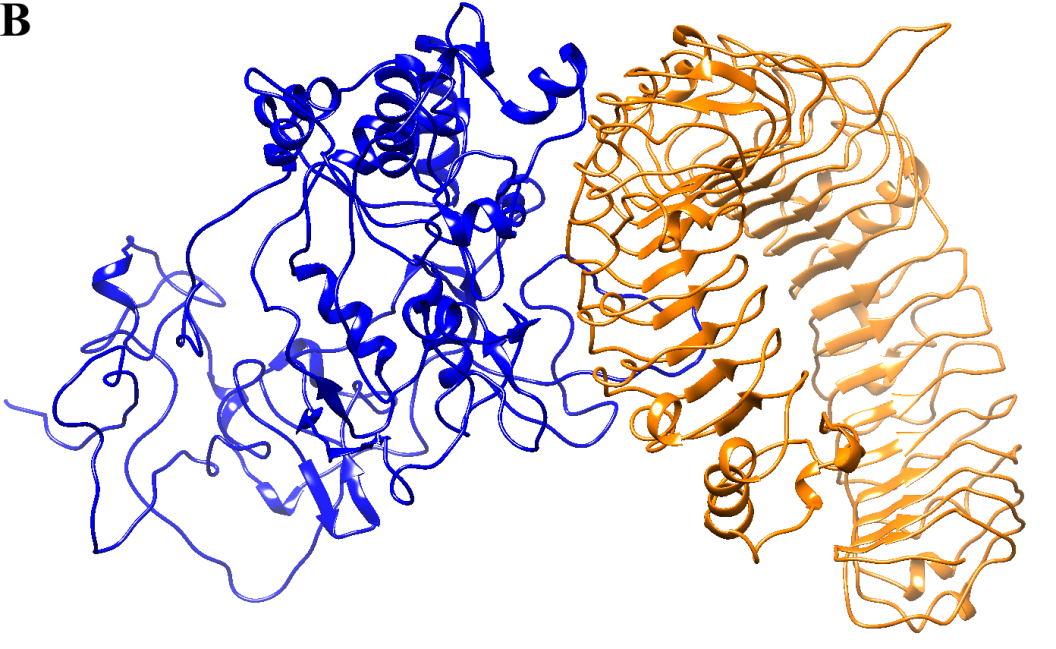 |

**Figure S4**: A and B indicate TLR4-construct 1 and TLR3-construct 2 complexes, respectively. TLRs and costructs are depicted as brown and blue cartoons respectively.
