# Supplementary material for: Design of a novel multi-epitope vaccine candidate against hepatitis C virus using structural and nonstructural proteins: An immunoinformatics approach
Source: PLoS One. 2022 Aug 30;17(8):e0272582. doi: 10.1371/journal.pone.0272582 (PMC9426923; doi:10.1371/journal.pone.0272582)
Supplement: S1 Data — (PDF) [file pone.0272582.s014.pdf]

```
        1        10        20        30        40        50        60
NS3     APITAYAQQTRGLLGCIITSLTGRDKNQVEGEVQIVSTTSQTFLATCVNGVCWTVYHGAG
Q00269  APITAYAQQTRGLLGCIVTSLTGRDKNQVEGEVQVVSTATQSFLATCVNGVCWTVFHGAG
P29846  APITAYAQQTRGLFGCIITSLTGRDKNQVEGEVQVVSTATQSFLATCINGVCWTVYHGAG
P26663  APITAYSQQTRGLLGCIITSLTGRDKNQVEGEVQVVSTATQSFLATCVNGVCWTVYHGAG
Q9WMX2  APITAYSQQTRGLLGCIITSLTGRDRNQVEGEVQVVSTATQSFLATCVNGVCWTVYHGAG
P26662  APITAYSQQTRGLLGCIITSLTGRDKNQVDGEVQVLSTATQSFLATCVNGVCWTVYHGAG
P26664  APITAYAQQTRGLLGCIITSLTGRDKNQVEGEVQIVSTAAQTFLATCINGVCWTVYHGAG
Q81754  APITAYAQQTRGLLGCIITSLTGRDKNQVEGEVQIVSTATQTFLATCVNGVCWTVYHGAG
Q913V3  APITAYSQQTRGLLGCIITSLTGRDKNQVEGEVQVVSTATQSFLATCVNGACWTVFHGAG
Q03463  APITAYAQQTRGLLGCIITSLTGRDKNQVEGEVQIVSTAAQTFLATCINGVCWTVYHGAG
O9297   APITAYSQQTRGVLGCIITSLTGRDKNQVEGEVQVVSTATQSFLATCINGVCWTVYHGAG
P27958  APITAYAQQTRGLLGCIITSLTGRDKNQVEGEVQIVSTATQTFLATCINGVCWTVYHGAG
Q913D4  APITAYAQQTRGLLGCIITSLTGRDKNQVEGEIQIVSTATQTFLATCINGACWTVYHGAG
O92531  APITAYAQQTRGLVGTIVTSLTGRDKNEVEGEIQVVSTATQSFLATTVNGVLWTVYHGAG
O92529  APITAYAQQTRGLVGTIVTSLTGRDKNEAEGEVQVVSTATQSFLATTINGVLWTVYHGAG
Q68798  APITAYAQQTRGLLGTIVTSLTGRDKNEAAGEIQILSTATQTFLATCVNGVCWTVYHGAG
Q5I2N3  APITAYAQQTRGLVGTIVTSLTGRDKNEVEGEVQVVSTATQSFLATSINGVMWTVYHGAG
O92530  APITAYCQQTRGLLGTIVTSLTGRDRNVVEGEIQVLSTATQSFLGTAINGVMWTVYHGAG
O91936  APITAYAQQTRGVLGAIIVSLTGRDKNEAEGEVQVLSTATQTFLGTCINGVMWTVFHGAG
O92532  APITAYAQQTRGIVGTIVTSLTGRDKNEVEGEIQVVSTATQSFLATAVNGVLWTVYYGAG
O39927  APITAYAQQTRGLVGTIVTSLTGRDKNEVEGEVQVVSTDTQSFVATSINGVMWTVYHGPG
O39929  APITAYAQQTRGLFSTIVTSLTGRDTNENCGEVQVLSTATQSFLGTAVNGVMWTVYHGAG
O39928  APITAYAQQTRGVLGAIVLSLTGRDKNEAEGEVQFLSTATQTFLGICINGVMWTLFHGAG
Q9QAX1  APITAYAQQTRGLLGTIVVSMTGRDKTEQAGEIQVLSTVTQSFLGTTISGILWTVFHGAG
P26660  APITAYAQQTRGLLGTIVVSMTGRDKTEQAGEIQVLSTVTQSFLGTTISGVLWTVYHGAG
Q9DHD6  APITAYTQQTRGLLGAIVVSLTGRDKNEQAGQVQVLSSVTQSFLGTSISGVLWTVYHGAG
Q68801  APITAHAQQTRGLFGTIVTSLTGRDKNIVTGEIQVLSTSTQTFLGTSVGGVMWTVYHGAG
Q81258  APITAYAQQTRGLLGTIVTSLTGRDKNVVTGEVQVLSTATQTFLGTTVGGVIWTVYHGAG
Q81495  APITAYAQQTRGLLGTIVTSLTGRDKNVVAGEVQVLSTATQTFLGTTVGGVMWTVYHGAG
P26661  APITAYTQQTRGLLGAIVVSLTGRDKNEQAGQVQVLSSVTQTFLGTSISGVLWTVYHGAG
Q99IB8  APITAYAQQTRGLLGAIVVSMTGRDRTEQAGEVQILSTVSQSFLGTTISGVLWTVYHGAG
Q68749  APITAYAQQTRGLLSAIVVSMTGRDKTDQAGEIQVLSTVTQSFLGTSISGVLWTVFHGAG
Q81487  SPISAYAQQTRGLFGTIVTSLTGRDKNVVTGEVQVLSTATQTFLGTTVGGVMWTVYHGAG
Q69422  APFTLQCLSERGTLSAMAVVMTGIDPRTWTGTIFRLGSLATSYMGFVCDNVLYTAHHGSK
```

```
                 70        80        90       100       110       120
NS3     AKSLAGPKGPILQMYTNVDQDLVGWPAPQGTRSLTPCTCGSSDLYLVTRHADVIPVRRRG
Q00269  SKTLAGPKGPITQMYTNVDQDLVGWHAPPGARSLTPCTCGSSDLYLVTRHADVIPVRRRG
P29846  SKTLAGPKGPITQMYTNVDQDLVGWHAPQGARSLTPCTCGSSDLYLVTRHADVIPVRRRG
P26663  SKTLAAPKGPITQMYTNVDQDLVGWPKPPGARSLTPCTCGSSDLYLVTRHADVIPVRRRG
Q9WMX2  SKTLAGPKGPITQMYTNVDQDLVGWQAPPGARSLTPCTCGSSDLYLVTRHADVIPVRRRG
P26662  SKTLAGPKGPITQMYTNVDQDLVGWPAPPGARSMTPCTCGSSDLYLVTRHADVVPVRRRG
P26664  TRTIASPKGPVIQMYTNVDQDLVGWPAPQGSRSLTPCTCGSSDLYLVTRHADVIPVRRRG
Q81754  SRTIASASGPVIQMYTNVDQDLVGWPAPQGARSLTPCTCGASDLYLVTRHADVIPVRRRG
Q913V3  SKTLAGPKGPITQMYTNVDLDLVGWQAPPGSRSLTPCTCGSSDLYLVTRHADVIPVRRRG
Q03463  TRTIASPKGPVIQMYTNVDQDLVGWPAPQGARSLTPCSCGSSDLYLVTRHADVIPVRRRG
O9297   SKTLAGPKGPITQMYTNVDLDLVGWQAPPGARSMTPCSCGSSDLYLVTRHADVIPVRRRG
P27958  TRTIASPKGPVIQTYTNVDQDLVGWPAPQGSRSLTPCTCGSSDLYLVTRHADVIPVRRRG
Q913D4  SRTIASASGPVVRMYTNVDQDLVGWPAPQGARSLTPCTCGASDLYLVTRHADVIPVRRRG
O92531  SKTLAGPKGPICQMYTNVDQDLVGWPAPPGARSLTPCTCGSSDLYLVTRNADVIPARRRG
O92529  SKNLAGPKGPVCQMYTNVDQDLVGWPAPLGARSLAPCTCGSSDLYLVTRGADVIPARRRG
Q68798  SKTLAGPRGPVCQMYTNVDQDMVGWPAPAGTRSYTPCTCGASDLYLITRQADVIPARRRG
Q5I2N3  SKTLAGPKGPVCQMYTNVDKDLVGWPSPPGARSLTPCTCGSSDLYLVTREADVIPARRRG
O92530  SKTLAGPKGPVCQMYTNVDQDMVGWPAPPGTRSLTPCTCGASDLYLVTRNADVIPARRRG
O91936  AKTLAGPKGPVVQMYTNVDKDLVGWPTPPGTRSLTPCTCGSADLYLVTRHADVVPARRRG
O92532  SKTLAGPKGPVCQMYTNVDQDLVGWPAPAGARSLTPCSCGSSDLYLVTRNADVIPARRRG
O39927  FKTLAGPKGPVCQMYTNVDLDLVGWPSPPGARSLTPCNCGSSDLYLVTREADVIPARRRG
O39929  AKTISGPKGPVNQMYTNVDQDLVGWPAPPGVRSLAPCTCGSADLYLVTRHADVIPVRRRG
O39928  SKTLAGPKGPVVQMYTNVDKDLVGWPSPPGKGSLTRCTCGSADLYLVTRHADVIPARRRG
Q9QAX1  NKTLAGSRGPVTQMYSSAEGDLVGWPSPPGTRSLDPCTCGAVDLYLVTRNADVIPARRQG
P26660  NKTLAGSRGPVTQMYSSAEGDLVGWPSPPGTKSLEPCTCGAVDLYLVTRNADVIPARRRG
Q9DHD6  NKTLASPRGPVTQMYTSAEGDLVGWPSPPGTKSLDPCTCGAVDLYLVTRNADVIPVRRKD
Q68801  SRTLAGNKRPALQMYTNVDQDLVGWPSPPGAKSLVPCTCGSADLYLITRDADVLPARRRG
Q81258  SRTLAGAKHPALQMYTNVDQDLVGWPAPPGAKSLEPCACGSSDLYLVTRDADVIPARRRG
Q81495  SRTLAGVKHPALQMYTNVDQDLVGWPAPPGAKSLEPCTCGSADLYLVTRDADVIPARRRG
P26661  NKTLAGPKGPVTQMYTSAEGDLVGWPSPPGTKSLDPCTCGAVDLYLVTRNADVIPVRRKD
Q99IB8  NKTLAGLRGPVTQMYSSAEGDLVGWPSPPGTKSLEPCKCGAVDLYLVTRNADVIPARRRG
Q68749  NKTLAGSRGPVTQMYSSAEGDLVGWPSPPGTRSLEPCTCGAVDLYLVTRNADVIPARRRG
Q81487  SRTLAGNKRPALQMYTNVDQDLVGWPAPAGTKSLDPCTCGSSDLYLVTREADVLPARRRG
Q69422  GRRLAHPTGSIHPITVDAANDQDIYQPPCGAGSLTRCSCGETKGYLVTRLGSLVEVNKSD
```

```
                130       140       150       160       170       180
NS3     DTRGSLLSPRPISYLKGSSGGPLLCPAGHAVGIFRAAVCTRGVAKAVDFVPVESMETVMR
Q00269  DGRGSLLSPRPVSYLKGSSGGPLLCPSGHAVGIFRAAVCTRGVAKAVDFIPVESMETTMR
P29846  DSRGSLLSPRPISYLKGSSGGPLLCPSGHVVGIFRAAVCTRGVAKAVDFVPVESMETTMR
P26663  DSRGSLLSPRPVSYLKGSSGGPLLCPFGHAVGIFRAAVCTRGVAKAVDFVPVESMETTMR
Q9WMX2  DSRGSLLSPRPVSYLKGSSGGPLLCPSGHAVGIFRAAVCTRGVAKAVDFVPVESMETTMR
P26662  DSRGSLLSPRPISYLKGSSGGPLLCPSGHVVGIFRAAVCTRGVAKAVDFIPVESMETTMR
P26664  DSRGSLLSPRPISYLKGSSGGPLLCPAGHAVGIFRAAVCTRGVAKAVDFIPVENLETTMR
Q81754  DNRGSLLSPRPISYLKGSSGGPLLCPMGHAVGIFRAAVCTRGVAKAVDFVPVESLETTMR
Q913V3  DSRGSLLSPRPVSYLKGSSGGPLLCPSRHAVGIFRAAVCTRGVAKAVDFIPVESMETTMR
Q03463  DSRGSLLSPRPISYLKGSSGGPLLCPAGHVVGIFRAAVCTRGVAKAVDFIPVESLETTMR
O9297   DSRGSLLSPRPVSYLKGSSGGPLLCPSGHVVGVFRAAVCTRGVAKAVDFIPVESMETTMR
P27958  DSRGSLLSPRPISYLKGSSGGPLLCPTGHAVGLFRAAVCTRGVAKAVDFIPVENLETTMR
Q913D4  DNRGSLLSPRPISYLKGSSGGPLLCPMGHVAGIFRAAVCTRGVAKAVDFVPVESLETTMR
O92531  DTRAALLSPRPISTLKGSSGGPMLCPSGHVAGIFRAAVCTRGVAKSLDFVPVENMQSTAR
O92529  DTRAALLSPRPISTLKGSSGGPLMCPSGHVVGLFRAAVCTRGVAKALDFIPVENMDTTMR
Q68798  DNRAGLISPRPISTLKGSSGGPLLCPSGHVVGLFRAAVCTRGVAKALDFVPCEAMDATTR
Q5I2N3  DNRAALLSPRPISTLKGSSGGPVMCPSGHVVGLFRAAVCTRGVAKSLDFIPVENMETTMR
O92530  DTRAGLLSPRPLSTLKGSSGGPLMCPSDHVVGLFRAAVCTRGVAKALDFVPVENMETTMR
O91936  DTRASLLSPRPISYLKGSSGGPVMCPSGHVVGVFRAAVCTRGVAKALDFIPVENLETTMR
O92532  DNRAALLSPRPISTLKGSSGGPMLCPSGHVAGIFRAAVCTRGVAKSLDFAPVESMQSSQR
O39927  DSRAALLSPRPISTLKGSSGGPIMCPSGHVVGLFRAAVCTRGVAKSLDFIPVENMETTMR
O39929  DTRGALLSPRPISILKGSSGGPLLCPMGHRAGIFRAAVCTRGVAKAVDFVPVESLETTMR
O39928  DTRASLLSPRPISYLKGSSGGPIMCPSGHVVGVFRAAVCTRGVAKALEFVPVENLETTMR
Q9QAX1  DRRGALLSPRPLSSLKGSSGGPVLCPRGHAVGIFRAAICTRGAAKSIDFIPIESLDVIIR
P26660  DKRGALLSPRPLSTLKGSSGGPVLCPRGHAVGVFRAAVCSRGVAKSIDFIPVETLDIVTR
Q9DHD6  DRRGALLSPRPLSTLKGSSGGPVLCPRGHAVGLFRAAVCARGVAKSIDFIPVESLDIARR
Q68801  DSTASLLSPRPLACLKGSSGGPIMCPSGHVAGIFRAAVCTRGVAKALQFIPVESLSAQTR
Q81258  DSTASLLSPRPLACLKGSSGGPVMCPSGHVAGIFRAAVCTRGVAKSLQFIPVETLSTQAR
Q81495  DSTASLLSPRPLARLKGSSGGPVMCPSGHVAGIFRAAVCTRGVAKALQFIPVETLSTQAR
P26661  DRRGALLSPRPLSTLKGSSGGPVLCSRGHAVGLFRAAVCARGVAKSIDFIPVESLDVATR
Q99IB8  DKRGALLSPRPISTLKGSSGGPVLCPRGHVVGLFRAAVCSRGVAKSIDFIPVETLDVVTR
Q68749  DRRGALLSPRPLSSLKGSSGGPVLCPRGHAVGIFRAAVCSRGVAKSIDFIPVESLDVVTR
Q81487  DSTASLLSTRPLSCLKGSSGGPVMCPSGHVVGIFRAAVCTRGVAKALQFIPVETLSTQVR
Q69422  DPYWCVCGALPMAVAKGSSGAPILCSSGHVIGMFTAARNSGGSVSQIRVRPLVCAGYHPQ
```

```
                190       200       210       220       230       240
NS3     SPTFTDNSTPPAVPQTFQVAHLHAPTGSGKSTKVPAAYAAQGYKVLVLNPSVAATLGFGA
Q00269  SPVFTDNSSPPAVPQTFQVAHLHAPTGSGKSTKVPAAYAAQGYKVLVLNPSVAATLGFGA
P29846  SPVFTDNSSPPAVPQAFQVAHLHAPTGSGKSTKVPAAYAAQGYKVLVLNPSVAATLGFGA
P26663  SPVFTDNSSPPAVPQSFQVAHLHAPTGSGKSTKVPAAYAAQGYKVLVLNPSVAATLGFGA
Q9WMX2  SPVFTDNSSPPAVPQTFQVAHLHAPTGSGKSTKVPAAYAAQGYKVLVLNPSVAATLGFGA
P26662  SPVFTDNSSPPAVPQTFQVAHLHAPTGSGKSTKVPAAYAAQGYKVLVLNPSVAATLGFGA
P26664  SPVFTDNSSPPVVPQSFQVAHLHAPTGSGKSTKVPAAYAAQGYKVLVLNPSVAATLGFGA
Q81754  SPVFTDNSSPPTVPQSYQVAHLHAPTGSGKSTKVPAAYAAQGYKVLVLNPSVAATLGFGA
Q913V3  SPVFTDNSSPPAVPQTFQVAHLHAPTGSGKSTKVPAAYAAQGYKVLVLNPSVAATLGFGA
Q03463  SPVFTDNSSPPAVPQSFQVAHLHAPTGSGKSTKVPAAYAAQGYKVLVLNPSVAATLGFGA
O9297   SPVFTDNSSPPAVPQTFQVAHLHAPTGSGKSTKVPAAYAAQGYKVLVLNPSVAATLGFGA
P27958  SPVFTDNSSPPAVPQSFQVAHLHAPTGSGKSTKVPAAYAAKGYKVLVLNPSVAATLGFGA
Q913D4  SPVFTDNSSPPTVPQSYQVAHLHAPTGSGKSTKVPAAYAAQGYKVLVLNPSVAATLGFGA
O92531  SPSFSDNTTPPAVPQTYQVGYLHAPTGSGKSTKVPAAYAAQGYKVLVLNPSVAATLGFGS
O92529  SPVFTDNSSPPAVPQTYQVGYLHAPTGSGKSTRVPAAYATQGYKVLVLNPSVAATLSFGA
Q68798  SPTFTDNSTPPAVPQAYQVGYLHAPTGSGKSTKVPVAYASQGYKVLVLNPSVAATLSFGS
Q5I2N3  SPSFTDNSTPPAVPQTYQVGYLHAPTGSGKSTRVPAAYASQGYKVLVLNPSVAATLSFGS
O92530  SPVFTDNSTPPAVPQTYQVGYLHAPTGSGKSTKVPAAYASQGYKVLVLNPSVAATLGFGS
O91936  SPVFTDNSTPPAVPHEFQVGHLHAPTGSGKSTKVPAAYAAQGYKVLVLNPSVAATLGFGA
O92532  SPSFSDNTSPPAVPQTYQVGYLHAPTGSGKSTKVPAAYASQGYKVLVLNPSVAATLGFGS
O39927  SPSFTDNSTPPAVPQTYQVGYLHAPTGSGKSTRVPAAYASQGYKVLVLNPSVAATLSFGS
O39929  SPVFTDNSTPPAVPQTYQVAHLHAPTGSGKSTKVPAAHAAQGYKVLVLNPSVAATLGFGV
O39928  SPVFTDNSTPPAVPHEFQVGHLHAPTGSGKSTKVPAAYAAQGYKVLVLNPSVAATFGFGA
Q9QAX1  SPNFTDNSSPPAVPQTYQVGYLHAPTGSGKSTKVPASYAAQGYKVLVLNPSVAATLGFGA
P26660  SPTFSDNSTPPAVPQTYQVGYLHAPTGSGKSTKVPVAYAAQGYKVLVLNPSVAATLGFGA
Q9DHD6  TPSFSDNSTPPAVPQTYQVGYLHAPTGSGKSTKVPAAYTSQGYKVLVLNPSVAATLGFGA
Q68801  SPSFSDNSTPPAVPQTFQVGYLHAPTGSGESTKVPASYVAQGYTVLVLNPSVAATLGFGR
Q81258  SPSFSDNSTPPAVPQSYQVGYLHAPTGSGKSTKVPAAYVAQGYNVLVLNPSVAATLGFGS
Q81495  SPSFSDNSTPPAVPQSYQVGYLHAPTGSGKSTKVPAAYVAQGYNVLVLNPSVAATLGFGS
P26661  TPSFSDNSTPPAVPQSYQVGYLHAPTGSGKSTKVPAAYASQGYKVLVLNPSVAATLGFGA
Q99IB8  SPTFSDNSTPPAVPQTYQVGYLHAPTGSGKSTKVPVAYAAQGYKVLVLNPSVAATLGFGA
Q68749  SPNFTDNSTPPAVPQTYQVGYLHAPTGSGKSTKVPAAYAAQGYKVLVLNPSVAATLGFGA
Q81487  SPSFSDNSTPPAVPESYQVGYLHAPTGSGKSTKVPAAYVAQGYSVLVLNPSVAATLGFGT
Q69422  YTAHATLDTKPTVPNEYSVQILIAPTGSGKSTKLPLSYMQEKYEVLVLNPSVATTASMPK
```

```
                250       260       270       280       290       300
NS3     YMSKAHGIDPNIRTGVRTITTGSPITYSTYGKFLADGGCSGGAYDIIICDECHSTDATSI
Q00269  YMSKAHGTDPNIRTGVRTITTGAPITYSTYGKFLADGGCSGGAYDIIICDECHSTDSTTI
P29846  YMSKAHGVDPNIRTGVRTITTGAPITYSTYGKFLADGGCSGGAYDIIMCDECHSTDSTTI
P26663  YMSKAHGIDPNIRTGVRTITTGAPVTYSTYGKFLADGGCSGGAYDIIICDECHSTDSTTI
Q9WMX2  YMSKAHGIDPNIRTGVRTITTGAPITYSTYGKFLADGGCSGGAYDIIICDECHSTDSTTI
P26662  YMSKAHGIEPNIRTGVRTITTGGPITYSTYCKFLADGGCSGGAYDIIICDECHSTDSTTI
P26664  YMSKAHGIDPNIRTGVRTITTGSPITYSTYGKFLADGGCSGGAYDIIICDECHSTDATSI
Q81754  YMSKAHGIDPNVRTGVRTITTGSPITHSTYGKFLADGGCSGGAYDIIICDECHSVDATSI
Q913V3  YMSKAHGIDPNIRTGVRAITTGAPITYSTYGKFLADGGCSGGAYDIIICDECHSTDSTTI
Q03463  YMSKAHGIDPNIRTGVRTITTGSPITYSTYGKFLADGGCSGGAYDIIICDECHSTDATSV
O9297   YMSKAHGIDPNIRTGVRTITTGGSITYSTYGKFLADGGCSGGAYDIIICDECHSTDSTTI
P27958  YMSKAHGVDPNIRTGVRTITTGSPITYSTYGKFLADAGCSGGAYDIIICDECHSTDATSI
Q913D4  YMSKAHGIDPNVRTGVRTITTGSPITYSTYGKFLADGGCPGGAYDIIICDECHSVDATSI
O92531  YMSTAHGIDPNIRTGVRTITTGGAITYSTYGKFLADGGCSGGAYDIIICDECHSTDPTTV
O92529  YMSKAHGIDPNIRTGVRTITTGGPVTYSTYGKFLADGGCSGGAYDIIICDECHSTDPTTV
Q68798  YLSRAHGIDPNIRTGVRTITTGAPITYSTYGKFLADGGCSGGAYDVIICDECHSTDPTTV
Q5I2N3  YMRQAYGVEPNVRTGVRTVTTGGAITYSTYGKFLADGGCSGGAYDIIICDECHSTDPTTV
O92530  YMSTAHGIDPNIRTGVRTITTGGPITYSTYGKFLADGGCSGGAYDIIICDECHSTDPTTV
O91936  YMSRAYGVDPNIRTGVRTVTTGAAITYSTYGKFLADGGCSGGAYDVIICDECHSQDATTI
O92532  YMSTSHGIDPNIRTGVRTITTGGAITYSTYGKFLADGGCSGGAYDVIICDECHSTDPTTV
O39927  YMRQAYGVEPNIRTGVRTVTTGGAITYSTYGEFLADGGCSGGAYDIIICDECHSTDPTTV
O39929  YMSKAYGIDPNIRSGVRTITTGAPITYSTYGKFLADGGCSGGAYDIIICDECYSTDSTTI
O39928  YMSRAYGVDPNIRTGVRTVTTGAGITYSTYGKFFADGGCSGGAYDVIICDECHSQDATTI
Q9QAX1  YMSKAHGINPNIRTGVRTVTTGESITYSTYGKFLADGGCSGGAYDVIICDECHSVDATTI
P26660  YLSKAHGINPNIRTGVRTVTTGAPITYSTYGKFLADGGCAGGAYDIIICDECHAVDSTTI
Q9DHD6  YMSKAHGINPNIRTGVRTVTTGDSITYSTYGKFLADGGCSAGAYDIIICDECHSVDATTI
Q68801  FMSHAYGIDPNVRTGTRTITTGAKLTYSTYGKFLADGGCSGGAYDVIICDECHAQDATSI
Q81258  FMSRAYGIDPNIRTGNRTVTTGAKLTYSTYGKFLADGGCSGGAYDVIICDECHAQDATSI
Q81495  FMSRAYGIDPNIRTGNRTVTTGAKLTYSTYGKFLAGGGCSGGAYDVIICDDCHAQDATSI
P26661  YMSKAHGINPNIRTGVRTVTTGDSITYSTYGKFIADGGCAAGAYDIIICDECHSVDATTI
Q99IB8  YLSKAHGINPNIRTGVRTVMTGEAITYSTYGKFLADGGCASGAYDIIICDECHAVDATSI
Q68749  YMSKAYGINPNIRTGVRTVTTGDAITYSTYGKFLADGGCSGGAYDVIICDECHSVDSTTI
Q81487  YMSKAYGIDPNIRTGTRTITTGAKLTYSTYGKFLADGGCSGGAYDVIICDECHAQDATSI
Q69422  YMHATYGVNPNCYFNGKCTNTGASLTYSTYGMYLT.GACSRN.YDVIICDECHATDATTV

                310       320       330       340       350       360
NS3     LGIGTVLDQAETCGARMVVLATATPPGSVTVPHPNIEEVALSNIGEIPFYGKAIPLEVIK
Q00269  LGIGTVLDQAETAGARLVVLATATPPGSVTVPHPNIEEVALSNTGEIPFYGKAIPLEAIK
P29846  LGIGTVLDQAETAGARLVVLATATPPGSVTVPHPNIEEIALSNTGEIPFYGKAIPIETIK
P26663  LGIGTVLDQAETAGARLVVLATATPPGSVTVPHPNIEEVALSNTGEIPFYGKAIPIEAIR
Q9WMX2  LGIGTVLDQAETAGARLVVLATATPPGSVTVPHPNIEEVALSSTGEIPFYGKAIPIETIK
P26662  LGIGTVLDQAETAGARLVVLATATPPGSITVPHPNIEEVALSNTGEIPFYGKAIPIEAIK
P26664  LGIGTVLDQAETAGARLVVLATATPPGSVTVPHPNIEEVALSTTGEIPFYGKAIPLEVIK
Q81754  LGIGTVLDQAETAGVRLTILATATPPGSVTVPHSNIEEVALSTEGEIPFYGKAIPLNYIK
Q913V3  LGIGTVLDQAETAGARLVVLATATPPGSVTVPHPNIEEVALSNAGEIPFYGKAIPIEVIK
Q03463  LGIGTVLDQAETAGARLVVLATATPPGSITVPHANIEEVALSTTGEIPFYGKAIPLEAIK
O9297   LGIGTVLDQAETAGARLVVLATATPPGSVTVPHPNIEEIGLSNNGEIPFYGKAIPIEAIK
P27958  SGIGTVLDQAETAGARLVVLATATPPGSVTVSHPNIEEVALSTTGEIPFYGKAIPLEVIK
Q913D4  LGIGTVLDQAETAGVRLTVLATATPPGLVTVPHSNIEEVALSADGEKPFYGKAIPLNYIK
O92531  LGIGTVLDQAETAGVRLTVLATATPPGSVTVPHPNITEVALSSTGEVPFYGKAIPLEYIK
O92529  LGIGTVLDQAETAGVRLTVLATATPPGSVTVPHPNITETALPTTGEIPFYGKCIPLEFIK
Q68798  LGIGTVLDQAETAGCRLTVLATATPPGSVTVPHPNIQETALPLTGEVPFYGKAIPLEYIK
Q5I2N3  LGIGTVLDQAETAGARLTVLATATPPGSITVPHPNITETALPTTGEIPFYGKAIPLEYIK
O92530  LGIGTVLDQAETAGVRLTVLATATPPGSVTVPHPNITETALPSTGEVPFYGKAIPLECIK
O91936  LGIGTVLDQAETAGARLVVLATATPPGSVTTPHPNIEEVALPSEGEIPFYGRAIPLALIK
O92532  SGIGTVLDQAETSGVRLTVLATATPPGSVTVPHPNITESALPTTGEIPFYGKAVPLEYIK
O39927  LGVGTVLDQAETAGVRLTVLPTATPPGSVTVPHPNITETALPTTGEIPFYGKAIPLEYIK
O39929  LGIGTVLDQAETAGVRLTVLATATPPGSVTTPHSNIEEVALPTTGEIPFYGKAIPLELIK
O39928  LGIGTVLDQAETAGARLVVLATAIPPGSVTTPHPNIEEVALPSEGEIPFYGRAIPLVLIK
Q9QAX1  LGIGTVLDQAETAGARLTVLATATPPGSVTTPHPNIEEVALGHEGEIPFYGKAIPLSQIK
P26660  LGIGTVLDQAETAGVRLTVLATATPPGSVTTPHPNIEEVALGQEGEIPFYGRAIPLSYIK
Q9DHD6  LGIGTVLDQAETAGVRLVVLATATPPGTVTTPHANIEEVALGHEGEIPFYGKAIPLASIK
Q68801  LGIGTVLDQAETAGARLVVLATATPPGSITVPHSNIEEVALTGEGEIPFYGRAIPLGVIK
Q81258  LGIGTVLDQAETAGVRLTVLATATPPGSITVPHSNIEEVALGSEGEIPFYGKAIPIALLK
Q81495  LGIGTVLDQAETAGVRLTVLATATPPGSITVPHSNIEEVALGSEGEIPFYGKAIPIACIK
P26661  LGIGTVLDQAETAGVRLVVLATATPPGTVTTPHSNIEEVALGHEGEIPFYGKAIPLAFIK
Q99IB8  LGIGTVLDQAETAGVRLTVLATATPPGSVTTPHPDIEEVGLGREGEIPFYGRAIPLSCIK
Q68749  LGIGTVLDQAETAGVRLTVLATATPPGSVTTPHPNIEEVALGHEGEIPFYGKAIPLSAIK
Q81487  LGIGTVLDQAETAGVRLTVLATATPPGSITVPHPNIEEVGLTSDGEIPFYGKALPLAMIK
Q69422  LGIGKVLTEAPSKNVRLVVLATATPPGVIPTPHANITEIQLTDEGTIPFHGKKIKEENLK
```

```
                 370       380       390       400       410       420
                   .         .         .         .         .         .
NS3       GGRHLIFCHSKKKCDELAAKLTSLGLNAVAYYRGLDVSVIPLSGDVVVVSTDALMTGFTG
Q00269    GGRHLIFCHSKKKCDELAAKLSGLGINAVAYYRGLDVSVIPTSGDVVIVATDALMTGYTG
P29846    GGRHLIFCHSKKKCDELAAKLSALGIHAVAYYRGLDVSVIPASGNVVVVATDALMTGFTG
P26663    GGRHLIFCHSKKKCDELAAKLSGLGINAVAYYRGLDVSVIPTIGDVVVVATDALMTGYTG
Q9WMX2    GGRHLIFCHSKKKCDELAAKLSGLGLNAVAYYRGLDVSVIPTSGDVIVVATDALMTGFTG
P26662    GGRHLIFCHSKKKCDELAAKLTGLGLNAVAYYRGLDVSVIPTSGDVVVVATDALMTGFTG
P26664    GGRHLIFCHSKKKCDELAAKLVALGINAVAYYRGLDVSVIPTSGDVVVVATDALMTGYTG
Q81754    GGRHLIFCHSKKKCDELAAKLVGLGVNAVAFYRGLDVSVIPTTGDVVVVATDALMTGYTG
Q913V3    GGRHLIFCHSKKKYDELAAKLSALGLNAVAYYRGLDVSVIPTNGDVVVVATDALMTGFTG
Q03463    GGRHLIFCHSKKKCDELAAKLVALGVNAVAYYRGLDVSVIPTSGDVVVVATDALMTGYTG
O9297     GGRHLIFCHSKKKCDELAAKLTGLGLNAVAYYRGLDVSVIPPIGDVVVVATDALMTGFTG
P27958    GGRHLIFCHSKKKCDELAAKLVALGINAVAYYRGLDVSVIPTSGDVVVVSTDALMTGFTG
Q913D4    GGRHLIFCHSKKKCDELAAKLVGLGVNAVAFYRGLDVSVIPTTGDVVVVATDALMTGFTG
O92531    GGRHLIFCHSKKKCDELAKQLTSLGLNAVAFYRGVDVSVIPTSGDVVVCATDALMTGYTG
O92529    GGRHLIFCHSKKKCDELSKQLTSLGLNAVAFYRGVDVAVIPTSGDVVVCATDALMTGYTG
Q68798    GGRHLIFCHSKKKCDELAAQLRTLGLNAVAFYRGVDVSVIPTSGDVVVCATDALMTGYTG
Q5I2N3    GGRHLIFCHSKKKCDELAGKLKSLGLNAVAFYRGVDVSVIPTSGDVVICATDALMTGYTG
O92530    GGRHLIFCHSKKKCDELAKQLRTLGLNAVAFYRGVDVSVIPTAGDVVVCATDALMTGYTG
O91936    GGRHLIFCHSKKKCDELAKQLTSQGVNAVAYYRGLDVAVIPATGDVVVCSTDALMTGFTG
O92532    GGRHLIFCHPKKKCDELAKQLVSLGLNAVAFYRGVDVSVIPTSGDVVVCATDALMTGYTG
O39927    GGRHLIFCHSKKKCDELAGKLKSLGLNAVAFYRGVDVSVIPTSGDVVVCATDALMTGYTG
O39929    GGRHLIFCHSKKKCDELARQLTSLGLNAVAYYRGLDVSVIPTSGDVVVCATDALMTGFTG
O39928    GGRHLIFCHSKKKCDELAKQLTSLGVNAVAYYRGLDVAVIPATGDVVVCSTDALMTGFTG
Q9QAX1    GGRHLIFCHSKKKCDELAAALRGMGLNAVAYYRGLDVSVIPTQGDVVVVATDALMTGFTG
P26660    GGRHLIFCHSKKKCDELAAALRGMGLNAVAYYRGLDVSVIPTQGDVVVVATDALMTGFTG
Q9DHD6    GGRHLIFCHSKKKCDELAAALRGMGVNAVAYYRGLDVSVIPTQGDVVVVATDALMTGYTG
Q68801    GGRHLIFCHSKKKCDELAKQLTSLGVNAVAFYRGLDVSVIPTQGDVVVCATDALITGYTG
Q81258    GGRHLIFCHSKKKCDEIASKLRGMGLNAVAYYRGLDVSVIPTTGDVVVCATDALMTGFTG
Q81495    GGRHLIFCHSKKKCDKMASKLRGMGLNAVAYYRGLDVSVIPTTGDVVVCATDALMTGFTG
P26661    GGRHLIFCHSKKKCDELAAALRGMGVNAVAYYRGLDVSVIPTQGDVVVVATDALMTGYTG
Q99IB8    GGRHLIFCHSKKKCDELAAALRGMGLNAVAYYRGLDVSIIPAQGDVVVVATDALMTGYTG
Q68749    GGRHLIFCHSKKKCDELAVALRGMGLNAVAYYRGLDVSIIPTQGDVVVVATDALMTGYTG
Q81487    GGRHLVFCHSKEKCDELASKLRGMGVNAVAFYRGLDVSVIPVSGDVVVCATDALMTGYTG
Q69422    KGRHLIFEATKKHCDELANELARKGITAVSYYRGCDISKIP.EGDCVVVATDALCTGYTG
```

```
                 430       440       450       460       470       480
                   .         .         .         .         .         .
NS3       DFDSVIDCNVCVTQTVDFSLDPTFTIETTTLPQDAVSRSQRRGRTGRGRLGIYRFVTPGE
Q00269    DFDSVIDCNTCVTQTVDFSLDPTFTIETTTVPQDAVSRSQRRGRTGRGRGGIYRFVTPGE
P29846    DFDSVIDCNTCVTQTVDFSLDPTFTIETTTMPQDAVSRSQRRGRTSRGRRGIYRFVTPGE
P26663    DFDSVIDCNTCVTQTVDFSLDPTFTIETTTVPQDAVSRSQRRGRTGRGRRGIYRFVTPGE
Q9WMX2    DFDSVIDCNTCVTQTVDFSLDPTFTIETTTVPQDAVSRSQRRGRTGRGRMGIYRFVTPGE
P26662    DFDSVIDCNTCVTQTVDFSLDPTFTIETTTLPQDAVSRAQRRGRTGRGRSGIYRFVTPGE
P26664    DFDSVIDCNTCVTQTVDFSLDPTFTIETITLPQDAVSRTQRRGRTGRGKPGIYRFVAPGE
Q81754    DFDSVIDCNTCVVQTVDFSLDPTFSIETSTVPQDAVSRSQRRGRTGRGKHGIYRYVSPGE
Q913V3    DFDSVIDCNTCVTQTVDFSLDPTFTIETTTVPQDAVARSQRRGRTGRGRRGIYRFVTPGE
Q03463    DFDSVIDCNTCVTQTVDFSLDPTFTIETTTLPQDAVSRTQRRGRTGRGKPGIYRFVAPGE
O9297     DFDSVIDCNTCVTQTVDFSLDPTFTIETTTVPQDAVSRSQRRGRTGRGRSGIYRFVTPGE
P27958    DFDSVIDCNTCVTQTVDFSLDPTFTIETTTLPQDAVSRTQRRGRTGRGKPGIYRFVAPGE
Q913D4    DFDSVIDCNTCVVQTVDFSLDPIFSIETSTVPQDAVSRSQRRGRTGRGKHGIYRYVSPGE
O92531    DFDSVIDCNVSVTQVVDFSLDPTFTIETTTMPQDAVSRSQRRGRTGRGKHGVYRYVSQGE
O92529    DFDSVIDCNVAVTQVVDFSLDPTFSIETTTVPQDAVSRSQRRGRTGRGKPGVYRFVSQGE
Q68798    DFDSVIDCNVAVTQIVDFSLDPTFSIETTTVPQDAVARSQRRGRTGRGKPGVYRYVSQGE
Q5I2N3    DFDSVIDCNVAVTQVVDFSLDPTFSIETTTVPQDAVSRSQRRGRTGRGKPGVYRFVSQGE
O92530    DFDSVIDCNVAVTQIVDFSLDPTFSIETTTVPQDAVARSQRRGRTGRGKPGVYRYVSQGE
O91936    DFDSVIDCNTTVTQTVDFSLDPTFTIETTTVPQDAVSRSQRRGRTGRGRHGIYRYVSSGE
O92532    DFDSVIDCNVTVTQVVDFSLDPTFTIETTTVPQDAVSRSQRRGRTGRGKHGVYRYVSQGE
O39927    DFDSVIDCNVAVTQVVDFSLDPTFSIETTTVPQDAVSRSQRRGRTGRGKPGVYRFVSQGE
O39929    DFDSVIDCNTSVIQTVDFSLDPTFSIEITTVPQDAVSRSQRRGRTGRGRLGTYRYVTPGE
O39928    DFDSVIDCNSAVTQTVDFSLDPTFTIETTTVPQDAVSRSQRRGRTGRGRHGIYRYVSSGE
Q9QAX1    DFDSVVDCNVAVTQTVDFSLDPTFTVTTQTVPQDAVSRSQRRGRTGRGRLGIYRYVSSGE
P26660    DFDSVIDCNVAVTQVVDFSLDPTFTITTQTVPQDAVSRSQRRGRTGRGRLGIYRYVSTGE
Q9DHD6    DFDSVIDCNVAVTQIVDFSLDPTFTITTQTVPQDAVSRSQRRGRTGRGRLGTYRYVSSGE
Q68801    DFDSVIDCNVAVEQYVDFSLDPTFSIETHTVPQDAVSRSQRRGRTGRGKSGTYRYVSPGE
Q81258    DFDSVIDCNVAVEQYVDFSLDPTFSIETRTAPQDAVSRSQRRGRTGRGRLGTYRYVASGE
Q81495    DFDSVIDCNVAVEQYVDFSLDPTFSIETCTAPQDAVSRSQRRGRTGRGRLGTYRYVTPGE
P26661    DFDSVIDCNVAVSQIVDFSLDPTFTITTQTVPQDAVSRSQRRGRTGRGRLGVYRYVSSGE
Q99IB8    DFDSVIDCNVAVTQAVDFSLDPTFTITTQTVPQDAVSRSQRRGRTGRGRQGTYRYVSTGE
Q68749    DFDSVIDCNVAVTQVVDFSLDPTFTITTQTVPQDSVSRSQRRGRTGRGRLGIYRYVSSGE
Q81487    DFDTVIDCNVAVEQYVDFSLDPTFSIETRTVPQDAVSRSQRRGRTGRGRPGIYRFVTPGE
Q69422    DFDSVYDCSLMVEGTCHVDLDPTFTMGVRVCGVSAIVKGQRRGRTGRGRAGIYYYVDGSC
```

```
                490       500       510       520       530       540
NS3     RPSGMFDSSVLCECYDAGCAWYELTPAETSVRLRAYMNTPGLPVCQDHLEFWESVFTGLT
Q00269  RPSGMFDSSVLCECYDAGCAWYELTPAETTVRLRAYLNTPGLPVCQDHLEFWESVFTGLT
P29846  RPSGMFDSSVLCECYDAGCAWYELTPAETSVRLRAYLNTPGLPVCQDHLEFWESVFTGLT
P26663  RPSGMFDSSVLCECYDAGCAWYELTPAETSVRLRAYLNTPGLPVCQDHLEFWESVFTGLT
Q9WMX2  RPSGMFDSSVLCECYDAGCAWYELTPAETSVRLRAYLNTPGLPVCQDHLEFWESVFTGLT
P26662  RPSGMFDSSVLCECYDAGCAWYELTPAETSVRLRAYLNTPGLPVCQDHLEFWESVFTGLT
P26664  RPSGMFDSSVLCECYDAGCAWYELTPAETTVRLRAYMNTPGLPVCQDHLEFWEGVFTGLT
Q81754  RPSGMFDSVVLCECYDAGCAWYELTPAETTVRLRAYLNTPGLPVCQDHLEFWESVFTGLT
Q913V3  RPSGMFDSSVLCECYDAGCAWYELTPAETSVRLRAYLNTPGLPVCQDHLEFWESVSTGLT
Q03463  RPSGMFDSSILCECYDTGCAWYELTPAETTVRLRAYMNTPGLPVCQDHLEFWEGVFTGLT
O9297   RPSGMFDSSVLCECYDAGCAWYELTPAETSVRLRAYLNTPGLPVCQDHLEFWESVFTGLT
P27958  RPSGMFDSSVLCECYDAGCAWYELTPAETTVRLRAYMNTPGLPVCQDHLGFWEGVFTGLT
Q913D4  RPSGMFDSVVLCECYDAGCAWYELTPAETTVRLRAYLNTPGLPVCQDHLEFWESVFTGLT
O92531  RPSGIFDTVVLCEAYDTGCAWYELTPSETTVRLRAYLNTPGLPVCQDHLEFWEGVFTGLT
O92529  RPSGMFDSVVLCEAYDTGCAWYELTPAETTVRLRAYLNTPGLPVCQDHLEFWEGVFTGLT
Q68798  RPSGMFDTVVLCEAYDTGAAWYELTPAETTVRLRAYLNTPGLPVCQDHLEFWEAVFTGLT
Q5I2N3  RPSGMFDTVVLCEAYDTGCAWYELTPSETTVRLRAYMNTPGLPVCQDHLEFWEGVFTGLT
O92530  RPSGMFDTVVLCEAYDVGCAWYELTPSETTVRLRAYLNTPGLPVCQDHLEFWEGVFTGMT
O91936  RPSGIFDSVVLCECYDAGCAWYDLTPAETTVRLRAYLNTPGLPVCQDHLEFWEGVFTGLT
O92532  RPSGMFDSVILCEAYDTGCAWYELTPAETTVRLRAYLNTPGLPVCQDHLEFWEGVFTGLT
O39927  RPSGMFDTVVLCEAYDTGCAWYELTPSETTVRLRAYMNTPGLPVCQDHLEFWEGVFTGLT
O39929  RPSGMFDTAELCECYDAGCAWYELTPAETTTRLKAYFDTPGLPVCQDHLEFWESVFTGLT
O39928  RPSGIFDSVVLCECYDAGCAWYDLTPAETTVRLRAYLNTPGLPVCQEHLEFWEGVFTGLT
Q9QAX1  RASGMFDSVVLCECYDAGAAWYELTPAETTVRLRAYFNTPGLPVCQDHLEFWEAVFTGLT
P26660  RASGMFDSVVLCECYDAGAAWYELTPAETTVRLRAYFNTPGLPVCQDHLEFWEAVFTGLT
Q9DHD6  RPSGMFDSVVLCECYDAGAAWYELTPAETTVRLRAYFNTPGLPVCQDHLEFWEAVFTGLT
Q68801  RPSGMFDSVVLCECYDAGCAWYELTPSETTVRLRAYLSTPGLPVCQDHLEFWEGVFTGLT
Q81258  RPSGMFDSVVLCECYDAGCSWYDLQPAETTVRLRAYLSTPGLPVCQDHLDFWESVFTGLT
Q81495  RPSGMFDSVVLCECYDAGCSWYDLQPAETTVRLRAYLSTPGLPVCQDHLDLWESVFTGLT
P26661  RPSGMFDSVVLCECYDAGAAWYELTPAETTVRLRAYFNTPGLPVCQDHLEFWEAVFTGLT
Q99IB8  RASGMFDSVVLCECYDAGAAWYDLTPAETTVRLRAYFNTPGLPVCQDHLEFWEAVFTGLT
Q68749  RASGMFDTVVLCECYDAGAAWYELTPAETTVRLRAYFNTPGLPVCQDHLEFWEAVFTGLT
Q81487  RPSGMFDSVVLCECYDAGCSWYDLQPAETTVRLRAYLSTPGLPVCQDHLDFWERVFTGLT
Q69422  TPSGMVPECNIVEAFDAAKAWYGLSSTEAQTILDTYRTQPGLPAIGANLDEWADLFS.MV
```

```
                550       560       570       580       590       600
NS3     HIDAHFLSQTKQAGDNFPYLVAYQATVCARASAPPPSWDQMWKCLIRLKPTLRGPTPLLY
Q00269  HIDAHFLSQTKQAGDNFPYLVAYQATVCARAQAPPPSWDQMWKCLIRLKPTLHGPTPLLY
P29846  HIDAHFLSQTKQAGDNFPYLVAYQATVCARAQAPPPSWDQMWKCLTRLKPTLHGPTPLLY
P26663  HIDAHFLSQTKQAGDNFPYLVAYQATVCARAQAPPPSWDQMWKCLIRLKPTLHGPTPLLY
Q9WMX2  HIDAHFLSQTKQAGDNFPYLVAYQATVCARAQAPPPSWDQMWKCLIRLKPTLHGPTPLLY
P26662  HIDAHFLSQTKQAGDNLPYLVAYQATVCARAQAPPPSWDQMWKCLIRLKPTLHGPTPLLY
P26664  HIDAHFLSQTKQSGENLPYLVAYQATVCARAQAPPPSWDQMWKCLIRLKPTLHGPTPLLY
Q81754  HIDAHFLSQTKQSGENFPYLVAYQATVCARAKAPPPSWDQMWKCLIRLKPTLTGATPLLY
Q913V3  HIDAHFLSQTKQAGDNFPYLVAYQATVCARAQAPPPSWDQMWKCLIRLKPTLHGPTPLLY
Q03463  HIDAHFLSQTKQGGENFPYLVAYQATVCARAQAPPPSWDQMWKCLIRLKPTLHGPTPLLY
O9297   HIDAHFLSQTKQAGDNFPYLVAYQATVCARAQAPPPSWDQMWKCLIRLKPTLHGPTPLLY
P27958  HIDAHFLSQTKQSGENFPYLVAYQATVCARAQAPPPSWDQMRKCLIRLKPTLHGPTPLLY
Q913D4  HIDAHFLSQTKQSGENFPYLVAYQATVCARARAPPPSWDQMWKCLIRLKPTLTGATPLLY
O92531  HIDAHLLSQTKQGGENFAYLVAYQATVCARAKAPPPSWDTMWKCLIRLKPMLTGPTPLLY
O92529  HIDAHFLSQTKQGGENFAYLVAYQATVCARAKAPPPSWDVMWKCLTRLKPTLTGPTPLLY
Q68798  HIDAHFLSQTKQGGENFAYLVAYQATVCARAKAPPPSWDTMWKCLLRLKPTLTGPTPLLY
Q5I2N3  HIDAHFLSQTKQGGENFAYLVAYQATVCARAKAPPPSWDTMWKCLIRLKPTLTGPTPLLY
O92530  HIDAHFLSQTKQGGENFAYLVAYQATVCARAKAPPPSWDTMWKCLIRLKPMLTGPTPLLY
O91936  NIDAHMLSQTKQGGENFPYLVAYQATVCVRAKAPPPSWDTMWKCMLRLKPTLTGPTPLLY
O92532  HIDAHFLSQTKQAEENFAYLVAYQATVCARAKAPPPSWDTMWKCLIRLKPMLTGPTPLLY
O39927  HIDAHFLSHTKQAGENFAYLVAYQATVCARAKAPPPSWDMMWKCLIRLKPTLTGPTPLLY
O39929  HIDGHFLSQTKQSGENFPYLVAYQATVSAKVWLAPPSWDTMWKCLIRLKPTLHGPTPLLY
O39928  NIDAHMLSQAKQGGENFPYLVAYQATVCVRAKAPPPSWDTMWKCMICLKPTLTGPTPLLY
Q9QAX1  HIDAHFLSQTKQAGENFPYLVAYQATVCARAKAPPPSWDVMWKCLIRLKPTLTGPTPLLY
P26660  HIDAHFLSQTKQSGENFAYLTAYQATVCARAKAPPPSWDVMWKCLTRLKPTLVGPTPLLY
Q9DHD6  HIDAHFLSQTKQGGDNFAYLTAYQATVCARAKAPPPSWDVMWKCLTRLKPTLTGPTPLLY
Q68801  HIDAHFLSQTKQQGLNFPYLTAYQATVCARAAALPPSWDETWKCLIRLKPTLHGPTPLLY
Q81258  HIDAHFLSQTKQQGLNFSYLTAYQATVCARAQAPPPSWDEMWKCLVRLKPTLHGPTPLLY
Q81495  HIDAHFLSQTKQAGLNFSYLTAYQATVCARAQAPPPSWDETWKCLVRLKPTLHGPTPLLY
P26661  HIDAHFLSQTKQGGENFAYLTAYQATVCARAKAPPPSWDVMWKCLTRLKPTLTGPTPLLY
Q99IB8  HIDAHFLSQTKQAGENFAYLVAYQATVCARAKAPPPSWDAMWKCLARLKPTLAGPTPLLY
Q68749  HIDAHFLSQTKQAGEGFPYLVAYQATVCARAKAPPPSWDVMWKCLIRLKPTLVGPTPLLY
Q81487  HIDAHFLSQAKQQGLNFAYLVAYQATVCARAKASPPCWDEMWKCLIRLKPTLQGPTPLLY
Q69422  NPEPSFVNTAKRTADNYVLLTAAQLQLCHQYGYAAPNDAPRWQG.ARLGKK...PCGVLW
```

```
                  610       620       630
                    .         .         .
NS3      RLGSVQNEVTLTHPITKYIMACMSADLEVVT
Q00269   RLGAVQNEITLTHPITKFIMACMSADLEVVT
P29846   RLGAVQNEVTLTHPITKYIMACMSADLEVVT
P26663   RLGAVQNEVTLTHPITKYIMACMSADLEVVT
Q9WMX2   RLGAVQNEVTTTHPITKYIMACMSADLEVVT
P26662   RLGAVQNEVTLTHPITKYIMACMSADLEVVT
P26664   RLGAVQNEITLTHPVTKYIMTCMSADLEVVT
Q81754   RLGGVQNEITLTHPITKYIMACMSADLEVVT
Q913V3   RLGAVQNEITLTHPMTKFIMACMSADLEVVT
Q03463   RLGAVQGEVTLTHPVTKYIMTCMSADLEVVT
O9297    RLGAVQNEVILTHPITKYIMACMSADLEVVT
P27958   RLGAVQNEVTLTHPITKYIMTCMSADLEVVT
Q913D4   RLGSVQNEITLTHPITQYIMACMSADLEVVT
O92531   RLGAVQNEITTTHPITKYIMTCMSADLEVIT
O92529   RLGAVQNEIVTTHPITKYIMTCMSADLEVIT
Q68798   RLGAVQNEVTPTHPVTKYIMACMSADLEVIT
Q5I2N3   RLGAVQNEIITTHPITKYIMTCMSADLEVIT
O92530   RLGAVQNEIITTHPITKYIMTCMAADLEVIT
O91936   RLGAVQNEITLTHPITKYIMACMSADLEVIT
O92532   RLGPVQNEVVTTHPITKYIMTCMSADLEVIT
O39927   RLGAVQNGVITTHPITKYIMTCMSADLEVIT
O39929   RLGSVQNEVVLTHPITKYIMACMSADLEVVT
O39928   RLGAVQNEITLTHPITKYIMACMSADLEVIT
Q9QAX1   RLGPVTNETTLTHPVTKYIATCMQADLEIMT
P26660   RLGSVTNEVTLTHPVTKYIATCMQADLEVMT
Q9DHD6   RLGAVTNEITLTHPVTKYIATCMQADLEVMT
Q68801   RLGAVQNEICTTHPVTKYIATCMAADLEVAT
Q81258   RLGPVQNETCLTHPITKYLMACMSADLEVTT
Q81495   RLGPVQNEICLTHPITKYVMACMSADLEVTT
P26661   RLGAVTNEVTLTHPVTKYIATCMQADLEIMT
Q99IB8   RLGPITNEVTLTHPGTKYIATCMQADLEVMT
Q68749   RLGSVTNEVTLTHPVTKYIATCMQADLEIMT
Q81487   RLGAIQNDICMTHPITKYIMACMSADLEVTT
Q69422   RL.............................
```
