# Supplementary material for: Design of a novel multi-epitope vaccine candidate against hepatitis C virus using structural and nonstructural proteins: An immunoinformatics approach
Source: PLoS One. 2022 Aug 30;17(8):e0272582. doi: 10.1371/journal.pone.0272582 (PMC9426923; doi:10.1371/journal.pone.0272582)
Supplement: S2 Data — (PDF) [file pone.0272582.s015.pdf]

```
           1        10        20        30        40        50        60
p7       ALENLVVLNAASLAGTHGLLSFLVFFCAAWYIKGRLVPGMTYAIYGTWPLLLLLLALPQR
P27958   ALENLVILNAASLAGTHGLVSFLVFFCFAWYLKGRWVPGAVYALYGMWPLLLLLLALPQR
Q03463   ALENLVILNAASLAGTRGLVSFLVFFCFAWYLKGRWVPGAAYALYGMWPLLLLLLALPQR
P26664   ALENLVILNAASLAGTHGLVSFLVFFCFAWYLKGKWVPGAVYTFYGMWPLLLLLLALPQR
Q913D4   ALENLIVLNAASLAGTHGIVPFFIFFCAAWYLKGKWAPGLVYSVYGMWPLLLLLLALPQR
P26663   ALENLVVLNSASVAGAHGILSFLVFFCAAWYIKGRLVPGATYALYGVWPLLLLLLALPPR
Q5EG65   ALENLVVLNAASLAGTHGLVSFLVFFCFAWFLRGKWVPGAVYALYGMWPLLLLLLALPQR
Q81754   ALENLIVLNAASLVGTHGIVPFFIFFCAAWYLKGKWAPGLAYSVYGMWPLLLLLLALPQR
Q9WMX2   ALENLVVLNAASVAGAHGILSFLVFFCAAWYIKGRLVPGAAYALYGVWPLLLLLLALPPR
P26662   .LENLVVLNAASVAGAHGLLSFLVFFCAAWYIKGRLVPGAAYALYGVWPLLLLLLALPPR
Q913V3   ALENLVVLNAASVAGAHGILSFLVFFCAAWYIKGKLVPGAAYAFYGVWPLLLLLLALPPR
O92972   ALENLVVLNAASVAGAHGILSFLVFFCAAWYIKGRLAPGAAYAFYGVWPLLLLLLALPPR
Q00269   ALENLVVLNAASLAGADGILSFLVFFCAAWYIKGRLVPGAAYALYGVWPLLLLLLALPPR
P29846   ALENLVVFNAASVAGMHGTLSFLVFFCAAWYIKGRLVPGAAYALYGVWPLLLLLLALPPR
O92529   ALERLVVLNAASAAGTAGWCWTLIFLCCVWHVKGRLVPACTYTALGMWPILLVILALPQR
Q99IB8   ALEKLVVLHAASAANCHGLLYFAIFFVAAWHIRGRVVPLTTYCLTGLWPFCLLLMALPRQ
Q5I2N3   AVERLVVLNAASAAGTAGWWWAVLFLCCVWYVKGRLVPACTYMALGMWPLLLTILALPHR
Q9DHD6   ALEKLIILHSASAASANGPLWFFIFFTAAWYLKGRVVPAATYSVLGLWSFLLLVLALPQQ
O39927   AVERLVVLNAASAAGTAGWWWAVLFLCCVWYVKGRLVPACTYMALGMWPLLLTILALPPR
P26661   ALEKLIILHSASAASANGPLWFFIFFTAAWYLKGRVVPVATYSVLGLWSFLLLVLALPQQ
Q68749   ALEKLVILHAASAASSNGLLYFILFFVAAWCIKGRAVPMVTYTLLGCWSFVLLLMALPHQ
O92530   ALENLIVLNAASAASSQGWIYCLVFICCAWYIKGRVVPGATYAILHLWPLLLLVLALPQR
Q9QAX1   ALEKLVILHAASAASSHGMLCFIIFFIAAWYIKGRVTPLVTYSYLGMWSFSLLLLALPQQ
Q81258   ALENLVTLNAVAAAGTHGIGWYLVAFCAAWYVRGKLVPLVTYSLTGLWSLALLVLLLPQR
Q81495   ALENLVTLNAVAAAGTHGIGWYLVAFCAAWYVRGKLVPLVTYSLTGLWSLALLVLLLPQR
O91936   ALENVIVLNAAAAAGTHGFFWGLLVICFAWHFKGRLVPGATYLCLGIWPLLLLLFLLPQR
P26660   ALEKLVVLHAASAASCNGFLYFVIFFVAAWYIKGRVVPLATYSLTGLWSFGLLLLALPQQ
Q81487   AMENLVMLNALSAAGQQGYVWYLVAFCAAWHIRGKLVPLITYGLTGLWPLALLDLLLPQR
Q68798   ALENLIVLNAASAAATRGWECFLLFMCWAWYVRGRVVPAVTYGLLNLWPLLLLVLLLPHR
O92531   ALENLIVLNATSAAGSQGWVWGVVFICAAWYIRGRAAPITTYAILQLWPLLLLVLALPRR
Q68801   ALENLIVLNAISAAGTHGIWWSLVAFCVAWHVRGRIFPIAVYSIVGLWPLLLLVLMLPYR
O92532   ALENVIVLNAASAASCQGLLWGLIFICCAWHVRGRAVPVTTYALLQLWPLLLLILALPRR
O39929   ALSNLININAASAAGAQGFWYAILFICIVWHVKGRFPAAAAYAACGLWPCFLLLLMLPER

p7       AYA
P27958   AYA
Q03463   AYA
P26664   AYA
Q913D4   AYA
P26663   AYA
Q5EG65   AYA
Q81754   AYA
Q9WMX2   AYA
P26662   AYA
Q913V3   AYA
O92972   AYA
Q00269   AYA
P29846   AYA
O92529   AYA
Q99IB8   AYA
Q5I2N3   AYA
Q9DHD6   AYA
O39927   AYA
P26661   AYA
Q68749   AYA
O92530   AYA
Q9QAX1   AYA
Q81258   AYA
Q81495   AYA
O91936   ALA
P26660   AYA
Q81487   AYA
Q68798   AYA
O92531   AYA
Q68801   AYA
O92532   AYA
O39929   AYA
```
