# Supplementary material for: Design of a novel multi-epitope vaccine candidate against hepatitis C virus using structural and nonstructural proteins: An immunoinformatics approach
Source: PLoS One. 2022 Aug 30;17(8):e0272582. doi: 10.1371/journal.pone.0272582 (PMC9426923; doi:10.1371/journal.pone.0272582)
Supplement: S3 Data — (PDF) [file pone.0272582.s016.pdf]

```
              1      10        20        30        40        50        60
A0A1B2FV88    SMSYTWTGALITPCAAEESKLPINALSNSLLRHHNMVYATTSRSASQRQKKVTFDRQQVL
Q00269        SMSYTWTGALITPCAAEESKLPINALSNSLLRHHNMVYATTSRSASLRQKKVTFDRLQVL
P26663        SMSYTWTGALITPCAAEESKLPINALSNSLLRHHNMVYATTSRSAGLRQKKVTFDRLQVL
P29846        SMSYTWTGALITPCAAEESKLPINALSNSLLRHHNMVYATTSRSASQRQKKVTIDRLQVL
Q913V3        SMSYTWTGALITPCAAEESKLPINPLSNSLLRHHNMVYATTSRSAGLRQKKVTFDRLQVL
Q9WMX2        SMSYTWTGALITPCAAEETKLPINALSNSLLRHHNLVYATTSRSASLRQKKVTFDRLQVL
O92972        SMSYTWTGALITPCAAEESKLPINPLSNSLLRHHNMVYATTSRSASLRQKKVTFDRLQVL
P26662        SMSYTWTGALITPCAAEESKLPINPLSNSLLRHHSMVYSTTSRSASLRQKKVTFDRLQVL
Q81754        SMSYSWTGALITPCAAEETKLPINALSNSLLRHHNLVYSTTSRSAGQRQKKVTFDRLQVL
Q03463        SMSYTWTGALITPCAAEEQKLPINALSNSLLRHHNLVYSTTSRSACQRQKKVTFDRLQVL
P26664        SMSYSWTGALVTPCAAEEQKLPINALSNSLLRHHNLVYSTTSRSACQRQKKVTFDRLQVL
P27958        SMSYSWTGALVTPCAAEEQKLPINALSNSLLRHHNLVYSTTSRSACQRKKKVTFDRLQVL
Q913D4        SMSYSWTGALITPCASEEAKLPINALSNSLLRHHNLVYSTTSRSAGQRQKKVTFDRVQVL
Q5I2N3        SMSYSWTGALITPCAAEEEKLPINPLSNSLIRHHNMVYSTTSRSASLRQKKVTFDRLQVF
O39927        SMSYSWTGALITPCAAEEEKLPINPLSNSLVRHHNMVYSTTSRSASLRQKKVTFDRVQVF
O92530        SMSYSWTGALITPCAAEEEKLPISPLSNALIRHHNLVYSTTSRSASLRQKKVTFDRVQVV
O92529        SMSYTWTGALITPCAAEEEKLPINPLSNSLIRHHNMVYSTTSRSAGLRQKKVTFDRLQVV
O91936        SMSYSWTGALITPCSAEEEKLPINPLSNTLLRHHNLVYSTSSRSAGQRQKKVTFDRLQVL
Q68798        SMSYSWTGALITPCAAEEEKLPISPLSNTLIRHHNMVYSTTSRSAALRQKKVTFDRQQVV
O39928        SMSYTWTGALITPCSAEEEKLPINPLSNTLLRHHNLVYSTSSRSAGLRQKKVTFDRLQVL
O92531        SMSYSWTGALVTPCAAEEEKLPINPLSNSLIRHHNLVYSTTTRSAAMRQKKVTFDRLQIL
Q68801        SMSYSWTGALITPCAAEEEKLPISPLSNSLLRHHNLVYSTSSRSAAQRQKKVTFDRLQVL
Q9QAX1        SMSYSWTGALITPCGPEEEKLPINPLSNSLLRYHNKVYSTTSRSASQRAKKVTFDRVQLL
O39929        SMSYSWTGALVTPCAAEESKLPISPLSNSLLRHHNMVYATTTRSAVTRQKKVTFDRLQVV
O92532        SYSYSWTGALVTPCAAEEEKLPINPLSNSLIRHHNLVYSTSSRSAATRQKKVTFDRVQLL
Q68749        SMSYSWTGALITPCSPEEEKLPINPLSNSLLRYHNKVYCTTSRSASQRAKKVTFDRVQLL
Q81487        SMSYSWTGAIITPCSAEEEKLPISPLSNSLLRHHNLVYSTSSRSAAARQKKVTFDRLQVL
P26660        SMSYSWTGALITPCSPEEEKLPINPLSNSLLRYHNKVYCTTSKSASLRAKKVTFDRMQAL
P26661        SMSYSWTGALITPCGPEEEKLPINPLSNSLMRFHNKVYSTTSRSASLRAKKVTFDRVQVL
Q99IB8        SMSYSWTGALITPCSPEEEKLPINPLSNSLLRYHNKVYCTTSKSASQRAKKVTFDRTQVL
Q9DHD6        SMSYSWTGALITPCGPEEEKLPISPLSNSLMRFHNKVYSTTSRSASLRAKKVTFDRVQVL
Q81495        SMSYSWTGALITPCSAEEEKLPISPLSNSLLRHHNLVYSTSSRSASQRQKKVTFDRLQVL
Q81258        SMSYSWTGALITPCSAEEEKLPISPLSNSLLRHHNLVYSTSSRSASQRQKKVTFDRLQVL
Q69422        SMSYTWTD.VISFKTASKVLSATRAITSGFLKQRSLVYVTEPRDAELRKQKVTINRQPLF

                       70        80        90        100       110
A0A1B2FV88    DDHYRDVLKEMKAKASTVKAKLLSVEEACKLTPPHSARSKF.GYGAKDVRN.LSSKAVNH
Q00269        DDHYRDVLKEMKAKASTVKAKLLSVEEACKLTPPHSAKSKF.GYGAKDVRN.LSSKAINH
P26663        DDHYRDVLKEMKAKASTVKAKLLSVEEACKLTPPHSAKSKF.GYGAKDVRN.LSSKAVNH
P29846        DDHYRDVLKEMKAKASTVKAKLLSVEEACKLTPPHSARSKF.GYGAKDVRN.LSGKAINH
Q913V3        DDHYRDVLKEMKAKASTVKAKLLSIEEACKLTPPHSARSKF.GYGAKDVRN.LSSKAVNH
Q9WMX2        DDHYRDVLKEMKAKASTVKAKLLSVEEACKLTPPHSARSKF.GYGAKDVRN.LSSKAVNH
O92972        DDHYRDVLKEMKAKASTVKAKLLSIEEACKLTPPHSAKSKF.GYGAKDVRN.LSSRAVNH
P26662        DDHYRDVLKEMKAKASTVKARLLSIEEACKLTPPHSAKSKF.GYGAKDVRS.LSSRAVNH
Q81754        DDHYRDVLKEAKAKASTVKAKLLSVEEACSLTPPHSARSKF.GYGAKDVRS.HSSKAIRH
Q03463        DSHYQDVLKEVKAAASKVKANLLSVEEACSLTPPHSAKSKF.GYGAKDVRC.HARKAVNH
P26664        DSHYQDVLKEVKAAASKVKANLLSVEEACSLTPPHSAKSKF.GYGAKDVRC.HARKAVTH
P27958        DSHYQDVLKEVKAAASKVKANLLSVEEACSLAPPHSAKSKF.GYGAKDVRC.HARKAVAH
Q913D4        DDHYRDVLKEAKAKASTVKARLLSVEEACSLTPPHSARSKF.GYGAKDVRS.HSSKAIRH
Q5I2N3        DQHYQDVLKEIKLRASTVQARLLSIEEACDLTPSHSARSKY.GYGAQDVRS.HASKAINH
O39927        DQHYQDVLKEIKLRASTVQAKLLSIEEACDLTPSHSARSKY.GYGAQDVRS.RASKAVDH
O92530        DQHYYDVLKEIKTKASGVSAKLLSVEEACALTPPHSARSKF.GYGAKEVRG.LASKAVNH
O92529        DQHYQDVLKEIKLRASTVHARLLSTEEACSLTPPHSARSRY.GYGARDVRS.HTSKAVKH
O91936        DDHYREVVDEMKRLASKVKARLLPLEEACGLTPPHSARSKY.GYGAKEVRS.LDKKALNH
Q68798        DQHYYDTLKEMKARASTVSAKLLSVEEACDLTPAHSARSKF.GYGAKDVRG.RTSKALNH
O39928        DDHYREVVDEMKRLASKVKARLLPLEEACGLTPPHSARSKY.GYGAKEVRS.LDKKALKH
O92531        DQHYNNVVKEVKLRASGVTAKLLSVEEACSLTPPHSARSKF.GYGAKDVRS.HTSKAINH
Q68801        DDHYNTTLKEIKELASGVKAELLSVEEACRLVPSHSARSKF.GYGAKEVRS.LSSKAINH
Q9QAX1        DSHYDQVLKDIKLAASKVSANLLSIEEACALTPPHSARSKY.GFGAKEVRS.LSRKAVDH
O39929        DSTYNEVLKEIKARASRVKPRLLTTEEACDLTPPHSARSKF.GYGKKDVRS.HSRKAINH
O92532        DQHYYDTVKEIKLRASHVKAQLLSTEEACDLTPPHSARSKF.GYGAKDVRS.HASKAINH
Q68749        DSHYESVLKDVKQAATKVSAKLLSIEEACALTPPHSARSKY.GFGAKEVRS.LSRRAVDH
Q81487        DDHYKNVLKEVKERASGVKGRLLSFEEACSLVPPHSGRSKY.GYSAKDVRS.LSSKAMNQ
P26660        DAHYDSVLKDIKLAASKVTARLLTLEEACQLTPPHSARSKY.GFGAKEVRS.LSGRAVNH
P26661        DAHYDSVLQDVKRAASKVSARLLTVEEACALTPPHSAKSRY.GFGAKEVRS.LSRRAVNH
Q99IB8        DAHYDSVLKDIKLAASKVSARLLTLEEACQLTPPHSARSKY.GFGAKEVRS.LSGRAVNH
Q9DHD6        DAHYDSVLQDVKRAASKVSARLLSVEEACALTPPHSAKSRY.GFGAKEVRS.LSRGAVNH
Q81495        DDHYKTALQEVKERASRVKARMLSIEEACALVPPHSARSKF.GYSAKDVRS.LSSKAINQ
Q81258        DDHYKTALKEVKERASRVKARMLTIEEACALVPPHSARSKF.GYSAKDVRS.LSSRAINQ
Q69422        PPSYHKQVRLAKEKASKVVGVMWDYDEVAAHTPSKSAKSHITGLRGTDVRSGAARKAVLD
```

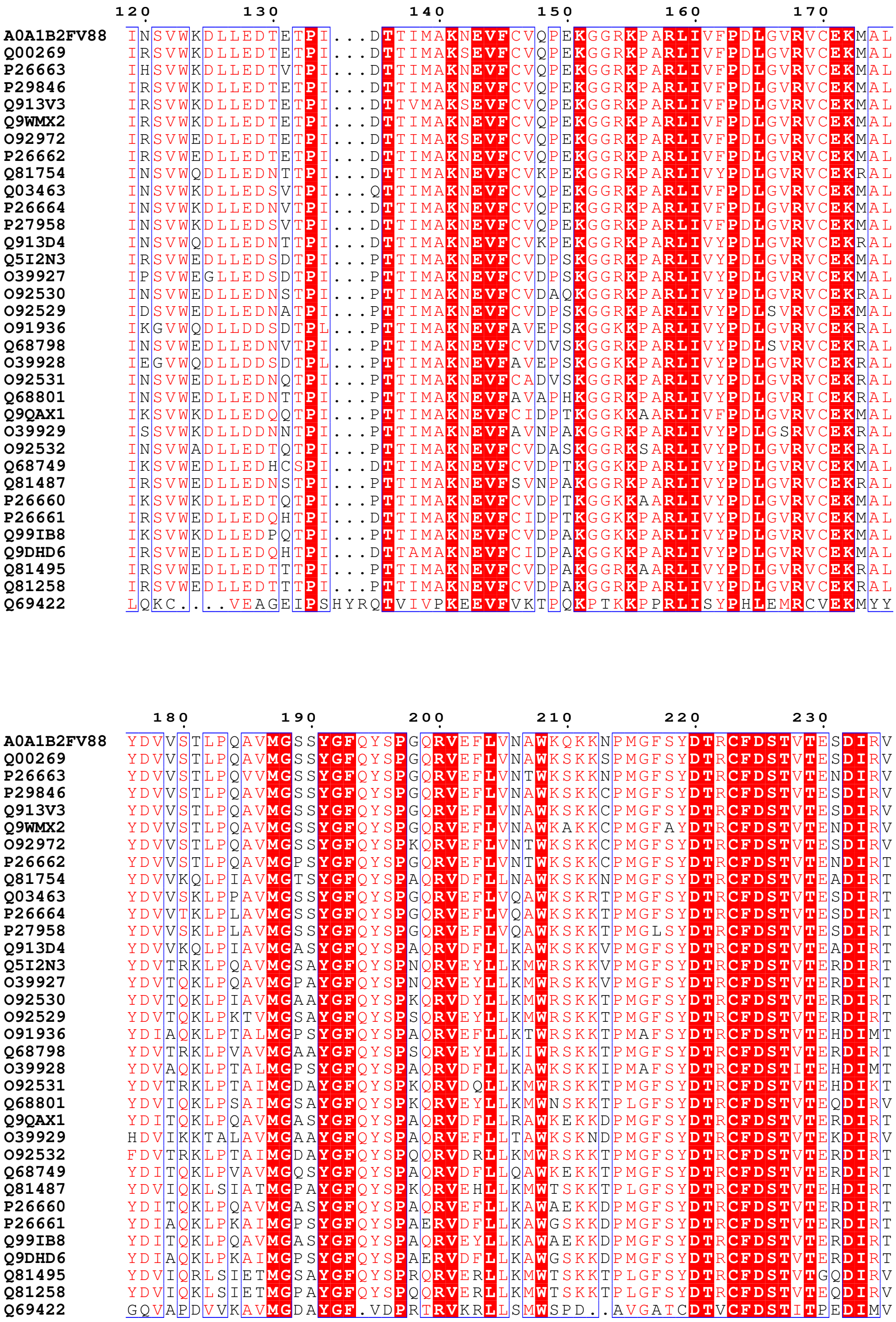
120 130 140 150 160 170
A0A1B2FV88 INSVWKDLLEDTETPI...DTTIMAKNEVFCVQPEKGGRKPARLIVFPDLGVRVCEKMAL
Q00269 IRSVWKDLLEDTETPI...DTTIMAKSEVFCVQPEKGGRKPARLIVFPDLGVRVCEKMAL
P26663 IHSVWKDLLEDTVTPI...DTTIMAKNEVFCVQPEKGGRKPARLIVFPDLGVRVCEKMAL
P29846 IRSVWKDLLEDTETPI...DTTIMAKNEVFCVQPEKGGRKPARLIVFPDLGVRVCEKMAL
Q913V3 IRSVWKDLLEDTETPI...DTTVMAKSEVFCVQPEKGGRKPARLIVFPDLGVRVCEKMAL
Q9WMX2 IRSVWKDLLEDTETPI...DTTIMAKNEVFCVQPEKGGRKPARLIVFPDLGVRVCEKMAL
O92972 IRSVWEDLLEDTETPI...DTTIMAKSEVFCVQPEKGGRKPARLIVFPDLGVRVCEKMAL
P26662 IRSVWEDLLEDTETPI...DTTIMAKNEVFCVQPEKGGRKPARLIVFPDLGVRVCEKMAL
Q81754 INSVWQDLLEDNTTPI...DTTIMAKNEVFCVKPEKGGRKPARLIVYPDLGVRVCEKRAL
Q03463 INSVWKDLLEDSVTPI...QTTIMAKNEVFCVQPEKGGRKPARLIVFPDLGVRVCEKMAL
P26664 INSVWKDLLEDNVTPI...DTTIMAKNEVFCVQPEKGGRKPARLIVFPDLGVRVCEKMAL
P27958 INSVWKDLLEDSVTPI...DTTIMAKNEVFCVQPEKGGRKPARLIVFPDLGVRVCEKMAL
Q913D4 INSVWQDLLEDNTTPI...DTTIMAKNEVFCVKPEKGGRKPARLIVYPDLGVRVCEKRAL
Q5I2N3 IRSVWEDLLEDSDTPI...PTTIMAKNEVFCVDPSKGGRKPARLIVYPDLGVRVCEKMAL
O39927 IPSVWEGLLEDSDTPI...PTTIMAKNEVFCVDPSKGGRKPARLIVYPDLGVRVCEKMAL
O92530 INSVWEDLLEDNSTPI...PTTIMAKNEVFCVDAQKGGRKPARLIVYPDLGVRVCEKRAL
O92529 IDSVWEDLLEDNATPI...PTTIMAKNEVFCVDPSKGGRKPARLIVYPDLSVRVCEKMAL
O91936 IKGVWQDLLDDSDTPL...PTTIMAKNEVFAVEPSKGGKKPARLIVYPDLGVRVCEKRAL
Q68798 INSVWEDLLEDNVTPI...PTTIMAKNEVFCVDVSKGGRKPARLIVYPDLSVRVCEKRAL
O39928 IEGVWQDLLDDSDTPL...PTTIMAKNEVFAVEPSKGGKKPARLIVYPDLGVRVCEKRAL
O92531 INSVWEDLLEDNQTPI...PTTIMAKNEVFCADVSKGGRKPARLIVYPDLGVRVCEKRAL
Q68801 INSVWEDLLEDNTTPI...PTTIMAKNEVFAVAPHKGGRKPARLIVYPDLGVRICEKRAL
Q9QAX1 IKSVWKDLLEDQQTPI...PTTIMAKNEVFCIDPTKGGKKAARLIVFPDLGVRVCEKMAL
O39929 ISSVWKDLLDDNNTPI...PTTIMAKNEVFAVNPAKGGRKPARLIVYPDLGSRVCEKRAL
O92532 INSVWADLLEDTQTPI...PTTIMAKNEVFCVDASKGGRKSARLIVYPDLGVRVCEKRAL
Q68749 IKSVWEDLLEDHCSPI...DTTIMAKNEVFCVDPTKGGKKPARLIVYPDLGVRVCEKMAL
Q81487 IRSVWEDLLEDNSTPI...PTTIMAKNEVFSVNPAKGGRKPARLIVYPDLGVRVCEKRAL
P26660 IKSVWKDLLEDTQTPI...PTTIMAKNEVFCVDPTKGGKKAARLIVYPDLGVRVCEKMAL
P26661 IRSVWEDLLEDQHTPI...DTTIMAKNEVFCIDPTKGGKKPARLIVYPDLGVRVCEKMAL
Q99IB8 IKSVWKDLLEDPQTPI...PTTIMAKNEVFCVDPAKGGKKPARLIVYPDLGVRVCEKMAL
Q9DHD6 IRSVWEDLLEDQHTPI...DTTAMAKNEVFCIDPAKGGKKPARLIVYPDLGVRVCEKMAL
Q81495 IRSVWEDLLEDTTTPI...PTTIMAKNEVFCVDPAKGGRKAARLIVYPDLGVRVCEKRAL
Q81258 IRSVWEDLLEDTTTPI...PTTIMAKNEVFCVDPAKGGRKPARLIVYPDLGVRVCEKRAL
Q69422 LQKC....VEAGEIPSHYRQTVIVPKEEVFVKTPQKPTKKPPRLISYPHLEMRCVEKMYY
180 190 200 210 220 230
A0A1B2FV88 YDVVSTLPQAVMGSSYGFQYSPGQRVEFLVNAWKQKKNPMGFSYDTRCFDSTVTESDIRV
Q00269 YDVVSTLPQAVMGSSYGFQYSPGQRVEFLVNAWKSKKSPMGFSYDTRCFDSTVTESDIRV
P26663 YDVVSTLPQVVMGSSYGFQYSPGQRVEFLVNTWKSKKNPMGFSYDTRCFDSTVTENDIRV
P29846 YDVVSTLPQAVMGSSYGFQYSPKQRVEFLVNAWKSKKCPMGFSYDTRCFDSTVTESDIRV
Q913V3 YDVVSTLPQAVMGSSYGFQYSPGQRVEFLVNAWKSKKCPMGFSYDTRCFDSTVTESDIRV
Q9WMX2 YDVVSTLPQAVMGSSYGFQYSPGQRVEFLVNAWKAKKCPMGFAYDTRCFDSTVTENDIRV
O92972 YDVVSTLPQAVMGSSYGFQYSPKQRVEFLVNTWKSKKCPMGFSYDTRCFDSTVTESDIRV
P26662 YDVVSTLPQAVMGPSYGFQYSPGQRVEFLVNTWKSKKCPMGFSYDTRCFDSTVTENDIRT
Q81754 YDVVKQLPIAVMGTSYGFQYSPAQRVDFLLNAWKSKKNPMGFSYDTRCFDSTVTEADIRT
Q03463 YDVVSKLPLAVMGSSYGFQYSPGQRVEFLVQAWKSKKTPMGFSYDTRCFDSTVTESDIRT
P26664 YDVVTKLPLAVMGSSYGFQYSPGQRVEFLVQAWKSKKTPMGFSYDTRCFDSTVTESDIRT
P27958 YDVVSKLPLAVMGSSYGFQYSPGQRVEFLVQAWKSKKTPMGLSYDTRCFDSTVTESDIRT
Q913D4 YDVVKQLPIAVMGASYGFQYSPAQRVDFLLKAWKSKKVPMGFSYDTRCFDSTVTEADIRT
Q5I2N3 YDVTRKLPQAVMGSAYGFQYSPNQRVEYLLKMWRSKKVPMGFSYDTRCFDSTVTERDIRT
O39927 YDVTQKLPQAVMGPAYGFQYSPNQRVEYLLKMWRSKKVPMGFSYDTRCFDSTVTERDIRT
O92530 YDVTQKLPIAVMGAAYGFQYSPKQRVDYLLKMWRSKKTPMGFSYDTRCFDSTVTERDIRT
O92529 YDVTQKLPKTVMGSAYGFQYSPSQRVEYLLKMWRSKKTPMGFSYDTRCFDSTVTERDIRT
O91936 YDIAQKLPTALMGPSYGFQYSPAQRVEFLLKTWRSKKTPMAFSYDTRCFDSTVTEHDIMT
Q68798 YDVTRKLPVAVMGAAYGFQYSPSQRVEYLLKIWRSKKTPMGFSYDTRCFDSTVTERDIRT
O39928 YDVAQKLPTALMGPSYGFQYSPAQRVDFLLKAWKSKKIPMAFSYDTRCFDSTITEHDIMT
O92531 YDVTRKLPTAIMGDAYGFQYSPKQRVDQLLKMWRSKKTPMGFSYDTRCFDSTVTEHDIKT
Q68801 YDVIQKLPSAIMGSAYGFQYSPKQRVEYLLKMWNSKKTPLGFSYDTRCFDSTVTEQDIRV
Q9QAX1 YDITQKLPQAVMGASYGFQYSPAQRVDFLLRAWKEKKDPMGFSYDTRCFDSTVTERDIRT
O39929 HDVIKKTALAVMGAAYGFQYSPAQRVEFLLTAWKSKNDPMGFSYDTRCFDSTVTEKDIRV
O92532 FDVTRKLPTAIMGDAYGFQYSPQQRVDRLLKMWRSKKTPMGFSYDTRCFDSTVTERDIRT
Q68749 YDITQKLPVAVMGQSYGFQYSPAQRVDFLLQAWKEKKTPMGFSYDTRCFDSTVTERDIRT
Q81487 YDVIQKLSIATMGPAYGFQYSPKQRVEHLLKMWTSKKTPLGFSYDTRCFDSTVTEHDIRT
P26660 YDITQKLPQAVMGASYGFQYSPAQRVEFLLKAWAEKKDPMGFSYDTRCFDSTVTERDIRT
P26661 YDIAQKLPKAIMGPSYGFQYSPAERVDFLLKAWGSKKDPMGFSYDTRCFDSTVTERDIRT
Q99IB8 YDITQKLPQAVMGASYGFQYSPAQRVEYLLKAWAEKKDPMGFSYDTRCFDSTVTERDIRT
Q9DHD6 YDIAQKLPKAIMGPSYGFQYSPAERVDFLLKAWGSKKDPMGFSYDTRCFDSTVTERDIRT
Q81495 YDVIQRLSIETMGSAYGFQYSPRQRVERLLKMWTSKKTPLGFSYDTRCFDSTVTGQDIRV
Q81258 YDVIQKLSIETMGPAYGFQYSPQQRVERLLKMWTSKKTPLGFSYDTRCFDSTVTEQDIRV
Q69422 GQVAPDVVKAVMGDAYGF.VDPRTRVKRLLSMWSPD..AVGATCDTVCFDSTITPEDIMV

```
                  240       250       260       270       280       290
A0A1B2FV88  EESIYQCCDLAPEARQAIRSLTERLYIGGPLTNSKGQNCGYRRCRASGVLTTSCGNTLTC
Q00269      EESIYQCCDLAPEARQAIKSLTERLYIGGPLTNSKGQNCGYRRCRASGVLTTSCGNTLTC
P26663      EESIYQCCDLAPEARQAIKSLTERLYIGGPLTNSKGQNCGYRRCRASGVLTTSCGNTLTC
P29846      EESIYQCCDLAPEARQAIRSLTERLYIGGPLTNSKGQNCGYRRCRASGVLTTSCGNTLTC
Q913V3      EESIYQCCDLAPEARQAIKSLTERLYIGGPLTNSKGQNCGYRRCRASGVLTTSCGNTLTC
Q9WMX2      EESIYQCCDLAPEARQAIRSLTERLYIGGPLTNSKGQNCGYRRCRASGVLTTSCGNTLTC
O92972      EESIYQCCDLAPEARQAIRSLTERLYIGGPLTNSKGQNCGYRRCRASGVLTTSCGNTLTC
P26662      EESIYQCCDLAPEARQAIRSLTERLYVGGPLTNSKGQNCGYRRCRASGVLTTSCGNTLTC
Q81754      EEDLYQSCDLVPEARAAIRSLTERLYIGGPLTNSKGQNCGYRRCRASGVLTTSCGNTITC
Q03463      EEAIYQCCDLDPQARVAIRSLTERLYVGGPLTNSRGENCGYRRCRASGVLTTSCGNTLTC
P26664      EEAIYQCCDLDPQARVAIKSLTERLYVGGPLTNSRGENCGYRRCRASGVLTTSCGNTLTC
P27958      EEAIYQCCDLDPQARVAIKSLTERLYVGGPLTNSRGENCGYRRCRASRVLTTSCGNTLTR
Q913D4      EEDLYQSCDLAPEARIAIRSLTERLYIGGPLTNSKGQNCGYRRCRASGVLTTSCGNTITC
Q5I2N3      ENDIYQSCQLDPVARRAVSSLTERLYVGGPMVNSKGQSCGYRRCRASGVLPTSMGNTLTC
O39927      ENDIYQSCQLDPVARRVVSSLTERLYVGGPMANSKGQSCGYRRCRASGVLPTSMGNTLTC
O92530      EEDIYQCCQLDPVAKKAITSLTERLYCGGPMYNSRGQSCGYRRCRASGVLTTSLGNTLTC
O92529      EEDIYQSCQLDPTARKAISSLTERLYCGGPMFNSKGESCGYRRCRASGVLTTSLGNTLTC
O91936      EESIYQSCDLQPEARAAIRSLTQRLYCGGPMYNSKGQQCGYRRCRASGVFTTSMGNTMTC
Q68798      EESIYQCCELDPVARKAISSLTERLYVGGPMYNSQGQSCGYRRCRASGVLPTSMGNTLTC
O39928      EESIYQSCDLQPEARVAIRSLTQRLYCGGPMYNSKGQQCGYRRCRASGVFTTSMGNTMTC
O92531      ERDVYLSCKLDPVARKAIESLTERLYIGGPMYNSRGQLCGTRRCRASGVLTTSLGNTMTC
Q68801      EESIYQACDLKDEARRVITSLTERLYCGGPMFNSKGQHCGYRRCRASGVLPTSFGNTVTC
Q9QAX1      EESIYLACSLPEEARVAIHSLTERLYVGGPMMNSKGQSCGYRRCRASGVLTTSMGNTITC
O39929      EEEVYQCCDLEPEARKVITALTDRLYVGGPMHNSKGDLCGYRRCRATGVYTTSFGNTLTC
O92532      EQDIYLSCQLDPEARKVIESLTERLYVGGPMYNSKGQLCGQRRCRASGVLPTSMGNTVTC
Q68749      EESIYLSCSLPEEARTAIHSLTERLYVGGPMTNSKGQSCGYRRCRASGVLTTSMGNTLTC
Q81487      EEGIYQCCDLEPEARKAISALTERLYIGGPMYNSKGLQCGYRRCRASGVLPTSFGNTITC
P26660      EESIYRACSLPEEAHTAIHSLTERLYVGGPMFNSKGQTCGYRRCRASGVLTTSMGNTITC
P26661      EESIYQACSLPQEARTVIHSLTERLYVGGPMTNSKGQSCGYRRCRASGVFTTSMGNTMTC
Q99IB8      EESIYQACSLPEEARTAIHSLTERLYVGGPMFNSKGQTCGYRRCRASGVLTTSMGNTITC
Q9DHD6      EESIYQACSLPQEARTVIHSITERLYVGGPMTNSKGQSCGYRRCRASGVFTTSMGNTMTC
Q81495      EEAVYQCCNLEPEPGQAISSLTERLYCGGPMNNSKGAQCGYLRCRASGVLPTSFGNTITC
Q81258      EEEIYQCCNLEPEARKVISSLTERLYCGGPMFNSKGAQCGYRRCRASGVLPTSFGNTITC
Q69422      ETDIYSAAKLSDQHRAGIHTIARQLYAGGPMIAYDGREIGYRRCRSSGVYTTSSSNSLTC
```

```
                  300       310       320       330       340       350
A0A1B2FV88  YLKASAACRAAKLQDCTMLVCGDDLVVICESAGTQEDAASLRVFTEAMTRYSAPPGDPPQ
Q00269      YLKATAACRAAKLQDCTMLVNGDDLVVICESAGTQEDAASLRVFTEAMTRYSAPPGDPPQ
P26663      YLKASAACRAAKLQDCTMLVNGDDLVVICESAGTQEDAASLRVFTEAMTRYSAPPGDPPQ
P29846      YLKASAACRAAKLQDCTMLVCGDDLVVICESAGTQEDAASLRVFTEAMTRYSAPPGDLPQ
Q913V3      YLKASAACRAAKLRDCTMLVNGDDLVVICESAGTQEDEANLRVFTEAMTRYSAPPGDPPR
Q9WMX2      YLKAAAACRAAKLQDCTMLVCGDDLVVICESAGTQEDEASLRAFTEAMTRYSAPPGDPPK
O92972      YLKATAACRAAKLQDCTMLVNGDDLVVICESAGTQEDAAALRAFTEAMTRYSAPPGDPPQ
P26662      YLKATAACRAAKLQDCTMLVNGDDLVVICESAGTQEDAAALRAFTEAMTRYSAPPGDPPQ
Q81754      YLKASAACRAAKLRDCTMLVCGDDLVVICESAGVQEDAANLRAFTEAMTRYSAPPGDPPQ
Q03463      YIKARAACRAAGLQDCTMLVCGDDLVVICESAGVQEDAASLRAFTEAMTRYSAPPGDPPQ
P26664      YIKARAACRAAGLQDCTMLVCGDDLVVICESAGVQEDAASLRAFTEAMTRYSAPPGDPPQ
P27958      YIKARAACRAAGLQDCTMLVCGDDLVVICESAGVQEDAASLRAFTEAMTRYSAPPGDPPQ
Q913D4      FLKASAACRAAKLQDCTMLVCGDDLVVICESAGVQEDAASLRAFTEAMTRYSAPPGDPPQ
Q5I2N3      YLKAQAACRAANIKDCDMLVCGDDLVVICESAGVQEDTASLRAFTDAMTRYSAPPGDVPQ
O39927      YLKAQAACRAANIKDCDMLVCGDDLVVICESAGVQEDTASLRAFTDAMTRYSAPPGDAPQ
O92530      YLKAQAACRAAKLKDFDMLVCGDDLVVISESMGVAEDASALRAFTEAMTRYSAPPGDDPQ
O92529      YLKAQAACRAANIKNFDMLVCGDDLVVICESAGVQEDVVALRAFTDAMIRYSAPPGDAPQ
O91936      YIKALASCRAAKLRDCTLLVCGDDLVAICESQGTHEDEASLRAFTEAMTRYSAPPGDPPV
Q68798      YLKAMAACKAAGLKNFDMLVCGDDLVVISESLGVSEDASALRAFTDAMTRYSAPPGDEPH
O39928      YIKALASCRAAKLRDCTLLVCGDDLVAICESQGTHEDEASLRAFTEAMTRYSAPPGDPPV
O92531      FIKAEAACRAAGLTNYDMLVCGDDLVVIAESAGVQEDASNLRAFTEAMTRYSAPPGDEPH
Q68801      YLKAKAATKAAGIKDPSFLVCGDDLVVIAESAGIDEDKSALRAFTEAMTRYSAPPGDPPQ
Q9QAX1      YVKALAACKAAGIVAPTMLVCGDDLVVISESQGAEEDERNLRVFTEAMTRYSAPPGDPPK
O39929      YLKATAAIRAAALRDCTMLVCGDDLVVIAESDGVEEDNRALRAFTEAMTRYSAPPGDAPQ
O92532      FLKATAACRAAGFTDYDMLVCGDDLVVVTESAGVNEDIANLRAFTEAMTRYSATPGDEPS
Q68749      YVKAKAACNAAGIVAPTMLVCGDDLVVISESQGVEEDERNLRVFTEAMTRYSAPPGDPPK
Q81487      YIKATAASRAAGLKNPSFLVCGDDLVVISESCGVEEDRTALRAFTEAMTRYSAPPGDAPQ
P26660      YVKALAACKAAGIIAPTMLVCGDDLVVISESQGTEEDERNLRAFTEAMTRYSAPPGDPPR
P26661      YIKALAACKAAGIVDPVMLVCGDDLVVISESQGNEEDERNLRAFTEAMTRYSAPPGDLPR
Q99IB8      YVKALAACKAAGIVAPTMLVCGDDLVVISESQGTEEDERNLRAFTEAMTRYSAPPGDPPR
Q9DHD6      YIKALAACKAAGIVDPTMLVCGDDLVVISESQGNEEDERNLRAFTEAMTRYSAPPGDLPR
Q81495      YIKATAAARAAGLRNPDFLVCGDDLVVVAESDGVDEDRATLRAFTEAMTRYSAPPGDAPQ
Q81258      YIKATAAAKAANLRNPDFLVCGDDLVVVAESDGVDEDRAALRAFTEAMTRYSAPPGDAPQ
Q69422      WLKVNAAAEQAGMKNPRFLICGDDCTVIWKSAGADADKQAMRVFASWMKVMGAPQDCVPQ
```

```
                 360       370       380       390       400       410
A0A1B2FV88  PEYDLERITSCSSNVSVAHDASGKRVYYLTRDPTTPLARAAWETARHTPVNSWLGNIIMF
Q00269      PEYDLELITSCSSNVSVAHDASGKRVYYLTRDPTTPLARAAWETARHTPVNSWLGNIIMY
P26663      PEYDLELITSCSSNVSVAHDASGKRVYYLTRDPTTPLARAAWETARHTPVNSWLGNIIMY
P29846      PEYDQELITSCSSNVSVAHDASGKRVYYLTRDPTTPLARAAWATARHTPVNSWLGNIIMY
Q913V3      PEYDLELITSCSSNVSVAHDASGKRVYYLTRDPSTPLARAAWETARHTPVNSWLGNIIMY
Q9WMX2      PEYDLELITSCSSNVSVAHDASGKRVYYLTRDPTTPLARAAWETARHTPVNSWLGNIIMY
O92972      PEYDLELITSCSSNVSVAHDASGKRVYYLTRDPTTPLARAAWETARHTPINSWLGNIIMY
P26662      PEYDLELITSCSSNVSVAHDASGKRVYYLTRDPTTPLARAAWETVRHTPVNSWLGNIIMY
Q81754      PEYDLELITSCSSNVSVAHDGAGKRVYYLTRDPETPLARAAWETARHTPVNSWLGNIIMF
Q03463      PEYDLELITSCSSNVSVAHDGTGKRVYYLTRDPTTPLARAAWETARHTPVNSWLGNIIMF
P26664      PEYDLELITSCSSNVSVAHDGAGKRVYYLTRDPTTPLARAAWETARHTPVNSWLGNIIMF
P27958      PEYDLELITSCSSNVSVAHDGAGKRVYYLTRDPTTPLARAAWETARHTPVNSWLGNIIMF
Q913D4      PEYDLELITSCSSNVSVARDGAGKRVYYLTRDPETPLARAAWETARHTPVNSWLGNIIMF
Q5I2N3      PTYDLELITSCSSNVSVAHDGNGKRYYYLTRDCTTPLARAAWETARHTPVNSWLGNIIMF
O39927      PTYDLELITSCSSNVSVAHEGNGKKYYYLTRDCTTPLARAAWETARHTPVNSWLGNIIMF
O92530      PEYDLELITSCSSNVSVAHDGAGQRYYYLTRDPLTPLSRAAWETARHTPVNSWLGNIIMY
O92529      PTYDLELITSCSSNVSVAHDGTGQRYYYLTRDCTTPLARAAWETARHTPVNSWLGNIIMY
O91936      PAYDLELVTSCSSNVSVAHDASGNRVYYLTRDPQVPLARAAWETAKHSPVNSWLGNIIMY
Q68798      PEYDLEHITSCSSNVSVAHDHTGQRYYYLTRDPTNVLARAAWETARHTPVNSWLGNIIMY
O39928      PAYDLELVTSCSSNVSVARDASGNRIYYLTRDPQVPLAKAAWETAKHSPVNSWLGNIIMY
O92531      PAYDLELITSCSSNVSVAHDHTGQRYYYLTRDPTTPLSRAAWETARHTPVNSWLGNIIMY
Q68801      PTYDLELITSCSSNVSVAHDGAGKRYYYLTRDPETPLARAAWETARHTPVNSWLGNIIMY
Q9QAX1      PEYDLELITSCSSNVSVALDQHGRRMYYLTRDPSTPLARAAWETARHSPVNSWLGNIIQY
O39929      PAYDLELITSCSSNVSVAHDVTGKKVYYLTRDPETPLARAVWETVRHTPVNSWLGNIIVY
O92532      PTYDLELITSCSSNVSVAHDGDGRRYYYLTRDPVTPLARAAWETARHTPVNSWLGNIIMY
Q68749      AEYDLELITSCSSNVSVALDPRGRRRYYLTRDPTTPLARAAWETARHSPVNSWLGNIIQY
Q81487      PTYDLELISSCSSNVSVACDGAGKRYYYLTRDPETPLARAAWETARHTPVNSWLGNIIMF
P26660      PEYDLELITSCSSNVSVALGPQGRRRYYLTRDPTTPIARAAWETVRHSPVNSWLGNIIQY
P26661      PEYDLELITSCSSNVSVALDSRGRRRYFLTRDPTTPITRAAWETVRHSPVNSWLGNIIQY
Q99IB8      PEYDLELITSCSSNVSVALGPRGRRRYYLTRDPTTPLARAAWETVRHSPINSWLGNIIQY
Q9DHD6      PEYDLELITSCSSNVSVALDSRGRRRYFLTRDPTTPITRAAWETVRHSPVNSWLGNIIQY
Q81495      PTYDLELITSCSSNVSVARDDKGKRYYYLTRDATTPLARAAWETARHTPVNSWLGSIIMY
Q81258      ATYDLELITSCSSNVSVARDDKGRRYYYLTRDATTPLARAAWETARHTPVNSWLGNIIMY
Q69422      PKYSLEELTSCSSNVTSGITKSGKPYYFLTRDPRIPLGRCSAEGLGYNPSAAWIGYLIHH
```

```
                 420       430       440       450       460       470
A0A1B2FV88  APTLWARMVLMTHFFSILLAQEQLEKALDCQIYGACHSVEPLDLPQIIQRLHGLSAFSLH
Q00269      APTLWARMILMTHFFSILLAQEQLEKALDCQIYGACYSIEPLDLPQIIQRLHGLSAFSLH
P26663      APTLWARMILMTHFFSILLAQEQLEKALDCQIYGACYSIEPLDLPQIIERLHGLSAFSLH
P29846      APTLWARMILMTHFFSILLAQEQLEKALDCQIYGACYSIEPLDLPQIIERLHGLSAFSLH
Q913V3      APTLWARMILMTHFFSILLAQEQLEKALDCQIYGACYSIEPLDLPQIIERLHGLSAFSLH
Q9WMX2      APTLWARMILMTHFFSILLAQEQLEKALDCQIYGACYSIEPLDLPQIIQRLHGLSAFSLH
O92972      APTLWARMILMTHFFSILLAQEQLEKALDCQIYGACYSIEPLDLPQIIERLHGLSAFTLH
P26662      APTLWARMILMTHFFSILLAQEQLEKALDCQIYGACYSIEPLDLPQIIERLHGLSAFSLH
Q81754      APTLWVRMVLMTHFFSILIAQEHLEKALDCEIYGAVHSVQPLDLPEIIQRLHGLSAFSLH
Q03463      APTLWARMILMTHFFSVLIARDQLEQALDCEIYGACYSIEPLDLPPIIQRLHGLSAFSLH
P26664      APTLWARMILMTHFFSVLIARDQLEQALDCEIYGACYSIEPLDLPPIIQRLHGLSAFSLH
P27958      APTLWARMILMTHFFSVLIARDQLEQALNCEIYGACYSIEPLDLPPIIQRLHGLSAFSLH
Q913D4      APTLWVRMVLMTHFFSILIAQEHLGKALDCEIYGAVHSVQPLDLPEIIQRLHSLSAFSLH
Q5I2N3      APTIWVRMVLMTHFFSILQSQEQLEKALDFDIYGVTYSVSPLDLPAIIQRLHGMAAFSLH
O39927      APTIWVRMVLMNHFFSILQSQEQLEKAFDFDIYGVTYSVSPLDLPAIIQRLHGMAAFSLH
O92530      APTIWVRMVLMTHFFAILQSQEILHKALDFDMYGVTYSVTPLDLPYIIQRLHGMAAFSLH
O92529      APTIWVRMVLMTHFFSILQCQEQLEAALNFDMYGVTYSVTPLDLPAIIQRLHGMAAFSLH
O91936      APTLWARIVLMTHFFSVLQSQEQLEKALAFEMYGSVYSVTPLDLPAIIQRLHGLSAFTLH
Q68798      APTIWVRMVLMTHFFGILQPQEQLHKALDFDMYGVTYNITPLDLPQIIQRLHGMAAFSLH
O39928      APTLWARIVLMTHFFSVLQSQEQLEKTLAFEMYGSVYSVTPLDLPAIIQRLHGLSAFSLH
O92531      APAIWVRMVLMTHFFQILQAQEQLDKVLDFDMYGVTYSVSPLQLPAIIQRLHGMAAFSLH
Q68801      APTIWVRMVIMTHFFSILQAQEQLEKALDFEMYGAVYSVTPLDLPAIIERLHGLSAFSLH
Q9QAX1      APTIWVRMVLMTHFFSVLMAQETLDQDLNFEMYGAVYSVNPLDLPAIIERLHGLEAFSLH
O39929      APTIWVRMILMTHFFSILQSQEALEKALDFDMYGVTYSITPLDLPAIIQRLHGLSAFTLH
O92532      APTIWVRMVLMTHFFQILQAQETLDRALDFDIYGVTYSITPLDLPVIIQRLHGMAAFSLH
Q68749      APTVWVRMVLMTHFFSVLMAQDTLDQDLNFEMYGAVYSVSPLDLPAIIERLHGLEAFSLH
Q81487      APTIWVRMVLITHFFSILQAQEQLERALDFEMYGATYSVTPLDLPAIIERLHGLSAFSLH
P26660      APTIWARMVLMTHFFSILMAQDTLDQNLNFEMYGAVYSVSPLDLPAIIERLHGLDAFSLH
P26661      APTIWVRMVIMTHFFSILLAQDTLNQNLNFEMYGAVYSVNPLDLPAIIERLHGLEAFSLH
Q99IB8      APTIWVRMVLMTHFFSILMVQDTLDQNLNFEMYGSVYSVNPLDLPAIIERLHGLDAFSMH
Q9DHD6      APTIWVRMVIMTHFFSILLAQDTLNQNLNFEMYGAVYSVNPLDLPAIIERLHGLDAFSLH
Q81495      APTIWVRMVMMTHFFSILQSQEILDRPLDFEMYGATYSVTPLDLPAIIERLHGLSAFSVH
Q81258      APTIWVRMVMMTHFFSILQSQEILDRPLDFEMYGATYSVTPLDLPAIIERLHGLSAFTLH
Q69422      YPCLWVSRVLAVHFMEQMLFEDKLPETVTFDWYGKNYTVPVEDLPSIIAGVHGIEAFSVV
```

```
                480       490       500       510       520       530
A0A1B2FV88   SYSPGEINRVASCLRKLGVPPLRVWRHRARSVRAKLLSRGGRAATCGKYLFNWAVRTKLK
Q00269       SYSPGEINRVASCLRKLGVPPLRVWRHRARSVRARLLSQGGRAATCGKYLFNWAVRTKLK
P26663       SYSPGEINRVASCLRKLGVPPLRVWRHRARSVRARLLSQGGRAATCGKYLFNWAVKTKLK
P29846       SYSPGEINRVASCLRKLGVPPLRAWRHRARSVRAKLLSQGGRAATCGRYLFNWAVKTKLK
Q913V3       SYSPGEINRVASCLRKLGVPPLRVWRHRARSVRAKLLSQGGRAATCGKYLFNWAVRTKLK
Q9WMX2       SYSPGEINRVASCLRKLGVPPLRVWRHRARSVRARLLSQGGRAATCGKYLFNWAVRTKLK
O92972       SYSPGEINRVASCLRKLGVPPLRTWRHRARSVRAKLLSQGGRAATCGRYLFNWAVRTKLK
P26662       SYSPGEINRVASCLRKLGVPPLRVWRHRARSVRAKLLSQGGRAATCGKYLFNWAVKTKLK
Q81754       SYSPGEINRVAACLRKLGVPPLRAWRHRARSVRATLLSQGGRAAICGKYLFNWAVKTKLK
Q03463       SYSPGEINRVAACLRKLGVPPLRAWRHRARSVRARLLSRGGRAAICGKYLFNWAVRTKLK
P26664       SYSPGEINRVAACLRKLGVPPLRAWRHRARSVRARLLARGGRAAICGKYLFNWAVRTKLK
P27958       SYSPGEINRVAACLRKLGVPPLRAWRHRAWSVRARLLARGGKAAICGKYLFNWAVRTKLK
Q913D4       SYSPGEINRVAACLRKLGVPPLRAWRHRARSVRATLLSQGGKAAICGKYLFNWAVKTKLK
Q5I2N3       GYSPTELNRVGACLRKLGVPPLRAWRHRARAVRAKLIAQGGKAAICGKYLFNWAVKTKLK
O39927       GYSPVELNRVGACLRKLGVLPSRAWRHRARAVRAKLIAQGGKAAICGKYLFNWAVKTKLK
O92530       GYSPGELNRVASCLRKLGAPPLRAWRHRARAVRAKLIAQGGKHAICGKYLFNWAVRTKLK
O92529       GYSPTELNRVGASLRKLGAPPLRAWRHRARAVRAKLIAQGGKAAICGKYLFNWAVKTKLK
O91936       SYSPSEINRVSSCLRKLGVPPLRAWRHRARAVRAKLIAQGGKAAICGIYLFNWAVKTKRK
Q68798       GYSPGELNRVGACLRKLGAPPLRAWRHRARAVRAKLIAQGGKAAICGMYLFNWAVKTKLK
O39928       SYSPSEINRVASCLRKLGVPPLRAWRHRARAVRAKLIAQGGRAAICGIYLFNWAVKTKRK
O92531       GYSPTELNRVGACLRKLGAPPLRAWRHRARAVRAKLIAQGGGAAICGKYLFNWAVKTKLK
Q68801       SYSPVELNRVAGALRKLGIPPLRAWRHRARAVRAKLISQGGKAKICGLYLFNWAVRTKAK
Q9QAX1       GYSPTELTRVAAALRKLGAPPLRAWKSRARAVRASLISQGGRAATCGFYLFNWAVRTKRK
O39929       GYSPHELNRVAGALRKLGVPPLRAWRHRARAVRAKLIAQGGRAKICGIYLFNWAVKTKLK
O92532       GYSPDELNRVASCLRKLGAPPLRAWRHRARAVRAKLIAQGGKAAVCGKYLFNWAIKTKLR
Q68749       SYSPHELTRVAAALRKLGAPPLRAWKSRARAVRASLISRGGSAATCGRYLFNWAVRTKLK
Q81487       GYSPTELNRVAGALRKLGIPPLRAWRHRARAVRAKLIAQGGKARICGLYLFNWAVRTKTK
P26660       TYTPHELTRVASALRKLGAPPLRAWKSRARAVRASLISRGGRAAVCGRYLFNWAVKTKLK
P26661       TYSPHELSRVAATLRKLGAPPLRAWKSRARAVRASLIAQGARAAICGRYLFNWAVKTKLK
Q99IB8       TYSHHELTRVASALRKLGAPPLRVWKSRARAVRASLISRGGKAAVCGRYLFNWAVKTKLK
Q9DHD6       TYSPHELSRVAATLRKLGAPPLRAWKSRARAVRASLIIQGGRAATCGRYLFNWAVKTKLK
Q81495       SYSPVELNRVAGTLRKLGCPPLRAWRHRARAVRAKLIAQGGRAKICGLYLFNWAVRTKTK
Q81258       SYSPVELNRVAGTLRKLGCPPLRAWRHRARAVRAKLIAQGGKAKICGLYLFNWAVRTKTN
Q69422       RYTNAEILRVSQSLTDMTMPPLRAWRKKARAVLASAKRRGGAHAKLARFLL.WHATSR..
```

```
                540       550       560       570       580       590
A0A1B2FV88   LTPIPAASQLDLSSWFVAGYSG.....GDIYHSVSRARPRWLMLCLLLSVGVGIYLLPN
Q00269       LTPIPAASQLDLSSWFVAGYSG.....GDIYHSLSRARPRWFMWCLLLSVGVGIYLLPN
P26663       LTPIPAASRLDLSGWFVAGYSG.....GDIYHSLSRARPRWFMLCLLLSVGVGIYLLPN
P29846       LTPIPAASQLDLSKWFVAGYGG.....GDIYHSLSRARPRWFMLCLLLSVGVGIYLLPN
Q913V3       LTPIPAASQLDLSSWFVAGYSG.....GDIYHSLSRARPRWFMLCLLLSVGVGIYLLPN
Q9WMX2       LTPIPAASQLDLSSWFVAGYSG.....GDIYHSLSRARPRWFMWCLLLSVGVGIYLLPN
O92972       LTPIPAASQLDLSGWFVAGYSG.....GDIYHSLSRARPRWFPLCLLLSVGVGIYLLPN
P26662       LTPIPAASQLDLSGWFVAGYNG.....GDIYHSLSRARPRWFMLCLLLSVGVGIYLLPN
Q81754       LTPLPSASQLDLSNWFTGGYSG.....GDIYHSVSHVRPRWFFWCLLLSVGVGIYLLPN
Q03463       LTPIAAAGRLDLSGWFTAGYSG.....GDIYHSVSHARPRWFWFCLLLLAAGVGIYLLPN
P26664       LTPIAAAGQLDLSGWFTAGYSG.....GDIYHSVSHARPRWIWFCLLLLAAGVGIYLLPN
P27958       LTPITAAGRLDLSGWFTAGYSG.....GDIYHSVSHARPRWFWFCLLLLAAGVGIYLLPN
Q913D4       LTPLPSMSQLDLSNWFTGGYSG.....GDIYHSVSHARPRLFLWCLLLSVGVGIYLLPN
Q5I2N3       LTPLVSASKLDLSGWFVAGYDG.....GDIYHSVSQARPRLLLLGLLLTVGVGIFLVPA
O39927       LTPLVSASKLDLSGWFVAGYDG.....GDIYHSVSQARPRFLLLGLLLTVGVGIFLLPA
O92530       LTPLRGAANLDLSGWFVSGGSG.....GDIFHSVSRARPRNLLLCLLLTVGVGIFLLPA
O92529       LTPLAAASQLDLSGWFVAGYDG.....GDIYHSVSRARPRLLLLGLLLTVGVGIFLLPA
O91936       LTPLADADRLDLSSWFTVGAGG.....GDIYHSMSRARPRCILLCLLLTVGVGIFLLPA
Q68798       LTPLRDAHRLDLSGWFVAGYSG.....GDIFHSVSHARPRVLLLCLLLTVGVGIFFLPP
O39928       LTPLADADRLDLSSWFTVGAGG.....GDIYHSMSRARPRNLLLCLLLSVGVGIFLLPA
O92531       LTPIPDAARLDLSGWFISGFSG.....GDIYHSVSRARPRIFLLCLLLSVGVGIFLLPA
Q68801       LTPLPQAGLLDLSRWFTVGAGG.....NDIYHSVSRARSRHLLLGLLLTVGVGIFLLPA
Q9QAX1       LTPLPAARRLDLSGWFTVGAGG.....GDIYHSVSRARPRFLLLCLLLSVGVGIFLLPA
O39929       LTPLPAAKLDLSGWFTVGAGG.....GDIYHSMSHARPRYLLLCLLILTVGVGIFLLPA
O92532       LTPLRGASALDLSGWFTSGYGG.....GDVYHSASRARPRFLLLCLLLSVGVGIFLLPA
Q68749       LTPLPAARLLDLSSWFTVSAGG.....GDIYHSVSRARPRLLLLGLLLCVGVGIFLLPA
Q81487       LTPLPTAGQLDLSSWFTVGVGG.....NDIYHSVSRARTRHLLLCLLLLTVGVGIFLLPA
P26660       LTPLPEARLLDLSSWFTVGAGG.....GDIYHSVSRARPR.....................
P26661       LTPLPEASRLDLSGWFTVGAGG.....GDIYHSVSHARPRLLLLCLLLSVGVGIFLLPA
Q99IB8       LTPLPEARLLDLSSWFTVGAGG.....GDIFHSVSRARPR.....................
Q9DHD6       LTPLPEASRLDLSGWFTVGAGG.....GDIFHSVSHARPRLLLLCLLLSVGVGIFLLPA
Q81495       LTPLPAAGQLDLSSWFTVGVGG.....NDIYHSVSRARTRYLLLCLLLLTVGVGIFLLPA
Q81258       LTPLPAAGQLDLSSWFTVGVGG.....NDIYHSVSRARTRHLLLCLLLLTVGVGIFLLPA
Q69422       ..PLPDLDKTSVARYTTFNYCDVYSPEGDVFVTPQRRLQKFLVKYLAVIVFALGL.....
```

A0A1B2FV88	R
Q00269	R
P26663	R
P29846	R
Q913V3	R
Q9WMX2	R
O92972	R
P26662	R
Q81754	R
Q03463	R
P26664	R
P27958	R
Q913D4	R
Q5I2N3	R
O39927	R
O92530	R
O92529	R
O91936	R
Q68798	R
O39928	R
O92531	R
Q68801	R
Q9QAX1	R
O39929	R
O92532	R
Q68749	R
Q81487	R
P26660	.
P26661	R
Q99IB8	.
Q9DHD6	R
Q81495	R
Q81258	R
Q69422	.
